# Design, Synthesis, and Anticancer Activity of Novel Enmein-Type Diterpenoid Derivatives Targeting the PI3K/Akt/mTOR Signaling Pathway

**DOI:** 10.3390/molecules29174066

**Published:** 2024-08-27

**Authors:** Jiafeng Wang, Lu Wang, Yingbo Zhang, Siwen Pan, Yu Lin, Jiale Wu, Ming Bu

**Affiliations:** 1 College of Pathology, Qiqihar Medical University, Qiqihar 161006, China; wangjiafeng410323@163.com (J.W.); zhangyingbo0930@163.com (Y.Z.); siwen_tongtong0606@163.com (S.P.); 2 College of Pharmacy, Qiqihar Medical University, Qiqihar 161006, China; wanglu9264582022@163.com (L.W.); linyu7373@163.com (Y.L.); 3 College of Life and Health, Hainan University, Haikou 570228, China; wwwwjl55@163.com

**Keywords:** enmein-type *ent*-Kauranes, derivatives, structural modification, anticancer, PI3K/Akt/mTOR pathway

## Abstract

The enmein-type diterpenoids are a class of anticancer *ent*-Kaurane diterpnoids that have received much attention in recent years. Herein, a novel 1,14-epoxy enmein-type diterpenoid **4**, was reported in this project for the first time. A series of novel enmein-type diterpenoid derivatives were also synthesized and tested for anticancer activities. Among all the derivatives, compound **7h** exhibited the most significant inhibitory effect against A549 cells (IC_50_ = 2.16 µM), being 11.03-folds better than its parental compound **4**. Additionally, **7h** exhibited relatively weak anti-proliferative activity (IC_50_ > 100 µM) against human normal L-02 cells, suggesting that it had excellent anti-proliferative selectivity for cancer cells. Mechanism studies suggested that **7h** induced G0/G1 arrest and apoptosis in A549 cells by inhibiting the PI3K/AKT/mTOR pathway. This process was associated with elevated intracellular ROS levels and collapsed MMP. In summary, these data identified **7h** as a promising lead compound that warrants further investigation of its anticancer properties.

## 1. Introduction

Cancer, the disease of abnormal cell proliferation, stands as one of the principal threats to human life in the 21st century [1]. According to recent statistics, the global cancer-related death toll reached 10 million in 2020, with lung cancer claiming the highest number of lives, accounting for 18.0% of total cancer deaths, followed by liver cancer (8.3%) and stomach cancer (7.7%) [2]. The global cancer mortality rate is increasing annually, leading to an estimated 16 million future deaths per year by 2040 [3]. With advancements in science and technology, state-of-the-art diagnostic techniques and therapeutic methods have been applied to control various types of cancers, improving the survival of patients to some extent [4,5,6,7]. Chemotherapy is one of the broadest cancer treatment strategies in clinical application, but the conventional chemotherapeutic agents are often prone to developing cancer cell resistance or causing serious poisonous side effects to patients [8]. This urgent health crisis is propelling the need for innovative therapeutic agents with higher targeting efficiency and minimal side effects [9,10].

Natural products are a precious gift from nature to humankind, playing an irreplaceable role in drug discovery [11]. On the one hand, natural products can bind easily with biomolecules in cells and outwardly exhibit diverse biological activities [12,13]. On the other hand, natural products have the characteristic of structural diversity, which can provide rich sources of reference for the design of small-molecule drugs [14,15,16]. At present, more than 50% of clinical anticancer drugs are derived directly or indirectly from natural products [17,18,19].

**Figure 1:**
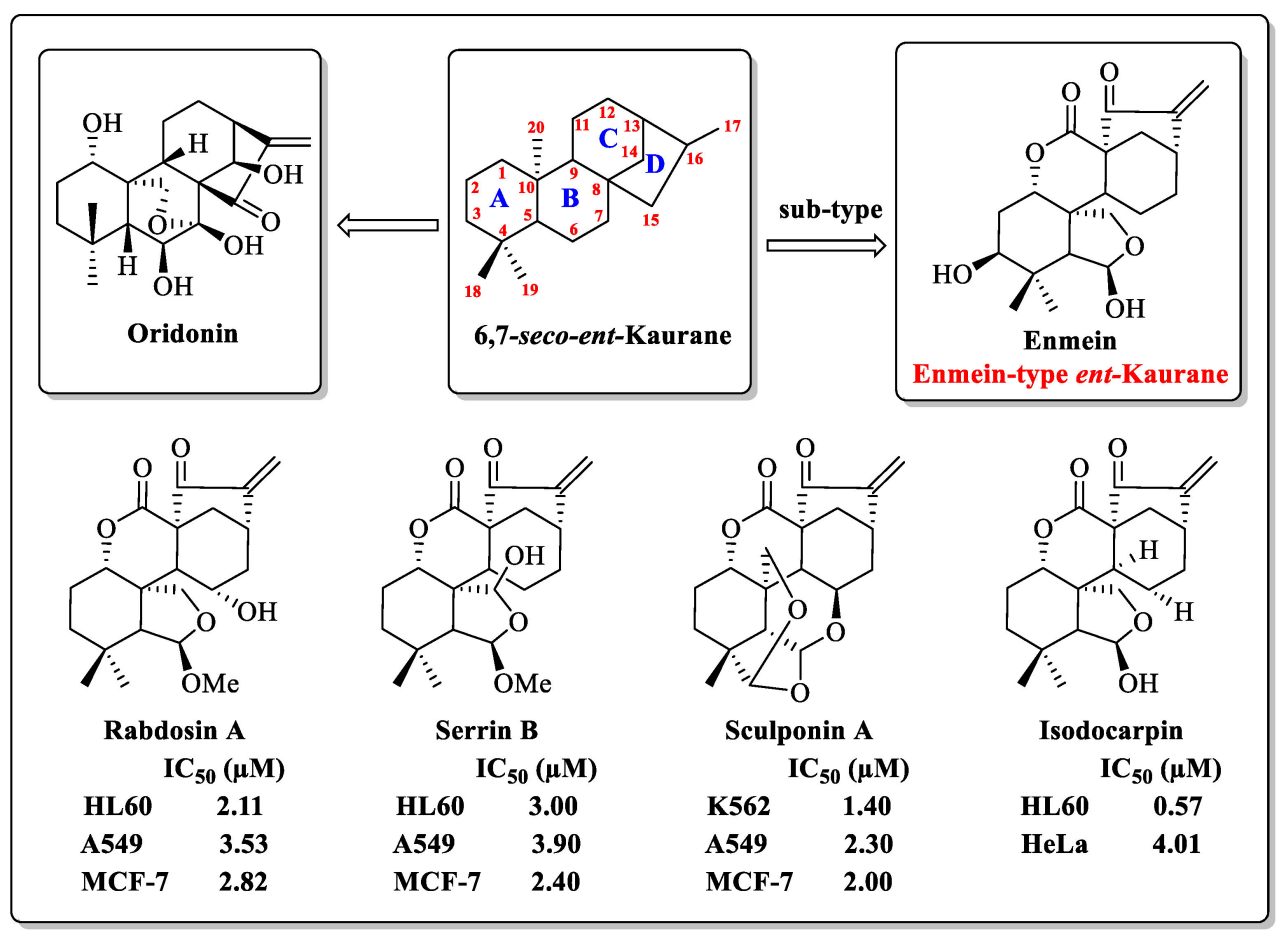
The structure of oridonin and partial enmein-type dipterpenoids.

*ent*-Kaurane diterpenoids, a widespread natural tetracyclic diterpenoid product, have received much attention because of their wide range of biological activities [20,21]. Oridonin (**1**, Figure 1), the most well-known natural *ent*-Kaurane diterpenoid product, has been reported in numerous studies for its inhibition of various cancer cells [22,23,24,25]. The enmein-type (1,7-lactone) diterpenoid is a sub-type of the *ent*-Kaurane diterpenoids. Partial enmein-type diterpenoids, such as Enmein, Rabdosin A, Sculponin A, Serrin B, and Isodocarpin (Figure 1), have shown micro-molar inhibitory effects against various cancer cells in vitro [26,27,28,29]. Although they demonstrate attractive anticancer potential, their lower natural yields have limited their further research and application. To solve this problem, Xu’s team constructed a semi-synthetic strategy for the synthesis of enmein-type diterpenoids from odidonin, and also obtained a variety of derivatives with potential for clinical anticancer applications [30]. In summary, enmein-type diterpenoids and their derivatives have the potential to be developed as anticancer drugs for clinical use.

To diversify the enmein-type diterpenoids and explore their potential anticancer mechanisms, a novel 1,14-epoxy enmein-type diterpenoid was designed and synthesized. After that, a compound library containing 21 novel enmein-type diterpenoid derivatives was also constructed in this project. The anticancer activities of all derivatives were tested via MTT assay, and their structure–activity relationships (SARs) were summarized. As the lead compound with the most potential, **7h** was selected for further investigation of its anticancer mechanisms.

## 2. Results and Discussion

### 2.1. Synthesis of Enmein-Type Diterpenoid Derivatives

**Scheme 1:**
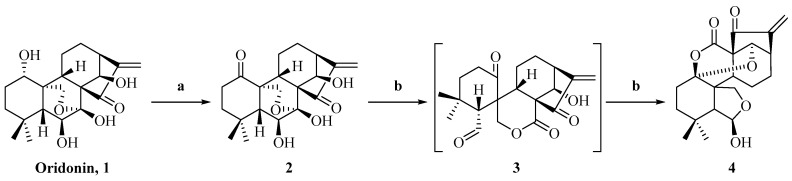
Synthesis of enmein-type diterpenoid derivative **4**. Reagents and conditions: (a) Acetone, Jones Reagent, 0 °C, 1 h; (b) THF, anhydrous sodium carbonate, lead tetraacetate, 25 °C, 1 h.

**Figure 2:**
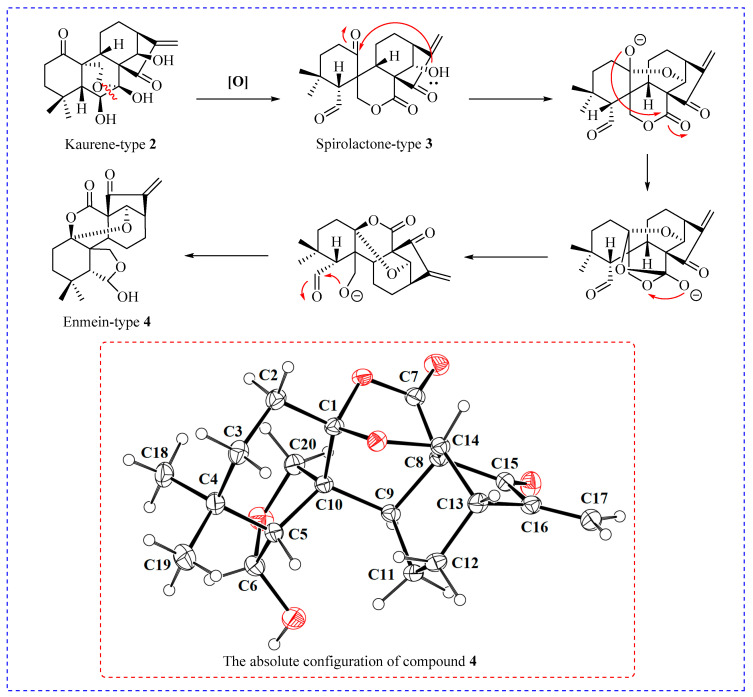
The potential mechanism of conversion from compound **2** to enmein-type diterpenoid **4,** and the absolute configuration of compound **4**.

The synthesis of enmein-type diterpenoids is outlined in Scheme 1. Oridonin, as the starting material, was oxidised by Johns’ reagent at 0 °C to synthesize the *ent*-Kaurene **2** [30]. Compound **2** was oxidized by lead tetraacetate and anhydrous sodium carbonate to produce the spironolactone-type diterpenoid **3** [30]; after that, through further advancing the reaction, compound **3** was converted to the enmein-type diterpene compound **4**. The assumed mechanisms of conversion from compound **2** to spironolactone-type diterpenoid **3** and enmein-type diterpenoid **4** are shown in Figure 2. The chemical structure of compound **4** was confirmed by HRMS, ^1^H NMR, ^13^C NMR, and crystal data.

**Scheme 2:**
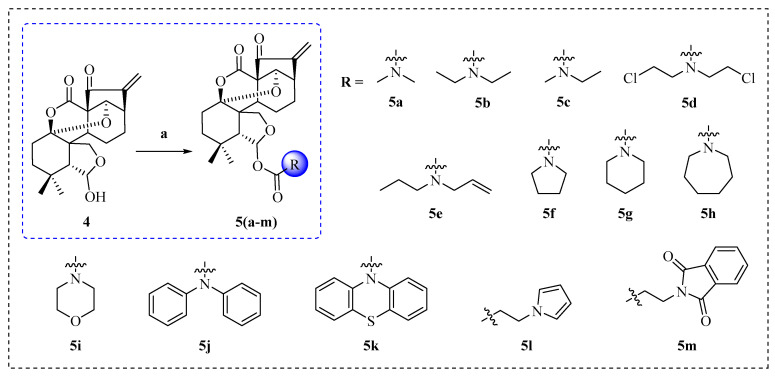
Synthesis of enmein-type diterpenoid derivatives **5a**–**m**. Reagents and conditions: (a) Acyl chlorides, DCM, TEA, DMAP, 25 °C, 24 h.

The cationic amino group, including carbamates, which are commonly used in the structural modification of natural products, can facilitate the binding between small molecules and protein receptors through electrostatic interactions, and further enhance the bio-activities of natural products [31,32]. We designed a series of novel derivatives, **5a**–**m**, synthesized by introducing different amino acid esters at the C-6 position of compound **4** via acylation reaction (Scheme 2). The chemical structures of all derivatives, **5a**–**m**, were determined by HRMS, ^1^H NMR, and ^13^C NMR spectra data.

**Scheme 3:**
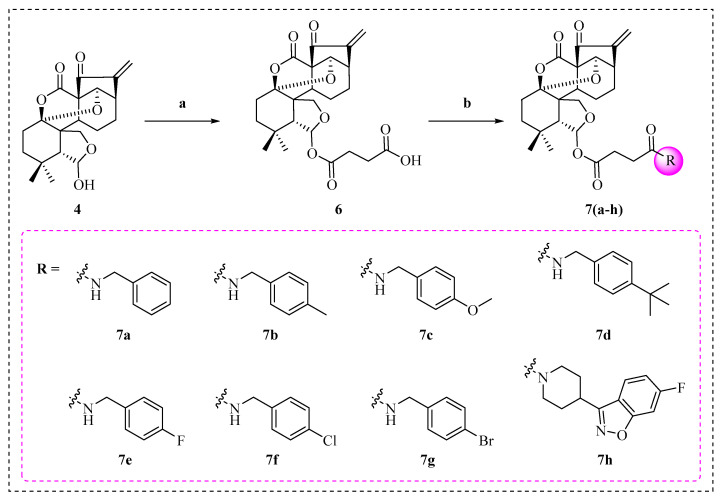
Synthesis of enmein-type diterpenoid derivatives **7a**–**h**. Reagents and conditions: (a) Amines, DMAP, DCM, 25 °C, 4 h; (b) suitable substituent, DCM, PyBOP, TEA, 25 °C, 3 h.

To explore whether a flexible side chain containing aromatic groups could enhance the anticancer activities of enmein-type diterpenoids, a series of novel derivatives, **7a**–**h**, were designed and synthesized (Scheme 3). Compound **4** and succinic anhydride were catalyzed by 4-dimethylaminopyridine (DMAP) to form intermediate compound **6** [33,34]. After that, compound **6** was acylated with different amines to obtain the target products, **7a**–**h**. The chemical structures of all derivatives were determined by HRMS, ^1^H NMR, and ^13^C NMR spectral data.

### 2.2. In Vitro Anti-Proliferative Activity and SAR Analysis

A series of novel enmein-type diterpenoid derivatives, **5a**–**m**, were synthesized by introducing a side chain containing a carbamate moiety at the C-6 position of compound **4**. The anticancer activities of **5a**–**m** were tested against three human cancer cell lines and one human normal cell line by MTT assay. The results are presented in Table 1.

In A549 cells, all of the derivatives, **5a**–**m**, exhibited better anticancer activities than their parental compound **4** (IC_50_ = 23.83 µM). Among them, compound **5m** (IC_50_ = 6.85 µM), which contained an indole moiety, displayed the most remarkable anticancer activity, which was 3.48-fold better than that of compound **4**. For the HepG2 cells and the MCF-7 cells, most derivatives showed better anticancer activities compared with their parental compound **4**; however, overall, neither reached micro-molar levels (IC_50_ < 10.00 µM). It is noteworthy that the derivatives containing both longer flexible linker arms and aromatic groups in their side chains, such as **5l** and **5m**, exhibited better anticancer activities than other derivatives. However, the derivatives containing only aliphatic chain groups (**5d**, **5e**) or aromatic groups (**5j**, **5k**) in their side chains failed to exhibit anticancer activities at the low micromolar level. We assumed that the flexible linker arms and aromatic groups in the side chains are indispensable for the derivatives’ enhanced anticancer activities.

To verify the above conjecture, a series of novel derivatives, **7a**–**h**, were synthesized using coupling compound **4** and aromatic groups, using succinic acid as a linker. The anticancer activities of **7a**–**h** were tested against three human cancer cell lines and one human normal cell line by MTT assay. The results are also presented in Table 1.

In A549 cells, all derivatives, **7a**–**h**, exhibited better anticancer activities than compound **4**, and all attained low micro-molar levels (IC_50_ < 10.00 µM). Among them, derivative **7h** exhibited the best anticancer activity (IC_50_ = 2.16 µM), which was 11.03-fold better than compound **4**, and which was better than the positive control taxol. Furthermore, **7h** also exhibited a relatively weak cytotoxic activity (IC_50_ > 50 µM) against human normal L-02 cell lines, indicating an excellent cancer cell-targeting property. For HepG2 cells, compounds **7f**, **7g**, and **7h** showed low micro-molar-level anticancer activities. For MCF-7 cells, all compounds except compounds **7a**, **7b**, and **7g** showed low micro-molar-level anticancer activities. These data suggest that the introduction of a suitable linking arm between the diterpenoid parental nucleus and the active moiety can remarkably improve the anticancer activities of derivatives.

Taken together with a suitable linking arm, derivative **7h**, which was successively substituted with succinic acid and 6-fluoro-3-(piperidin-4-yl) benzo[*d*]isoxazole groups, exhibited low micro-molar-level inhibitory effects against all tested cancer cells, suggesting that it is a potential lead compound for clinical anticancer applications. Therefore, compound **7h** was selected for further anticancer-mechanism exploration in A549 cells.

### 2.3. Cell Cycle Analysis

**Figure 3:**
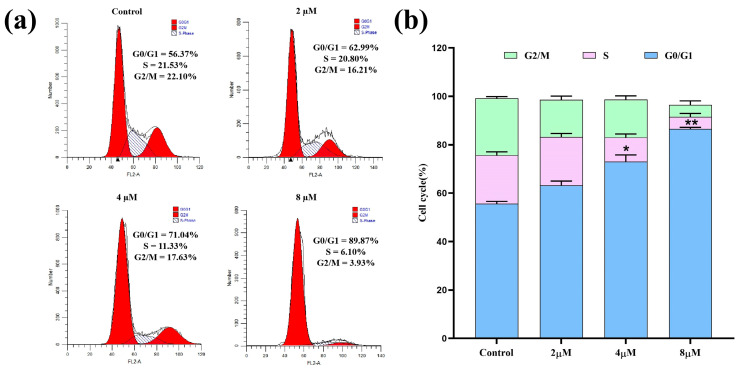
Compound **7h** induced cell cycle arrest in A549 cells. (**a**) A549 cells were treated with **7h** (2, 4, and 8 µM) and 0.1% DMSO (the blank control group), and stained by PI/RNase. (**b**) The percentage of cell cycle arrest in different experimental groups (*: *p* < 0.05, **: *p* < 0.01, compared to the blank control group). All data are expressed as mean ± standard deviation (n = 3).

Cell cycle regulation is an essential process in maintaining normal bodily functions and is often disturbed in cancer cells. The inducement of cell cycle arrest is considered a key avenue for targeted cancer therapy [35]. As a novel enmein-type diterpenoid derivative with remarkable anti-proliferative effects against A549 cells, **7h** may have the ability to induce cell cycle arrest. Therefore, A549 cells were treated with **7h**, stained with PI/RNase solution, and analyzed for their cell cycle distribution by flow cytometry. The experimental results are shown in Figure 3. The detailed data revealed that, under the treatments with different concentrations (0, 2, 4, and 8 µM) of **7h**, 56.37%, 62.99%, 71.04%, and 89.87% of A549 cells, respectively, were found to be in the G0/G1 phase. The above-mentioned results demonstrate that **7h** effectively inhibited the proliferation of A549 cells by arresting the cell cycle at the G0/G1 phase, and this effect also showed a marked dose-dependent trend.

### 2.4. Apoptosis Analysis

**Figure 4:**
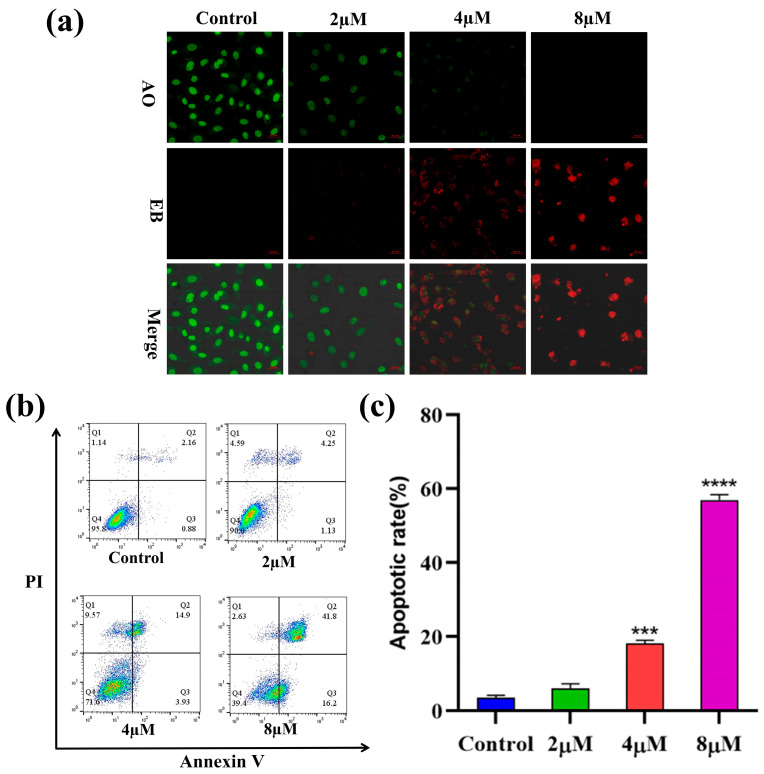
Compound **7h** induced A549 cell apoptosis. (**a**) A549 cells were treated with **7h** (2, 4, and 8 µM) and 0.1% DMSO (blank control), and then stained by AO/EB. (**b**) A549 cells were treated with **7h** (2, 4, and 8 µM) and 0.1% DMSO (the blank control group), and then stained by Annexin V-FITC/PI. (**c**) The percentage of apoptosis in different experimental groups (***: *p* < 0.001, ****: *p* < 0.0001, compared to the blank control group). All data are expressed as mean ± standard deviation (n = 3).

To explore whether compound **7h** could induce apoptosis in cancer cells, A549 cells were treated with different concentrations (0, 2, 4, and 8 µM) of **7h** and then stained with AO/EB double staining. The morphological variations in the cells were observed using fluorescence microscopy, and the results are shown in Figure 4a. In the blank control group, normal cells were stained green with AO staining solution with intact nuclei. With the increased concentration of **7h**, the cells gradually produced typical morphological transformations, such as nuclear chromatin condensation, cytoplasmic condensation, cell fragmentation, etc., and generated large amounts of apoptotic cells (EB staining in red).

To further confirm the capacity of **7h** to induce apoptosis in cancer cells, A549 cells were treated with **7h**, detected using Annexin V-FITC/PI staining, and the apoptosis rate was detected by flow cytometry. The results showed that the percentage of total apoptosis in A549 cells treated with different concentrations of **7h** increased from 5.38% to 58.00%, as opposed to an increase of 3.04% in the control group (Figure 4b,c). Among the types of apoptosis which occurred, late apoptosis was the main form in all apoptosis cells, increasing from 2.16% to 41.8%. Notably, the percentage of apoptosis in the A549 cells was correlated with **7h** in a positively dose-dependent manner. The above results indicated that compound **7h** could significantly induce apoptosis in A549 cells.

### 2.5. Reactive Oxygen Species (ROS) Analysis

**Figure 5:**
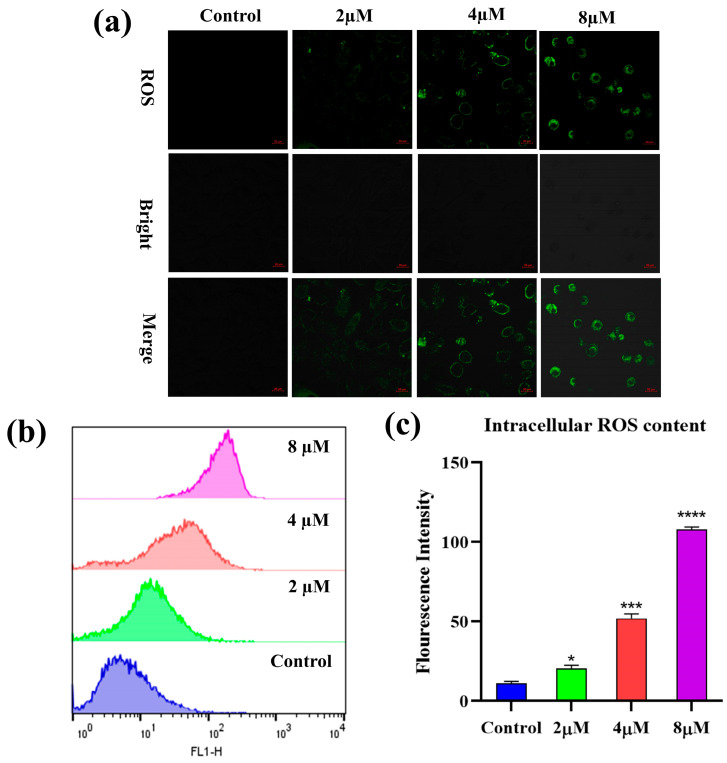
Compound **7h** induced ROS generation in A549 cells. (**a**,**b**) A549 cells were treated with **7h** (2, 4, and 8 µM) and 0.1% DMSO (blank control), and then stained by H2DCFDA. (**a**) The morphological detection of A549 cells. (**b**) The determination of ROS content in A549 cells. (**c**) The percentage of ROS content in different experimental groups (*: *p* < 0.05, ***: *p* < 0.001, ****: *p* < 0.0001, compared to the blank control group). All data are expressed as mean ± standard deviation (n = 3).

Reactive oxygen species (ROS) are secondary metabolites of cells and are closely linked to the regulation of multiple biological activities. Elevated intracellular ROS levels are thought to be a hallmark of apoptosis [35]. To determine whether **7h** could affect the ROS content in cancer cells, the A549 cells were treated with different concentrations (0, 2, 4, and 8 µM) of **7h** and then stained with H2DCFDA. The results of the laser scanning confocal microscopy observations that we conducted on the stained cells are shown in Figure 5a. Compared with the blank control group, all experimental groups treated with **7h** were observed to exhibit remarkable green fluorescence, and the fluorescence intensity was positively correlated with the concentration of **7h** in a dose-dependent manner.

Flow cytometry was used to detect changes in intracellular reactive oxygen species content, and the results are shown in Figure 5b,c. The intracellular ROS content of the **7h**-treated A549 cells was increased significantly in a dose-dependent manner compared to the control group. The above results suggest that **7h**-induced apoptosis in A549 cells may be involved in the notable induction of intracellular ROS production.

### 2.6. Mitochondrial Membrane Potential (MMP) Analysis

**Figure 6:**
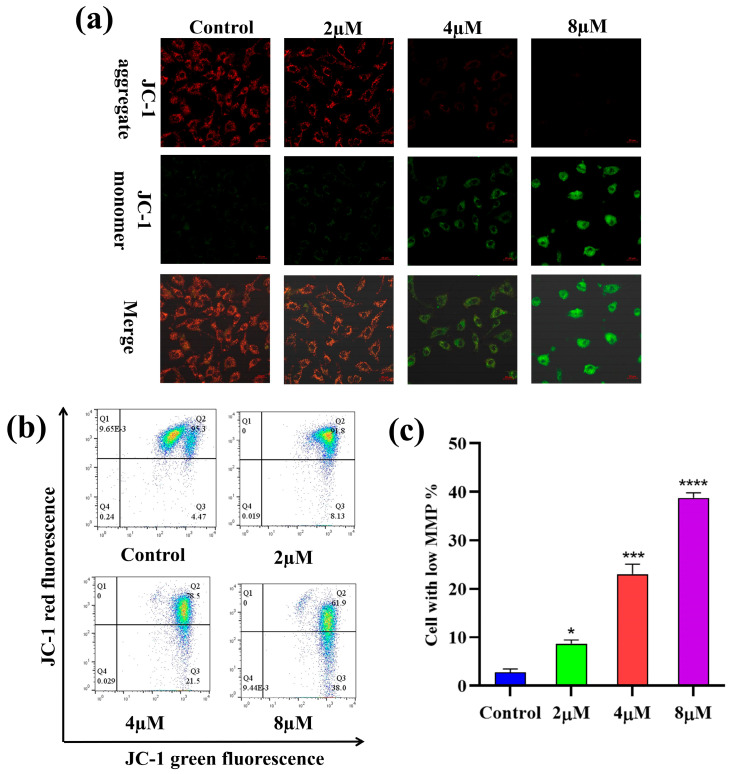
Compound **7h** induced MMP in A549 cells. (**a**,**b**) A549 cells were treated with **7h** (2, 4, and 8 µM) and 0.1% DMSO (blank control), and then stained by JC-1. (**a**) The morphological detection of A549 cells. (**b**) The determination of MMP depolarization rate in A549 cells. (**c**) The percentage of MMP depolarization rate in different experimental groups (*: *p* < 0.05, ***: *p* < 0.001, ****: *p* < 0.0001, compared to blank the control group). All data are expressed as mean ± standard deviation (n = 3).

The depolarization of MMP is reckoned to be a hallmark of apoptosis [36]. To investigate the impact of **7h** on the depolarization of MMP (*δ*:*Ψm*) in cancer cells, A549 cells were treated with different concentrations (0, 2, 4, and 8 µM) of **7h** and then stained with JC-1. The laser confocal microscopy observations are shown in Figure 6a. In the blank control group, most cells were stained red with JC-1, indicating a high mitochondrial membrane potential. In contrast, in the **7h**-treated experimental groups, the number of red cells decreased while the number of green cells increased, indicating a significant reduction in intracellular MMP. The results of the flow cytometry assay are illustrated in Figure 6b,c.

The results showed that the percentage of cells with depolarized MMP increased in a dose-dependent manner after the A549 cells were treated with different concentrations (0, 2, 4, and 8 µM) of **7h**, from 3.24% to 15.21%, 25.6%, and 59.6%, respectively. Altogether, these findings suggest that **7h**-induced cell cycle arrest and apoptosis in A549 cells may be linked to the depolarization of intracellular MMP.

### 2.7. The Predicted Potential Targets and Pathways of ***7h***

To further explore the anticancer mechanism of compound **7h**, we predicted potential targets by molecular docking in the PharmMapper platform [37,38]. A total of 106 target proteins (Z-score > 0.5) were selected (Table S1). The Gene Ontology (GO) enrichment and Kyoto Encyclopedia of Genes and Genomes (KEGG) enrichment results were analyzed using the David platform [39,40].

**Figure 7:**
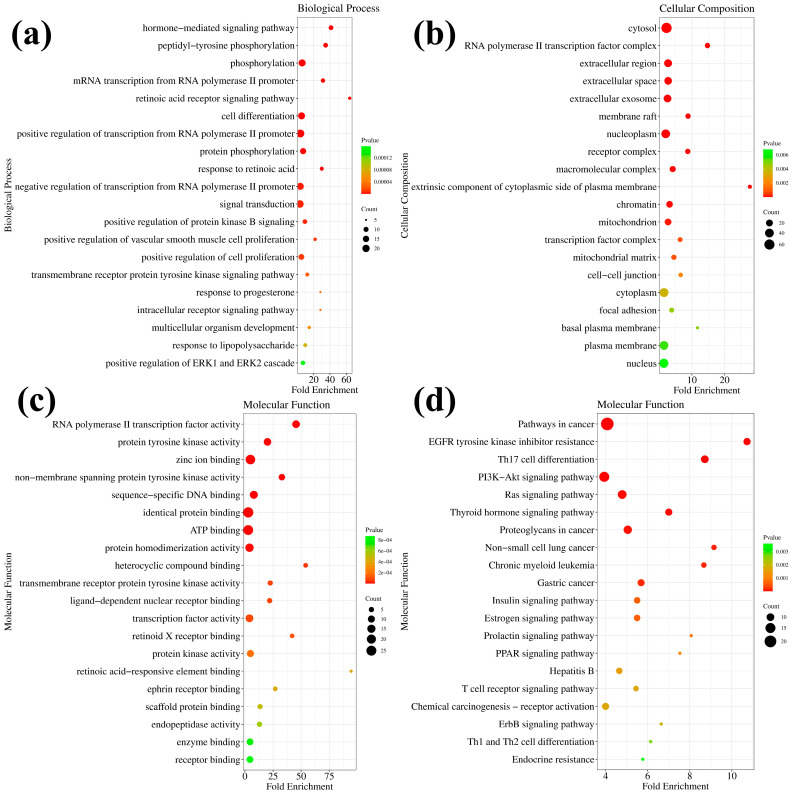
GO and KEGG enrichment analysis of **7h**. (**a**) The predicted molecular functions of **7h** based on GO analysis. (**b**) The predicted biological processes of **7h** based on GO analysis. (**c**) The predicted cellular compositions of **7h** based on GO analysis. (**d**) The predicted pathways of **7h** based on KEGG analysis.

GO enrichment analysis was performed against **7h** to predict its potential biological processes (BP), cellular composition (CC), and molecular functions (MF). The top 20 functional categories (the minimum of the *p*-value) were screened separately. Compound **7h** in BP mainly involves the hormone-mediated signaling pathway, phosphorylation, RNA polymerase II promoter protein phosphorylation, cell differentiation, signal transduction, and others (Table S2, Figure S1, and Figure 7a). Compound **7h** in CC is mainly located in the cytosol, RNA polymerase II transcription factor, extracellular space, nucleoplasm, and others (Table S3, Figure S2 and Figure 7b). Compound **7h** in MF mainly involves zinc ion binding, identical protein binding, ATP binding, protein homodimerization activity, protein tyrosine kinase activity, and others (Table S4, Figure S3 and Figure 7c). KEGG enrichment analysis was performed against **7h** and the top 20 potential pathways were screened (Table S5, Figure S4 and Figure 7d). The prediction suggests that the main effects of compound **7h** may be on cancer-related pathways and proteins in cells, potentially including the PI3K-Akt signaling pathway, the Ras signaling pathway, proteoglycans, the thyroid hormone signaling pathway, and others. The PI3K-Akt signaling pathway is proposed to be one of the potential major anticancer pathways of compound **7h**. Taken together with compound **7h**, the PI3K-Akt signaling pathway was selected for further studies to elucidate the exact anticancer mechanism of compound **7h**.

### 2.8. Western Blot Analysis

**Figure 8:**
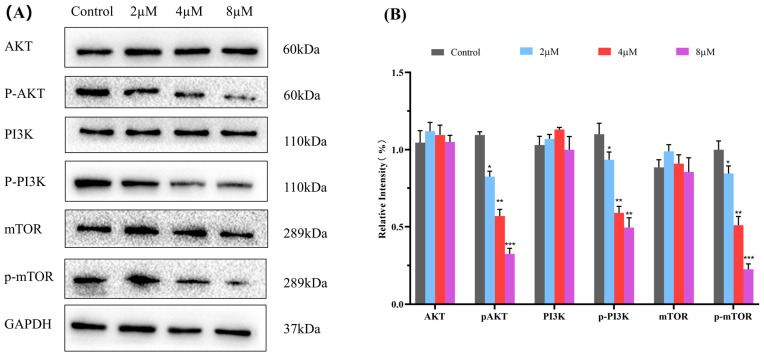
Compound **7h** inhibited the PI3K/AKT/mTOR pathway. (**A**) Western blot analysis of the expression levels of PI3K, p-PI3K, Akt, p-AKT, mTOR, and p-mTOR proteins in A549 cells treated with **7h** (2, 4, and 8 µM) and 0.1% DMSO (the blank control group). (**B**) Statistical analysis. *: *p* < 0.05, **: *p* < 0.01, ***: *p* < 0.001, compared to blank the control group. All data are expressed as mean ± standard deviation (n = 3).

The PI3K/Akt/mTOR pathway is a critical intracellular signaling pathway during cancer development that regulates cellular processes such as proliferation, differentiation, apoptosis, and autophagy. Inhibiting the aberrant activation of the PI3K/Akt/mTOR signaling pathway is envisaged as a promising anticancer strategy [41,42,43]. Based on KEGG analysis and researching the literature, the PI3K/Akt/mTOR signaling pathway is predicted to be one of the major pathways for **7h** to exert its anticancer effects. Therefore, a western blot assay was performed to determine the expression of the main proteins, PI3K, p-PI3K, Akt, p-Akt, mTOR, and p-mTOR, to examine whether the anticancer ability of **7h** against A549 cells is related to its modulation of the PI3K/Akt/mTOR pathway. The results demonstrated that **7h** can effectively reduce the phosphorescent expression levels of p-PI3K and p-mTOR in A549 cells compared with the blank control in a dose-dependent manner (Figure 8). In addition, **7h** also showed an effect on Akt protein phosphorylation expression. The above results suggest that **7h** can exert significant anticancer effects by modulating the PI3K/Akt/mTOR signaling pathway in A549 cells.

### 2.9. Molecular Docking Study

To explore the binding ability between ligand compounds and potential receptor proteins, compound **7h** and its parental compound **4** were subjected to molecular docking studies with the target proteins PI3Kα (PDB ID: 6GVF), Akt1 (6HHF), and mTOR (4JT6), respectively [44,45]. The docking results are shown in Table 2. The docking study results showed that compound **7h** exhibited superior binding ability to all three target proteins compared to its parental compound **4**. Among them, compound **7h** had the lowest docking binding energy (−11.6 kcal/mol) with the PI3Kα protein, which represented the highest binding affinity; therefore, PI3Kα might be one of the main targets of action of compound **7h**.

**Figure 9:**
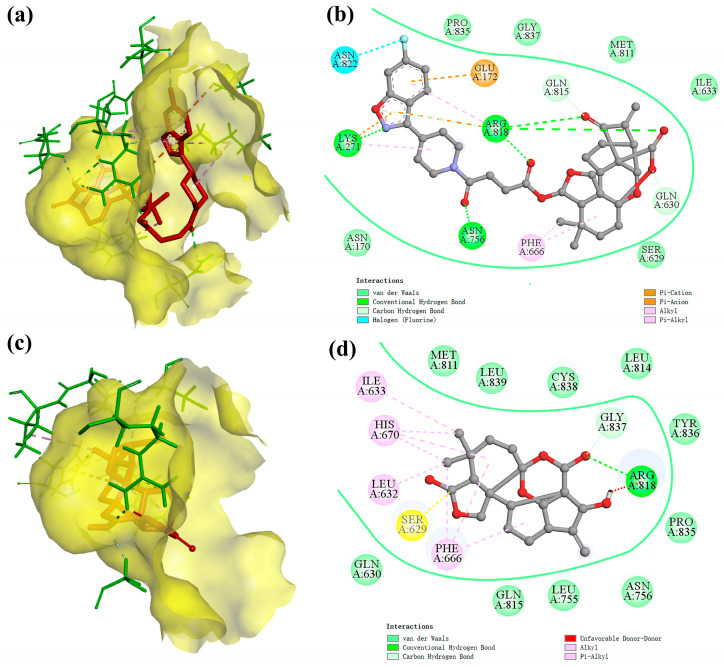
The docking model of ligand molecules to receptor proteins. (**a**) The 3D interaction model of compound **7h** with PI3Kα; (**b**) The 2D interaction model of compound **7h** with PI3Kα; (**c**) The 3D interaction model of compound **4** with PI3Kα; (**d**) The 2D interaction model of compound **4** with PI3Kα.

After that, we visualized and analyzed the above docking results. The 3D action models of the PI3Kα protein with compounds **7h** and **4**, respectively, are shown in Figure 9a,c. The diterpene parent nucleus and 6-fluoro-3-(piperidin-4-yl) benzo[*d*]isoxazole moiety of compound **7h** were embedded in the active pockets of the PI3Kα protein, respectively. Compared with compound **4**, compound **7h** showed a remarkable increase in the active pocket occupancy and binding stability of the PI3Kα protein. The 2D modelling of the interaction of the PI3Kα protein with compounds **7h** and **4**, respectively, is shown in Figure 9b,d. The 2D models of the interaction of the PI3Kα protein with compounds **7h** and **4**, respectively, are also shown in Figures S5 and S6 in the Supplementary Materials. Compound **7h** has the ability to bind to amino acid residues in the PI3Kα protein via hydrogen bonds (LYS271, ARG818, ASN756, GLN815, and GLN630), pi-cation (LYS271 and ARG818), pi-anion (GLU172), alkyl (PHE666), pi-alkyl (LYS271 and ARG818) and halogen (ASN822). In addition, the van der Waals force field, which consists of equal amino acid residues (PRO835, GLY837, MET811, ILE633, SER629, and ASN170), is also crucial for compound **7h** to be able to be immobilized in the active pocket of the PI3Kα protein.

Taken together, the introduction of a flexible side chain containing reactive groups at the C-6 position of the enmein-type diterpenoid **4** may enhance its PI3K inhibitory effect. Structural modifications that enhance the requisite capacity between the ligand molecule and its target protein may be a viable approach to improving the derivatives’ targeting abilities. The data and analysis of the above docking results provide critical clues for further structural modification or pharmacological research.

## 3. Conclusions

In conclusion, a novel 1,14-epoxy enmein-type diterpenoid and a library containing 21 novel enmein-type diterpenoid derivatives were constructed in this project. All the derivatives were evaluated for their anti-proliferative activities. Most derivatives displayed superior anti-proliferative activities against three cancer cell lines as compared with their parental compound **4**. Derivative **7h**, which was modified using succinic acid and an oxazole moiety, exhibited the most remarkable anti-proliferative activity against A549 cells, which was 11.03-fold higher than that of **4**. Additionally, **7h** exhibited a weak cytotoxicity against human normal L-02 cells and demonstrated a significant cancer cell-targeting property. The SARs analysis showed that the derivatives **6l**, **6m**, and **7a**–**h**, which introduced a flexible linker arm between the diterpenoid parental core and the active moiety, exhibited more significant anticancer activity than derivatives **4a**–**k**, which lacked the flexible linker arm. The biological studies revealed that **7h** affected A549 cells by up-regulating their intracellular ROS levels, down-regulating their MMP levels, and inducing cell cycle arrest and apoptosis in a dose-dependent manner. Further mechanism studies showed that **7h** exerted its anticancer effects through inhibition of the PI3K/Akt/mTOR pathway. Docking studies indicated that **7h** exhibited the best binding effect with PI3K, suggesting its potential as a novel PI3K inhibitor through further structural modification. In conclusion, the above studies provide some leads for the exploration of enmein-type diterpene derivatives as potential anticancer agents. Meanwhile, it is hoped that this work inspires more research teams to focus on the study of enmein-type diterpenoids in cancer therapy.

## 4. Materials and Methods

### 4.1. Chemistry

#### 4.1.1. Reagents and Apparatus

The starting material, Oridonin, was purchased from Chengdu Yirui Biotechnology Co., Ltd. (Chengdu, China), and the other reagents and solvents were purchased from Anegi (Shanghai, China) Medicinal Chemicals Co. All reagents were used directly after purchase and required no extra preparation. The chemical reactions were monitored using thin-layer chromatography, and the crude target products were purified using silica gel column chromatography. The silica gel preforms (model: HSGF254) and silica gel (100–200 mesh) were purchased from Yantai Yinlong Silica Gel Co (Yantai, China). The HRMS data were recorded on a Waters XEVO G2-XS QTOF high-resolution mass spectrometer. The NMR spectra were recorded on a Bruker AVANCE NEO 600 spectrometer, Fällanden, Switzerland, with CDCl_3_ or DMSO as solvent and tetramethylsilane (TMS) as internal standard. Coupling constants and chemical shift values are expressed in *J* (Hz) and d (ppm), respectively. The structures of all reported derivatives were consistent with the data from HRMS, ^1^H NMR, and ^13^C NMR spectra. The spectra of all derivatives are contained in Supplementary Materials.

#### 4.1.2. Synthesis of *Ent-6β,7β,14β-trihydroxy-1,15-dioxo-7,20-epoxy-16-kaurene* (**2**)

Oridonin (**1**, 1.00 g, 2.74 mmol) was dissolved in ice-cold acetone (100 mL) and then Jones reagent was dropped slowly into the system at 0 °C and stirred for 1 h. The reaction was monitored throughout the experiment via thin-layer chromatography (TLC). After the reaction, the solution was removed by spinning under reduced pressure, redissolved by dichloromethane (DCM, 100 mL), and then extracted sequentially with 5% sodium bicarbonate solution, distilled water, and saturated saline. The organic phase was separated and dried with anhydrous sodium sulfate. The organic solvent was removed by decompression evaporation, and the crude mixture was purified by flash column chromatography (DCM/MeOH, 25/1, *v*/*v*), resulting in the target intermediate compound **2** (0.90 g, Yield: 90%) as a white granule [30].

#### 4.1.3. Synthesis of *Ent-6β,14α-Dihydroxy-(1,7–1,14)-epoxy-7,15-dioxo-6,20-hemiketal-6,7-seco-16-kaurene* (**4**)

Compound **2** (0.36 g, 1.00 mmol) and anhydrous sodium carbonate (1.06 g, 10.00 mmol) were co-dissolved in anhydrous tetrahydrofuran (100 mL). The mixture was reacted at 25° C and the lead tetraacetate (2.00 g, 4.5 mmol) was added in batches (0.5 g/per/5 min). After the reaction, the insoluble impurities were filtered off, and the solution was removed by spinning under reduced pressure, redissolved by DCM (100 mL), and then extracted sequentially with 5% sodium bicarbonate solution, distilled water, and saturated saline. The organic phase was separated and dried with anhydrous sodium sulfate. The organic solvent was removed by decompression evaporation, and the crude mixture was purified by flash column chromatography (DCM/MeOH, 50/1, *v*/*v*), resulting in the target compound **4** (0.31 g, Yield: 85%) as a white granule. ^1^H NMR (600 MHz, CDCl_3_) δ values were as follows: 6.28 (1H, s, CH_2_-17), 5.54 (1H, s, CH_2_-17), 5.43 (1H, brs, H-6), 4.26 (1H, d, *J* = 5.7 Hz, H-14), 3.99 (1H, d, *J* = 9.7 Hz, CH_2_-20), 3.73 (1H, d, *J* = 9.7 Hz, CH_2_-20), 3.25 (1H, m, H-13), 2.53 (1H, m, CH_2_-12), 2.51 (1H, m, CH_2_-2), 2.16 (1H, m, H-9), 2.08 (2H, m, CH_2_-2, CH_2_-12), 1.99 (1H, d, *J* = 13.8 Hz, CH-3), 1.80 (1H, m, CH_2_-11), 1.60 (2H, m, CH_2_-3, H-5), 1.47 (1H, d, *J* = 13.8 Hz, CH_2_-11), 1.12 (3H, s, CH_3_-18), 1.01 (3H, s, CH_3_-19). ^13^C NMR (151 MHz, CDCl_3_) δ 194.53 (C15), 168.84 (C7), 146.52 (C16), 121.39 (C17), 107.43 (C1), 102.01 (C6), 79.23 (C14), 72.71 (C20), 54.43 (C8), 53.54 (C5), 53.35 (C10), 46.82 (C13), 39.63 (C9), 36.11 (C3), 33.29 (C4), 30.69 (C2), 27.76 (C12), 23.66 (C18), 23.37 (C19), and 19.63 (C11). HRMS (ESI) *m*/*z*: calculated for C_20_H_24_O_6_ [M + Na]^+^, 383.1471, found, 383.1468. The single crystal data for compound **4** is detailed in Supplemental Materials.

#### 4.1.4. General Procedure for the Synthesis of Target Derivatives **5a**–**m**

Compound **3** (1.00 mmol) and appropriate acyl chloride (1.50 mmol) were co-dissolved in anhydrous DCM (20 mL), DMAP (0.1 mmol) and triethylamine (1.50 mmol) were added as catalysts, and the mixture was stirred for 24 h at 25° C. After the reaction, the solution was removed by spinning under reduced pressure, redissolved by DCM (100 mL), and then extracted sequentially with 5% sodium bicarbonate solution, distilled water, and saturated saline. The organic solvent was removed by decompression evaporation, and the crude mixture was purified by flash column chromatography. The solid was precipitated from *n*-hexane to give the target derivatives **5a**–**m**.

**ent-6β,14α-Dihydroxy-(1,7–1,14)-epoxy-7,15-dioxo-6,20-hemiketal-6,7-seco-16-kaurene-6β-yl dimethylcarbamate (5a).** Yield: 85%, white granule. ^1^H NMR (600 MHz, Chloroform-*d*) values were as follows: δ 6.31 (1H, s, CH_2_-17), 6.23 (1H, s, CH_2_-17), 5.57 (1H, brs, H-6), 4.29 (1H, d, *J* = 5.7 Hz, H-14), 4.04 (1H, d, *J* = 9.9 Hz, CH_2_-20), 3.77 (1H, d, *J* = 9.9 Hz, CH_2_-20), 3.29 (1H, m, H-13), 2.91 (3H, s, CH_3_-1′), 2.78 (3H, s, CH_3_-2′), 2.51 (1H, d, *J* = 5.6 Hz, H-9), 2.23 (1H, s), 2.18 (1H, d, *J* = 9.8 Hz), 2.10 (2H, m), 2.02 (1H, d, *J* = 14.0 Hz), 1.81 (1H, m), 1.67 (1H, m), 1.63 (1H, m), 1.51 (1H, d, *J* = 13.8 Hz), 1.19 (3H, s, CH_3_-18), 1.08 (3H, s, CH_3_-19). ^13^C NMR (151 MHz, CDCl_3_) δ 194.13 (C15), 168.25 (C7), 155.12 (C1′), 146.23 (C16), 121.82 (C17), 107.23 (C1), 103.22 (C6), 79.70 (C14), 72.71 (C20), 54.10 (C8), 53.24 (C5), 52.76 (C10), 46.21 (C13), 39.39 (C9), 36.64 (C1′), 36.09 (C3), 35.93 (C2′), 33.18 (C4), 31.15 (C2), 27.68 (C12), 23.65 (C18), 23.30 (C19), and 19.71 (C11). HRMS (ESI) *m*/*z*: calculated for C_23_H_29_NO_7_ [M + Na]^+^, 454.1842, found, 454.1847.

**ent-6β,14α-Dihydroxy-(1,7–1,14)-epoxy-7,15-dioxo-6,20-hemiketal-6,7-seco-16-kaurene-6β-yl diethylcarbamate (5b).** Yield: 82%, white granule. ^1^H NMR (600 MHz, Chloroform-*d*) values were as follows: δ 6.30 (1H, s, CH_2_-17), 6.23 (1H, s, CH_2_-17), 5.57 (1H, brs, H-6), 4.28 (1H, d, *J* = 5.7 Hz, H-14), 4.03 (1H, d, *J* = 9.9 Hz, CH_2_-20), 3.75 (1H, d, *J* = 9.9 Hz, CH_2_-20), 3.28 (2H, m, CH_2_-2′), 3.25 (1H, m, H-13) 3.13 (1H, m, H-3′), 3.07 (1H, m, H-3′), 2.48 (1H, d, *J* = 5.4 Hz, H-9), 2.24 (1H, s), 2.17 (1H, d, *J* = 9.8 Hz), 2.10 (2H, m), 2.01 (1H, d, *J* = 14.0 Hz), 1.81 (1H, m), 1.60 (2H, m), 1.50 (1H, d, *J* = 13.8 Hz), 1.18 (3H, s, CH_3_-18), 1.11 (3H, m, CH_3_-4′), 1.07 (3H, s, CH_3_-19), 1.03 (3H, m, CH_3_-5′). ^13^C NMR (150 MHz, CDCl_3_) δ: 194.14 (C15), 168.26 (C7), 154.53 (C1′), 146.19 (C16), 121.86 (C17), 107.27 (C1), 103.09 (C6), 79.67 (C14), 72.56 (C20), 54.14 (C8), 53.24 (C5), 52.69 (C10), 46.27 (C13), 41.91 (C2′), 41.06 (C3′), 39.41 (C9), 36.13 (C3), 33.21 (C4), 31.21 (C2), 27.69 (C12), 23.67 (C18), 23.29 (C19), 19.61 (C11), 14.24 (C4′), and 13.41 (C5′). HRMS (ESI) *m*/*z*: calculated for C_25_H_33_NO_7_ [M + Na]^+^, 482.2155, found, 482.2154.

**ent-6β,14α-Dihydroxy-(1,7–1,14)-epoxy-7,15-dioxo-6,20-hemiketal-6,7-seco-16-kaurene-6β-yl ethyl(methyl)carbamate (5c).** Yield: 86%, white granule. ^1^H NMR (600 MHz, Chloroform-*d*) values were as follows: δ 6.31 (1H, s, CH_2_-17), 6.23 (1H, s, CH_2_-17), 5.58 (1H, brs, H-6), 4.29 (1H, d, *J* = 5.7 Hz, H-14), 4.04 (1H, d, *J* = 9.9 Hz, CH_2_-20), 3.77 (1H, d, *J* = 9.9 Hz, CH_2_-20), 3.29 (2H, m, CH_2_-2′), 3.14 (1H, d, *J* = 46.9 Hz, H-13), 2.81 (3H, d, *J* = 79.5 Hz, CH_3_-3′), 2.50 (1H, d, *J* = 13.2 Hz, H-9), 2.24 (1H, s), 2.18 (1H, m), 2.10 (2H, m), 2.02 (1H, d, *J* = 13.9 Hz), 1.81 (1H, m), 1.66 (1H, m), 1.63 (1H, m), 1.51 (1H, d, *J* = 13.9 Hz), 1.19 (3H, s, CH_3_-18), 1.11-1.03 (6H, m, CH_3_-19, CH_3_-3′). ^13^C NMR (151 MHz, CDCl_3_) δ 194.02 (C15), 168.14 (C7), 154.57 (C1′), 146.1 (C16), 121.69 (C17), 107.14 (C1), 102.98 (C6), 79.58 (C14), 72.47 (C20), 54.01 (C8), 53.14 (C5), 52.62 (C10), 46.13 (C13), 43.89 (C2′), 39.29 (C9), 36.00 (C3), 33.08 (C4), 31.04 (C2), 29.70 (C3′), 27.58 (C12), 23.55 (C18), 23.20 (C19), 19.60 (C11) and 12.43 (C4′). HRMS (ESI) *m*/*z*: calculated for C_24_H_31_NO_7_ [M + Na]^+^, 468.1998, found, 468.1993.

**ent-6β,14α-Dihydroxy-(1,7–1,14)-epoxy-7,15-dioxo-6,20-hemiketal-6,7-seco-16-kaurene-6β-yl bis(2-chloroethyl) carbamate (5d).** Yield: 80%, white granule. ^1^H NMR (600 MHz, CDCl_3_) values were as follows: δ 6.31 (1H, s, CH_2_-17), 6.20 (1H, s, CH_2_-17), 5.58 (1H, brs, H-6), 4.28 (1H, d, *J* = 5.7 Hz, H-14), 4.04 (1H, d, *J* = 9.9 Hz, CH_2_-20), 3.76 (1H, d, *J* = 9.9 Hz, CH_2_-20), 3.70 (2H, m, CH_2_-2′), 3.65 (2H, m, CH_2_-2′), 3.56 (2H, m, CH_2_-2′), 3.49 (2H, m, CH_2_-2′), 3.28 (1H, m, H-13), 2.45 (1H, m, H-9), 2.26 (1H, s), 2.10 (2H, m), 2.07 (1H, m), 2.08 (1H, d, *J* = 13.8 Hz), 2.02 (1H, d, *J* = 13.9 Hz), 1.82 (1H, m), 1.73 (1H, m), 1.63 (1H, m), 1.51 (1H, d, *J* = 14.0 Hz), 1.18 (3H, s, CH_3_-18), 1.07 (3H, s, CH_3_-19). ^13^C NMR (151 MHz, CDCl_3_) δ 193.80 (C15), 168.10 (C7), 154.65 (C1′), 146.01 (C16), 122.05 (C17), 107.03 (C1), 103.96 (C6), 79.88 (C14), 72.55 (C20), 54.02 (C8), 53.10 (C5), 52.71 (C10), 50.85 (C2′), 50.11 (C3′), 46.02 (C13), 41.85 (C4′), 41.32 (C5′), 39.32 (C9), 36.04 (C3), 33.21 (C4), 31.25 (C2), 27.65 (C12), 23.63 (C18), 23.18 (C19), and 19.68 (C11). HRMS (ESI) *m*/*z*: calculated for C_25_H_31_Cl_2_NO_7_ [M + Na]^+^, 550.1376, found, 550.1375.

**ent-6β,14α-Dihydroxy-(1,7–1,14)-epoxy-7,15-dioxo-6,20-hemiketal-6,7-seco-16-kaurene-6β-yl diallylcarbamate (5e).** Yield: 85%, white granule. ^1^H NMR (600 MHz, DMSO) values were as follows: δ 6.13 (2H, s, CH_2_-17), 5.74 (1H, m, CH-4′), 5.69 (1H, brs, H-6), 5.66 (1H, m, CH-5′), 5.12 (2H, d, *J* = 10.7 Hz, CH_2_-7′), 5.07 (1H, d, *J* = 10.4 Hz, CH_2_-6′), 5.01 (1H, d, *J* = 10.4 Hz, CH_2_-6′), 4.41 (1H, d, *J* = 4.5 Hz, H-14), 4.04 (1H, d, *J* = 9.6 Hz, CH_2_-20), 3.81 (1H, m, CH_2_-3′), 3.73 (2H, d, *J* = 15.7 Hz, CH2-2′), 3.64 (1H, m, CH_2_-3′), 3.51 (1H, d, *J* = 9.6 Hz, CH_2_-20), 3.30 (1H, m, H-13), 2.32 (1H, d, *J* = 5.6 Hz), 2.19 (1H, s), 2.16 (1H, d, *J* = 10.2 Hz), 2.06 (1H, d, *J* = 10.2 Hz), 1.99 (1H, m), 1.86 (1H, d, *J* = 13.8 Hz), 1.70 (1H, m), 1.52 (1H, m), 1.46 (2H, d, *J* = 13.8 Hz), 1.23 (1H, s), 1.09 (3H, s, CH_3_-18), 0.99 (3H, s, CH_3_-19). ^13^C NMR (151 MHz, DMSO) δ 193.72 (C15), 167.64 (C7), 154.06 (C1′) 146.30 (C16), 133.64 (C4′), 133.38 (C5′), 121.31 (C17), 117.16 (C6′), 115.79 (C7′), 106.62 (C1), 102.26 (C6), 78.84 (C14), 71.41 (C20), 53.29 (C8), 52.49 (C5), 51.78 (C10), 48.85 (C2′), 47.76 (C3′), 45.47 (C13), 38.27 (C9), 35.36 (C3), 32.59 (C4), 30.69 (C2), 27.03 (C12), 23.22 (C18), 22.62 (C19), and 19.30 (C11). HRMS (ESI) *m*/*z*: calculated for C_27_H_33_NO_7_ [M + Na]^+^, 506.2155, found, 506.2152.

**ent-6β,14α-Dihydroxy-(1,7–1,14)-epoxy-7,15-dioxo-6,20-hemiketal-6,7-seco-16-kaurene-6β-yl pyrrolidine-1-carboxylate (5f).** Yield: 80%, white granule. ^1^H NMR (600 MHz, DMSO) values were as follows: δ 6.12 (2H, d, *J* = 6.0 Hz, CH_2_-17), 5.70 (1H, brs, H-6), 4.41 (1H, d, *J* = 4.5 Hz, H-14), 4.02 (1H, d, *J* = 9.6 Hz, CH_2_-20), 3.53 (1H, d, *J* = 9.6 Hz, CH_2_-20), 3.30 (1H, m, H-13), 3.22 (2H, m, CH_2_-2′), 3.15 (2H, m, CH_2_-5′), 2.35 (1H, d, *J* = 5.4 Hz, H-9), 2.23 (1H, d, *J* = 10.1 Hz), 2.17 (2H, m), 1.99 (1H, m), 1.86 (1H, d, *J* = 13.9 Hz), 1.77 (2H, m, CH_2_-3′), 1.74 (2H, m, CH_2_-4′), 1.70 (1H, m), 1.55 (2H, m), 1.46 (1H, d, *J* = 13.7 Hz), 1.08 (3H, s, CH_3_-18), 0.99 (3H, s, CH_3_-19). ^13^C NMR (151 MHz, DMSO) δ 193.81 (C15), 167.69 (C7), 152.56 (C1′) 146.36 (C16), 121.28 (C17), 106.65 (C1), 101.13 (C6), 78.71 (C14), 71.48 (C20), 53.41 (C8), 52.57 (C5), 51.85 (C10), 46.11 (C13), 45.69 (C2′), 45.23 (C5′), 38.30 (C9), 35.34 (C3), 32.67 (C4), 30.64 (C2), 27.08 (C12), 25.25 (C3′), 24.26 (C4′), 23.25 (C18), 22.70 (C19), and 19.42 (C11). HRMS (ESI) *m*/*z*: calculated for C_25_H_31_NO_7_ [M + Na]^+^, 480.1998, found, 480.2003.

**ent-6β,14α-Dihydroxy-(1,7–1,14)-epoxy-7,15-dioxo-6,20-hemiketal-6,7-seco-16-kaurene-6β-yl piperidine-1-carboxylate (5g).** Yield: 76%, white granule. ^1^H NMR (600 MHz, DMSO) values were as follows: δ 6.14 (2H, d, *J* = 4.5 Hz, CH_2_-17), 5.70 (1H, brs, H-6), 4.41 (1H, d, *J* = 4.4 Hz, H-14), 4.04 (1H, d, *J* = 9.6 Hz, CH_2_-20), 3.56 (1H, d, *J* = 9.6 Hz, CH_2_-20), 3.31 (1H, m, H-13), 3.30-3.14 (4H, m, CH_2_-2′, CH_2_-6′) 2.34 (1H, d, *J* = 5.6 Hz, H-9), 2.16 (2H, m), 2.02 (2H, m), 1.86 (1H, d, *J* = 13.9 Hz), 1.70 (1H, m), 1.55 (1H, d, *J* = 10.4 Hz), 1.50 (2H, m, CH_2_-4′), 1.47 (1H, m), 1.44 (1H, m), 1.42 (2H, m, CH_2_-3′), 1.37 (2H, m, CH_2_-5′), 1.10 (3H, s, CH_3_-18), 0.99 (3H, s, CH_3_-19). ^13^C NMR (151 MHz, DMSO) δ 194.25 (C15), 168.08 (C7), 153.36 (C1′), 146.73 (C16), 121.86 (C17), 107.11 (C1), 102.74 (C6), 79.29 (C14), 71.88 (C20), 53.74 (C8), 52.97 (C5), 52.56 (C10), 46.03 (C13), 44.51 (C2′, C6′), 38.71 (C9), 35.82 (C3), 33.05 (C4), 31.14 (C2), 27.52 (C12), 25.52 (C3′, C5′), 34.12 (C4′), 23.68 (C18), 23.11 (C19), and 19.70 (C11). HRMS (ESI) *m*/*z*: calculated for C_26_H_33_NO_7_ [M + Na]^+^, 494.2155, found, 494.2151.

**ent-6β,14α-Dihydroxy-(1,7–1,14)-epoxy-7,15-dioxo-6,20-hemiketal-6,7-seco-16-kaurene-6β-yl azepane-1-carboxylate (5h).** Yield: 72%, white granule. ^1^H NMR (600 MHz, DMSO) values were as follows: δ 6.28 (2H, d, *J* = 3.9 Hz, CH_2_-17), 5.70 (1H, brs, H-6), 4.42 (1H, d, *J* = 4.5 Hz, H-14), 4.04 (1H, d, *J* = 9.6 Hz, CH_2_-20), 3.53 (1H, d, *J* = 9.6 Hz, CH_2_-20), 3.39 (1H, m, CH_2_-2′), 3.30 (1H, m, H-13), 3.26 (1H, m, CH_2_-3′), 3.23 (1H, m, CH_2_-2′), 3.13 (1H, m, CH_2_-3′), 2.31 (1H, d, *J* = 5.6 Hz, H-9), 2.19 (2H, m), 2.14 (1H, m), 2.01 (1H, m), 1.86 (1H, d, *J* = 13.8 Hz), 1.71 (1H, m), 1.59 (2H, d, *J* = 5.4 Hz), 1.56 (1H, m), 1.48 (4H, m, CH_2_-3′, CH_2_-6′), 1.44 (4H, m, CH_2_-4′, CH_2_-5′), 1.23 (1H, s), 1.09 (3H, s, CH_3_-18), 1.00 (3H, s, CH_3_-19). ^13^C NMR (151 MHz, DMSO) δ 193.84 (C15), 167.62 (C7), 154.03 (C1′), 146.28 (C16), 121.41 (C17), 106.66 (C1), 101.87 (C6), 78.79 (C14), 71.41 (C20), 53.37 (C8), 52.52 (C5), 51.85 (C10), 46.38 (C13), 45.70 (C2′), 45.65 (C7′), 38.26 (C9), 35.36 (C3), 32.61 (C4), 30.71 (C2), 28.20 (C12), 27.41 (C3′), 27.65 (C6′), 26.75 (C4′), 26.32 (C5′), 23.24 (C18), 23.65 (C19), and 19.27 (C11). HRMS (ESI) *m*/*z*: calculated for C_27_H_35_NO_7_ [M + Na]^+^, 508.2311, found, 508.2311.

**ent-6β,14α-Dihydroxy-(1,7–1,14)-epoxy-7,15-dioxo-6,20-hemiketal-6,7-seco-16-kaurene-6β-yl bmorpholine-4-carboxylate (5i).** Yield: 65%, white granule. ^1^H NMR (600 MHz, CDCl_3_) values were as follows: δ 6.32 (1H, s, CH_2_-17), 6.27 (1H, s, CH_2_-17), 5.59 (1H, brs, H-6), 4.29 (1H, d, *J* = 4.5 Hz, H-14), 4.05 (1H, d, *J* = 9.9 Hz, CH_2_-20), 3.77 (1H, d, *J* = 9.9 Hz, CH_2_-20), 3.66 (2H, s, CH_2_-2′), 3.59 (2H, s, CH_2_-3′), 3.51 (1H, m, CH_2_-4′), 3.39 (1H, m, CH_2_-4′), 3.35 (1H, m, CH_2_-5′), 3.29 (1H, m, H-13), 3.24 (1H, m, CH_2_-4′), 2.46 (1H, d, *J* = 5.2 Hz, H-9), 2.24 (1H, s), 2.10 (2H, m), 2.04 (2H, m), 1.82 (1H, m), 1.65 (1H, m), 1.62 (2H, m), 1.52 (1H, d, *J* = 13.9 Hz), 1.20 (3H, s, CH_3_-18), 1.08 (3H, s, CH_3_-19). ^13^C NMR (151 MHz, CDCl_3_) δ 193.95 (C15), 168.02 (C7), 153.78 (C1′), 145.95 (C16), 121.95 (C17), 107.03 (C1), 103.64 (C6), 79.71 (C14), 72.43 (C20), 66.55 (C2′), 66.27 (C3′), 53.91 (C8), 53.05 (C5), 52.82 (C10), 46.00 (C13), 44.07 (C4′), 43.78 (C5′), 39.23 (C9), 35.95 (C3), 33.06 (C4), 31.08 (C2), 27.56 (C12), 23.52 (C18), 23.15 (C19), and 19.56 (C11). HRMS (ESI) *m*/*z*: calculated for C_25_H_31_NO_8_ [M + Na]^+^, 496.1947, found, 496.1942.

**ent-6β,14α-Dihydroxy-(1,7–1,14)-epoxy-7,15-dioxo-6,20-hemiketal-6,7-seco-16-kaurene-6β-yl diphenylcarbamate (5j).** Yield: 72%, white granule. ^1^H NMR (600 MHz, DMSO) values were as follows: δ 7.31 (4H, t, *J* = 7.7 Hz, H-Ar), 7.26-7.24 (4H, m, H-Ar), 7.19 (2H, t, *J* = 7.3 Hz, H-Ar), 6.22 (1H, s, CH_2_-17), 6.09 (1H, s, CH_2_-17), 5.59 (1H, brs, H-6), 4.29 (1H, d, *J* = 5.7 Hz, H-14), 4.04 (1H, d, *J* = 9.6 Hz, CH_2_-20), 3.53 (1H, d, *J* = 9.5 Hz, CH_2_-20), 3.14 (1H, m, H-13), 2.13 (1H, m, H-9), 2.01 (1H, s), 1.80 (2H, m), 1.72 (1H, m), 1.63 (1H, m), 1.43 (1H, d, *J* = 13.7 Hz), 1.27 (1H, m), 1.24 (1H, m), 1.23 (1H, m), 1.08 (3H, s, CH_3_-18), 0.98 (3H, s, CH_3_-19). ^13^C NMR (151 MHz, DMSO) δ 193.24 (C15), 167.66 (C7), 153.10 (C1′), 146.54 (C16), 129.14 (Ar), 127.08 (Ar), 120.56 (C17), 106.44 (C1), 102.00 (C6), 79.01 (C14), 71.31 (C20), 53.04 (C8), 52.56 (C5), 51.48 (C10), 45.49 (C13), 38.17 (C9), 35.28 (C3), 32.63 (C4), 30.98 (C2), 26.93 (C12), 23.22 (C18), 22.08 (C19), and 16.93 (C11). HRMS (ESI) *m*/*z*: calculated for C_33_H_33_NO_7_ [M + Na]^+^, 578.2155, found, 578.2150.

**ent-6β,14α-Dihydroxy-(1,7–1,14)-epoxy-7,15-dioxo-6,20-hemiketal-6,7-seco-16-kaurene-6β-yl 10H-phenothiazine-10-carboxylate (5k).** Yield: 88%, yellow granule. ^1^H NMR (600 MHz, DMSO) values were as follows: δ 7.32 (2H, s, H-Ar), 7.23 (2H, d, *J* = 7.8 Hz, H-Ar), 7.09 (2H, s, H-Ar), 6.99 (2H, s, H-Ar), 6.09 (1H, s, CH_2_-17), 5.85 (1H, s, CH_2_-17), 5.37 (1H, brs, H-6), 4.07 (1H, d, *J* = 5.7 Hz, H-14), 3.87 (1H, d, *J* = 9.6 Hz, 1H, CH_2_-20), 3.39 (1H, d, *J* = 9.6 Hz, CH_2_-20), 2.94 (1H, m, H-13), 1.92 (1H, s, H-9), 1.80 (1H, s), 1.64 (1H, s), 1.58 (1H, d, *J* = 13.9 Hz), 1.53 (1H, d, *J* = 15.1 Hz), 1.39 (1H, m), 1.19 (1H, d, *J* = 13.9 Hz), 0.99 (3H, s), 0.83 (3H, s, CH_3_-18), 0.76 (3H, s, CH_3_-19). ^13^C NMR (151 MHz, DMSO) δ 193.26 (C15), 167.59 (C7), 151.35 (C1′), 146.41 (C16), 137.40 (Ar), 131.52 (Ar×2), 129.67 (Ar), 127.48 (Ar×2), 127.36 (Ar×2), 127.13 (Ar×2), 126.89 (Ar×2), 120.78 (C17), 106.42 (C1), 103.37 (C6), 79.33 (C14), 71.32 (C20), 52.96 (C8), 52.26 (C5), 52.05 (C10), 45.31 (C13), 38.16 (C9), 35.19 (C3), 32.48 (C4), 30.56 (C2), 26.94 (C12), 23.15 (C18), 22.60 (C19), and 17.55 (C11). HRMS (ESI) *m*/*z*: calculated for C_33_H_31_NO_7_ [M + Na]^+^, 608.1719, found, 608.1718.

**ent-6β,14α-Dihydroxy-(1,7–1,14)-epoxy-7,15-dioxo-6,20-hemiketal-6,7-seco-16-kaurene-6β-yl 3-(1H-pyrrol-1-yl) propanoate (5l).** Yield: 85%, white granule. ^1^H NMR (600 MHz, DMSO-d_6_) values were as follows: δ 6.66 (2H, s, H-Ar), 6.12 (1H, s, H-Ar), 6.09 (1H, s, H-Ar), 5.93 (2H, s, CH_2_-17), 5.68 (1H, brs, H-6), 4.39 (1H, d, *J* = 5.7 Hz), 4.07 (2H, t, *J* = 6.6 Hz, CH_2_-3′), 4.04 (1H, d, *J* = 9.6 Hz), 3.48 (1H, d, *J* = 9.6 Hz), 3.27 (1H, m), 2.72 (1H, m, CH_2_-2′), 2.67 (1H, m, CH_2_-2′), 2.27 (1H, d, *J* = 5.3 Hz), 2.15 (1H, m), 2.08 (1H, s), 1.95 (1H, d, *J* = 15.7 Hz), 1.90 (1H, m), 1.84 (1H, d, *J* = 13.8 Hz), 1.67 (1H, m), 1.49 (1H, m), 1.46 (1H, m), 1.44 (1H, m), 1.07 (3H, s, CH_3_-18), 0.98 (3H, s, CH_3_-19). ^13^C NMR (151 MHz, DMSO) δ 193.68 (C15), 170.10 (C1′), 167.72 (C7), 146.48 (C16), 121.03 (C17), 120.51 (Ar), 107.69 (Ar), 106.52 (C1), 100.67 (C6), 78.94 (C14), 71.49 (C20), 53.22 (C8), 52.54 (C5), 51.79 (C10), 45.44 (C13), 44.32 (C2′), 38.32 (C9), 36.46 (C3′), 35.34 (C3), 32.63 (C4), 30.50 (C2), 27.00 (C12), 23.07 (C18), 22.78 (C19), and 18.81 (C11). HRMS (ESI) *m*/*z*: calculated for C_27_H_31_NO_7_ [M + Na]^+^, 504.1998, found, 504.1997.

**ent-6β,14α-Dihydroxy-(1,7–1,14)-epoxy-7,15-dioxo-6,20-hemiketal-6,7-seco-16-kaurene-6β-yl 3-(1,3-dioxoisoindolin-2-yl) propanoate (5m).** Yield: 90%, white granule. ^1^H NMR (600 MHz, DMSO) values were as follows: δ 7.87 (2H, d, *J* = 5.3 Hz, H-Ar), 7.84 (2H, d, *J* = 5.2 Hz, H-Ar), 6.13 (1H, s, CH_2_-17), 6.03 (1H, s, CH_2_-17), 5.68 (1H, brs, H-6), 4.37 (1H, d, *J* = 5.7 Hz, H-14), 3.93 (1H, d, *J* = 9.6 Hz, 1H, CH_2_-20), 3.82 (1H, m, CH_2_-2′), 3.75 (1H, m, CH_2_-2′), 3.26 (1H, m, H-9), 3.16 (1H, d, *J* = 9.6 Hz, CH_2_-20), 2.66 (1H, m, CH_2_-3′), 2.61 (1H, m, CH_2_-3′), 2.24 (1H, s), 2.10 (2H, m), 2.07 (1H, m), 1.86 (1H, m), 1.81 (1H, d, *J* = 14.0 Hz), 1.64 (1H, m), 1.49 (1H, m), 1.42 (2H, m), 1.03 (3H, s, CH_3_-18), 0.94 (3H, s, CH_3_-19). ^13^C NMR (151 MHz, DMSO) δ 193.67 (C15), 169.54 (C4′, C7′), 167.59 (C7, C1′), 146.41 (C16), 134.50 (Ar×2), 131.49 (Ar×2), 123.14 (Ar×2), 121.06 (C17), 106.42 (C1), 100.40 (C6), 78.74 (C14), 71.45 (C20), 53.13 (C8), 52.46 (C5), 51.67 (C10), 45.35 (C13), 38.26 (C9), 35.30 (C3), 33.50 (C4), 32.80 (C2′), 32.53 (C3′), 30.42 (C2), 26.93 (C12), 23.01 (C18), 22.64 (C19), and 18.85 (C11). HRMS (ESI) *m*/*z*: calculated for C_31_H_31_NO_9_ [M + Na]^+^, 584.1897, found, 584.1901.

#### 4.1.5. Synthesis of *4-(Ent-6β,14α-dihydroxy-(1,7–1,14)-epoxy-7,15-dioxo-6,20-hemi-ketal-6,7-seco-16-kaurene-6β-yloxy)-4-oxobutanoic Acid* (**6**)

Compound **4** (0.36 g, 1.00 mmol), succinic anhydride (0.15 g, 1.5 mmol), and DMAP (15mg, 0.12 mmol) were co-dissolved in anhydrous DCM (50 mL) and stirred for 4 h at 25 °C. After the reaction, the solution was removed by spinning under reduced pressure, redissolved by DCM (100 mL), and then extracted sequentially with 5% sodium bicarbonate solution, distilled water, and saturated saline. The organic solvent was removed by decompression evaporation, and the crude mixture was purified by flash column chromatography (DCM/MeOH, 50/3, *v*/*v*), resulting in the target intermediate compound **6** (0.37g, Yield: 80%) as a white granule. ^1^H NMR (600 MHz, DMSO) values were as follows: δ 12.25 (1H, s, OH-4′), 6.13 (1H, s, CH_2_-17), 6.11 (1H, s, CH_2_-17), 5.70 (1H, brs, H-6), 4.42 (1H, d, *J* = 5.7 Hz, H-14), 4.07 (1H, d, *J* = 9.6 Hz, CH_2_-20), 3.53 (1H, d, *J* = 9.6 Hz, CH_2_-20), 3.30 (1H, m, H-13), 2.47 (1H, m, H-9), 2.45 (1H, d, *J* = 5.4 Hz, CH_2_-3′), 2.42 (2H, d, *J* = 1.9 Hz, CH_2_-2′), 2.36 (1H, d, *J* = 5.1 Hz, CH_2_-3′), 2.16 (3H, m), 1.98 (1H, m), 1.86 (1H, d, *J* = 13.9 Hz), 1.70 (1H, m), 1.54 (2H, m), 1.46 (1H, d, *J* = 13.8 Hz), 1.23 (1H, s), 1.08 (3H, s, CH_3_-18), 0.99 (3H, s, CH_3_-19). ^13^C NMR (151 MHz, DMSO) δ 193.77 (C15), 173.35 (C4′), 171.14 (C1′), 167.73 (C7), 146.47 (C16), 121.13 (C17), 106.59 (C1), 100.84 (C6), 78.96 (C14), 71.50 (C20), 53.38 (C8), 52.56 (C5), 51.97 (C10), 45.67 (C13), 38.33 (C9), 35.37 (C3), 32.60 (C4), 30.56 (C2), 29.03 (C2′), 28.37 (C3′), 27.03 (C12), 23.11 (C18), 22.77 (C19), and 19.03 (C11). HRMS (ESI) *m*/*z*: calculated for C_24_H_28_O_9_ [M + Na]^+^, 483.1631, found, 483.1633.

#### 4.1.6. General Procedure for the Synthesis of Target Derivatives **7a**–**h**

Compound **6** (1.00 mmol), PyBOP (1.5 mmol), and triethylamine (1.50 mmol) were co-dissolved in anhydrous DCM (20 mL), activated for 1 h at 25 °C, and then amine (1.50 mmol) was added and stirred for 2 h. After the reaction, the solution was removed by spinning under reduced pressure, redissolved by DCM (100 mL), and then extracted sequentially with 5% sodium bicarbonate solution, distilled water, and saturated saline. The organic solvent was removed by decompression evaporation, and the crude mixture was purified by flash column chromatography. The solid was precipitated from *n*-hexane to give the target derivatives **7a**–**h**.

**ent-6β,14α-Dihydroxy-(1,7–1,14)-epoxy-7,15-dioxo-6,20-hemiketal-6,7-seco-16-kaurene-6β-yl 4-(benzylamino)-4-oxobutanoate (7a).** Yield: 75%, white granule. ^1^H NMR (600 MHz, DMSO) values were as follows: δ 8.37 (1H, s, NH), 7.30 (2H, t, *J* = 7.6 Hz, H-Ar), 7.24 (2H, t, *J* = 14.2 Hz, H-Ar), 7.22 (1H, s, H-Ar), 6.12 (2H, *J* = 14.1 Hz, CH_2_-17), 5.70 (1H, brs, H-6), 4.42 (1H, d, *J* = 4.4 Hz, H-14), 4.24 (2H, d, *J* = 3.4 Hz, CH_2_-5′), 4.07 (1H, d, *J* = 9.6 Hz, CH_2_-20), 3.52 (1H, d, *J* = 9.6 Hz, CH_2_-20), 3.30 (1H, s, H-13), 2.48 (d, *J* = 6.5 Hz, CH_2_-2′), 2.39 (2H, t, *J* = 6.4 Hz, CH_2_-3′), 2.35 (1H, d, *J* = 6.5 Hz, CH_2_-2′), 2.16 (2H, s), 1.98 (2H, d, *J* = 5.8 Hz), 1.86 (1H, d, *J* = 6.8 Hz), 1.70 (1H, m), 1.53 (2H, d, *J* = 9.2 Hz), 1.46 (1H, d, *J* = 13.8 Hz), 1.23 (2H, s), 1.08 (3H, s, CH_3_-18), 1.00 (3H, s, CH_3_-19). ^13^C NMR (150 MHz, DMSO) δ: 193.80 (C15), 171.38 (C4′), 170.57 (C1′), 167.74 (C7), 146.47 (C16), 139.51 (Ar), 128.26 (Ar), 127.18 (Ar), 126.74 (Ar), 121.13 (C17), 106.60 (C1), 100.77 (C6), 78.93 (C14), 71.50 (C20), 53.29 (C8), 52.57 (C5), 51.94 (C10), 45.71 (C13), 42.04 (C5′), 38.33 (C9), 35.37 (C3), 32.60 (C4), 30.56 (C2), 29.43 (C2′), 29.28 (C3′), 27.04 (C12), 23.12 (C18), 22.76 (C19), and 19.03 (C11). HRMS (ESI) *m*/*z*: calculated for C_31_H_35_NO_8_ [M + Na]^+^, 572.2260, found, 572.2259.

**ent-6β,14α-Dihydroxy-(1,7–1,14)-epoxy-7,15-dioxo-6,20-hemiketal-6,7-seco-16-kaurene-6β-yl 4-((4-methylbenzyl) amino)-4-oxobutanoate (7b)**. Yield: 72%, white granule. ^1^H NMR (600 MHz, DMSO-d_6_) values were as follows: δ 8.30 (1H, s, NH), 7.11 (4H, s, Ar), 6.14 (1H, s, CH_2_-17), 6.11 (1H, s, CH_2_-17), 5.70 (1H, brs, H-6), 4.41 (1H, d, *J* = 5.8 Hz, H-14), 4.19 (2H, s, CH_2_-5′), 4.06 (1H, d, *J* = 9.3 Hz, CH_2_-20), 3.52 (1H, d, *J* = 9.9 Hz, CH_2_-20), 3.30 (1H, m), 2.48 (1H, m, CH_2_-2′), 2.37 (1H, m, CH_2_-2′), 2.36 (2H, m, CH_2_-3′), 2.27 (3H, m, CH_3_-6′), 2.16 (2H, m), 1.98 (1H, m), 1.86 (1H, d, *J* = 13.6 Hz), 1.70 (1H, m), 1.54 (2H, d, *J* = 13.3 Hz), 1.46 (1H, d, *J* = 13.5 Hz), 1.24 (2H, s), 1.08 (3H, s, CH_3_-18), 1.00 (3H, s, CH_3_-19). ^13^C NMR (150 MHz, DMSO) δ: 193.77 (C15), 171.35 (C4′), 170.46 (C1′), 167.72 (C7), 146.46 (C16), 136.45 (Ar), 135.74 (Ar), 128.78 (Ar), 127.18 (Ar), 121.09 (C17), 106.59 (C1), 100.74 (C6), 78.92 (C14), 71.51 (C20), 53.29 (C8), 52.57 (C5), 51.95 (C10), 45.71 (C13), 41.80 (C5′), 38.34 (C9), 35.36 (C3), 32.58 (C4), 30.56 (C2), 29.44 (C2′), 29.30 (C3′), 27.03 (C12), 23.10 (C18), 22.75 (C19), 20.65 (C6′), and 19.01 (C11). HRMS (ESI) *m*/*z*: calculated for C_32_H_37_NO_8_ [M + Na]^+^, 586.2417, found, 586.2420.

**ent-6β,14α-Dihydroxy-(1,7–1,14)-epoxy-7,15-dioxo-6,20-hemiketal-6,7-seco-16-kaurene-6β-yl 4-((4-meth0xybenzyl) amino)-4-oxobutanoate (7c)**. Yield: 80%, white granule. ^1^H NMR (600 MHz, DMSO) values were as follows: δ 8.28 (1H, s, NH), 7.15 (2H, d, *J* = 8.2 Hz, H-Ar), 6.86 (2H, d, *J* = 8.5 Hz, H-Ar), 6.12 (2H, *J* = 16.1 Hz, CH_2_-17), 5.70 (1H, brs, H-6), 4.41 (1H, d, *J* = 5.7 Hz, H-14), 4.16 (2H, d, *J* = 4.0 Hz, CH_2_-5′), 4.07 (1H, d, *J* = 9.5 Hz, CH_2_-20), 3.72 (3H, s, OCH_3_-6′), 3.52 (1H, d, *J* = 9.6 Hz, CH_2_-20), 3.30 (1H, m, H-13), 2.48 (2H, d, *J* = 6.1 Hz, CH_2_-2′), 2.35 (2H, d, *J* = 6.2 Hz, CH_2_-3′), 2.18(2H, m), 1.98 (1H, m), 1.86 (1H, d, *J* = 13.8 Hz), 1.70 (1H, m), 1.54 (2H, d, *J* = 9.8 Hz), 1.46 (1H, d, *J* = 13.7 Hz), 1.23 (2H, s), 1.08 (3H, s, CH_3_-18), 0.99 (3H, s, CH_3_-19). ^13^C NMR (150 MHz, DMSO) δ: 193.78 (C15), 171.37 (C4′), 170.39 (C1′), 167.73 (C7), 146.47 (C16), 131.43 (Ar), 128.53 (Ar×3), 121.11 (C17), 113.64 (Ar×2) 106.59 (C1), 100.74 (C6), 78.92 (C14), 71.50 (C20), 55.06 (C8), 53.29 (C6′), 52.57 (C5), 51.94 (C10), 45.71 (C13), 41.51 (C5′), 38.33 (C9), 35.37 (C3), 32.59 (C4), 30.55 (C2), 29.44 (C2′), 29.29 (C3′), 27.03 (C12), 23.11 (C18), 22.76 (C19), and 19.02 (C11). HRMS (ESI) *m*/*z*: calculated for C_32_H_37_NO_9_ [M + Na]^+^, 602.2366, found, 602.2366.

**ent-6β,14α-Dihydroxy-(1,7–1,14)-epoxy-7,15-dioxo-6,20-hemiketal-6,7-seco-16-kaurene-6β-yl 4-((4-(tert-butyl)benz-yl)amino)-4-oxobutanoate (7d)**. Yield: 70%, white granule. ^1^H NMR (600 MHz, DMSO) values were as follows: δ 8.31 (1H, s, NH), 7.31 (2H, d, *J* = 8.3 Hz, H-Ar), 7.15 (2H, d, *J* = 8.3 Hz, H-Ar), 6.12 (2H, d, *J* = 13.6 Hz, CH_2_-17), 5.69 (1H, brs, H-6), 4.41 (1H, d, *J* = 4.5 Hz, H-14), 4.19 (2H, dd, *J* = 5.3, 3.7 Hz, CH_2_-5′), 4.07 (1H, d, *J* = 9.6 Hz, CH_2_-20), 3.52 (1H, d, *J* = 9.6 Hz, CH_2_-20), 3.29 (1H, m, H-13), 2.48 (1H, d, *J* = 3.7 Hz, CH_2_-2′), 2.37 (1H, d, *J* = 6.9 Hz, CH_2_-2′), 2.35 (1H, d, *J* = 6.5 Hz, CH_2_-3′), 2.16 (2H, m), 1.95 (1H, m), 1.86 (1H, d, *J* = 13.8 Hz), 1.70 (1H, m), 1.53 (2H, m), 1.46 (1H, d, *J* = 13.8 Hz), 1.26 (9H, s, CH_3_-7′, CH_3_-8′, CH_3_-9′), 1.23 (2H, s), 1.08 (3H, s, CH_3_-18), 1.00 (3H, s, CH_3_-19). ^13^C NMR (150 MHz, DMSO) δ: 193.78 (C15), 171.38 (C4′), 170.44 (C1′), 167.72 (C7), 149.13 (Ar), 146.48 (C16), 136.47 (Ar), 127.07 (Ar), 124.99 (Ar), 121.10 (C17), 106.59 (C1), 100.78 (C6), 78.93 (C14), 71.50 (C20), 53.29 (C8), 52.57 (C5), 51.94 (C10), 45.71 (C13), 41.79 (C5′), 38.34 (C9), 35.37 (C3), 34.15 (C6′), 32.59 (C4), 31.18 (C7′, C8′, C9′), 30.56 (C2), 29.42 (C2′), 29.30 (C3′), 27.04 (C12), 23.11 (C18), 22.75 (C19), and 19.03 (C11). HRMS (ESI) *m*/*z*: calculated for C_35_H_43_NO_8_ [M + H]^+^, 628.2886, found, 628.2890.

**ent-6β,14α-Dihydroxy-(1,7–1,14)-epoxy-7,15-dioxo-6,20-hemiketal-6,7-seco-16-kaurene-6β-yl 4-((4-fluorobenzyl) amino)-4-oxobutanoate (7e)**. Yield: 65%, white granule. ^1^H NMR (600 MHz, DMSO) values were as follows: δ 8.38 (1H, s, NH), 7.26 (2H, s, H-Ar), 7.12 (2H, s, H-Ar), 6.12 (2H, *J* = 16.6 Hz, CH_2_-17), 5.70 (1H, brs, H-6), 4.42 (1H, s, H-14), 4.22 (2H, s, CH_2_-5′), 4.06 (1H, d, *J* = 9.7 Hz, CH_2_-20), 3.52 (1H, d, *J* = 9.8 Hz, CH_2_-20), 3.30 (1H, m, H-13), 2.47 (1H, d, *J* = 6.2 Hz, CH_2_-3′), 2.37 (2H, d, *J* = 6.5 Hz, CH_2_-2′), 2.35 (1H, s, CH_2_-3′), 2.15 (2H, m), 1.98 (1H, m), 1.86 (1H, d, *J* = 13.4 Hz), 1.69 (1H, m), 1.54 (2H, m), 1.47 (1H, m), 1.23 (2H, s), 1.08 (3H, s, CH_3_-18), 0.99 (3H, s, CH_3_-19). ^13^C NMR (150 MHz, DMSO) δ: 193.80 (C15), 171.36 (C4′), 170.60 (C1′), 167.74 (C7), 161.95 (Ar), 160.35 (Ar), 146.48 (C16), 135.73 (Ar), 129.11 (Ar), 121.13 (C17), 115.02 (Ar), 114.88 (Ar), 106.60 (C1), 100.77 (C6), 78.94 (C14), 71.51 (C20), 53.29 (C8), 52.57 (C5), 51.94 (C10), 45.72 (C13), 41.34 (C5′), 38.34 (C9), 35.37 (C3), 32.59 (C4), 30.55 (C2), 29.43 (C2′), 29.25 (C3′), 27.04 (C12), 23.11 (C18), 22.76 (C19), and 19.03 (C11). HRMS (ESI) *m*/*z*: calculated for C_31_H_34_NO_8_F [M + Na]^+^, 590.2166, found, 590.2166.

**ent-6β,14α-Dihydroxy-(1,7–1,14)-epoxy-7,15-dioxo-6,20-hemiketal-6,7-seco-16-kaurene-6β-yl 4-((4-chlorobenzyl) amino)-4-oxobutanoate (7f).** Yield: 66%, white granule. ^1^H NMR (600 MHz, DMSO) values were as follows: δ 7.25-7.22 (2H, m, H-Ar), 7.00 (2H, t, *J* = 8.6 Hz, H-Ar), 6.30 (1H, s, CH_2_-17), 6.21 (1H, s, CH_2_-17), 5.92 (1H, s, NH), 5.57 (1H, brs, H-6), 4.39 (2H, dd, *J* = 14.8, 5.7 Hz, H-14, CH_2_-5′), 4.28 (1H, d, *J* = 5.7 Hz), 4.03 (1H, d, *J* = 9.8 Hz, CH_2_-20), 3.73 (1H, d, *J* = 9.8 Hz, CH_2_-20), 3.27 (1H, m, H-13), 2.71 (1H, m), 2.51 (1H, d, *J* = 10.6 Hz, CH_2_-3′), 2.47 (1H, d, *J* = 5.6 Hz, CH_2_-2′), 2.41 (1H, d, *J* = 9.3 Hz, CH_2_-3′), 2.25 (1H, d, *J* = 10.7 Hz, CH_2_-2′), 2.20 (1H, s), 2.08 (2H, d, *J* = 10.7 Hz), 2.02 (1H, d, *J* = 14.0 Hz), 1.81 (1H, m), 1.62 (1H, d, *J* = 13.0 Hz), 1.50 (1H, d, *J* = 13.9 Hz), 1.25 (2H, s), 1.14 (3H, s, CH_3_-18), 1.06 (3H, s, CH_3_-19). ^13^C NMR (150 MHz, DMSO) δ: 194.03 (C15), 171.81 (C4′), 171.04 (C1′), 168.28 (C7), 161.50 (Ar), 146.23 (C16), 134.02 (Ar), 129.58 (Ar), 129.52 (Ar), 121.83 (C17), 115.75 (Ar), 115.61 (Ar), 107.01 (C1), 101.61 (C6), 79.88 (C14), 72.67 (C20), 54.07 (C8), 53.24 (C5), 52.64 (C10), 46.31 (C13), 43.12 (C5′), 39.44 (C9), 35.97 (C3), 33.27 (C4), 31.03 (C2), 30.58 (C2′), 29.79 (C3′), 27.66 (C12), 23.61 (C18), 23.32 (C19), and 19.44 (C11). HRMS (ESI) *m*/*z*: calculated for C_31_H_34_NO_8_Cl [M + Na]^+^, 606.1871, found, 606.1871.

**ent-6β,14α-Dihydroxy-(1,7–1,14)-epoxy-7,15-dioxo-6,20-hemiketal-6,7-seco-16-kaurene-6β-yl 4-((4-bromobenzyl) amino)-4-oxobutanoate (7g).** Yield: 60%, white granule. ^1^H NMR (600 MHz, DMSO) values were as follows: δ 8.41 (1H, s, NH), 7.49 (2H, d, *J* = 8.4 Hz, H-Ar), 7.19 (2H, d, *J* = 8.4 Hz, H-Ar), 6.13 (1H, s, CH_2_-17), 6.10 (1H, s, CH_2_-17), 5.70 (1H, brs, H-6), 4.41 (1H, d, *J* = 4.4 Hz, H-14), 4.20 (2H, d, *J* = 3.5 Hz, CH_2_-5′), 4.06 (1H, d, *J* = 9.6 Hz, CH_2_-20), 3.51 (1H, d, *J* = 9.6 Hz, CH_2_-20), 3.30 (1H, m, H-13), 2.48 (1H, d, *J* = 5.2 Hz, CH_2_-3′), 2.37 (2H, d, *J* = 6.8 Hz, CH_2_-2′), 2.34 (1H, d, *J* = 5.2 Hz, CH_2_-3′), 2.15 (2H, m), 1.97 (1H, m), 1.85 (1H, d, *J* = 9.9 Hz), 1.69 (1H, m), 1.53 (2H, d, *J* = 12.6 Hz), 1.46 (1H, d, *J* = 13.8 Hz), 1.23 (2H, s), 1.07 (3H, s, CH_3_-18), 0.99 (3H, s, CH_3_-19). ^13^C NMR (150 MHz, DMSO) δ: 193.83 (C15), 171.37 (C4′), 170.72 (C1′), 167.76 (C7), 146.48 (C16), 139.09 (Ar), 131.11 (Ar), 129.43 (Ar), 121.16 (C17), 119.37 (Ar), 106.62 (C1), 100.78 (C6), 78.95 (C14), 71.51 (C20), 53.30 (C8), 52.59 (C5), 51.95 (C10), 45.73 (C13), 41.45 (C5′), 38.35 (C9), 35.38 (C3), 32.60 (C4), 30.56 (C2), 29.43 (C2′), 29.26 (C3′), 27.05 (C12), 23.12 (C18), 22.77 (C19), and 19.04 (C11). HRMS (ESI) *m*/*z*: calculated for C_31_H_34_NO_8_Br [M + Na]^+^, 650.1365, found, 650.1369.

**ent-6β,14α-Dihydroxy-(1,7–1,14)-epoxy-7,15-dioxo-6,20-hemiketal-6,7-seco-16-kaurene-6β-yl 4-(4-(6-fluorobenzo[d] isoxazol-3-yl) piperidin-1-yl)-4-oxobutanoate (7h).** Yield: 85%, white granule. ^1^H NMR (600 MHz, DMSO-d_6_) values were as follows: δ 8.05 (1H, dd, *J* = 8.7, 5.3 Hz, H-Ar), 7.70 (1H, d, *J* = 9.0 Hz, H-Ar), 7.29 (1H, t, *J* = 9.0 Hz, H-Ar), 6.12 (2H, s, CH_2_-17), 5.68 (1H, d, *J* = 11.3 Hz, H-6), 4.41 (2H, t, *J* = 8.7 Hz), 4.08 (1H, s, H-14), 3.96 (1H, d, *J* = 12.1 Hz, H-7′), 3.50 (2H, m, CH_2_-5′), 3.31 (1H, m), 3.20 (2H, m, CH_2_-9′), 2.80 (1H, s), 2.59 (2H, m, CH_2_-2′), 2.47 (1H, m, CH_2_-3′), 2.36 (1H, m, CH_2_-3′), 2.17 (2H, m, CH_2_-6′), 2.04 (2H, d, *J* = 14.8 Hz, CH_2_-8′), 2.00 (1H, m), 1.87 (1H, d, *J* = 13.9 Hz, 1H), 1.78 (1H, m), 1.70 (1H, m), 1.61 (1H, m), 1.55 (1H, d, *J* = 9.7 Hz), 1.46 (1H, d, *J* = 14.2 Hz), 1.23 (2H, s), 1.09 (3H, s, CH_3_-18), 1.00 (3H, s, CH_3_-19). ^13^C NMR (150 MHz, DMSO) δ: 193.83 (C15), 171.54 (C4′), 169.09 (C1′), 167.71 (C7), 163.11 (Ar), 162.89 (Ar), 160.94 (C10′), 146.48 (C16), 123.89 (Ar), 121.12 (C17), 117.15 (Ar), 112.67 (Ar), 106.60 (C1), 100.66 (C6), 97.50 (Ar), 78.91 (C14), 71.49 (C20), 53.30 (C8), 52.57 (C5), 51.92 (C10), 45.71 (C13), 44.51 (C5′), 41.03 (C9′), 38.32 (C9), 35.36 (C3), 33.23 (C7′), 32.61 (C4), 30.54 (C2), 30.17 (C6′), 29.73 (C8′), 29.38 (C12), 27.09 (C2′), 27.03 (C3′), 23.11 (C18), 22.80 (C19), and 19.09 (C11). HRMS (ESI) *m*/*z*: calculated for C_36_H_39_N_2_O_9_F [M + Na]^+^, 685.2537, found, 685.2542.

### 4.2. Biological Testing

#### 4.2.1. Cell Culture

Human non-small cell lung cancer cell lines (A549), human liver cancer cell lines (HepG2), human breast cancer cell lines (MCF-7), and human normal cell lines (L-02) were purchased from the cell bank of the Chinese Academy of Sciences. All cells were placed in RPMI-1640 medium (added 10% fetal bovine serum, 100 units/mL streptomycin, and 100 µg/mL penicillin) and cultured in an aseptic environment with 5% CO_2_ at 37 °C.

#### 4.2.2. In Vitro Anti-Proliferative Activity

All derivatives were evaluated for their anti-proliferative activities against three cancer cell lines and one normal cell line by MTT assay. The cells in the logarithmic growth phase were inoculated into 96-well plates (3 × 10^3^ cells/well) and incubated for 48 h in a sterile environment with 5% CO_2_ at 37 °C to make them attach via cell adhesion; then, the cells were co-incubated with target compounds for 48 h. After that, MTT solution (10 µL/well) was added to the cell culture medium to continue incubation for 4 h, following which the medium was replaced with DMSO (150 µL/well). The absorbance values at 490 nm were determined using an enzyme meter (Safire 2, Tecan, Männedorf, Switzerland) and further converted to half-inhibitory concentration values of these compounds.

#### 4.2.3. Experimental Method for Cell Cycle Analysis

A549 cells were inoculated into 24-well plates and attached via cell adhesion. Then, gradient concentrations (0, 2, 4, and 8 µM) of **7h** were added and the cells were co-incubated for 48 h. The cells were cleaned with ice-cold phosphate buffer solution (PBS), digested with trypsin, collected, and prepared as a cell suspension. The cell suspension was treated with PI/RNase staining solution, the data were gathered using a flow cytometer (FACS Calibur, BD, Milpitas, CA, USA), and the cell cycle distribution was analyzed using Flow Jo software. Three attempts were performed for each treatment to eliminate experimental bias.

#### 4.2.4. Experimental Methods for Apoptosis Analysis

##### Cell Apoptosis Morphological Detection

A549 cells were inoculated into 24-well plates and attached via cell adhesion. Then, gradient concentrations (0, 2, 4, and 8 µM) of **7h** were added and co-incubated for 48 h. The cells were cleaned with ice-cold PBS, stained with AO/EB, mixed, and then incubated in cells for 5 min in darkness at 25 °C. The apoptosis morphology of the cells in each group was observed and photographed using a laser confocal microscope (LSM710, Zeiss, Oberkochen, Germany).

##### Cell Apoptosis Rate Detection

A549 cells were inoculated into 6-well plates and attached via cell adhesion. Then, gradient concentrations (0, 2, 4, and 8 µM) of **7h** was added and co-incubated for 48 h. The cells were gathered and prepared as cell suspensions according to the method described in Section 4.2.3. The cell suspension was treated with Annexin V-FITC/PI staining solution, and the data were gathered using a flow cytometer (FACS Calibur, BD, Milpitas, CA, USA) and analyzed using Flow Jo software. Three attempts were performed for each treatment to eliminate experimental bias.

#### 4.2.5. Experimental Methods for ROS Measurement

##### Cell Morphological Detection

A549 cells were inoculated into 24-well plates and attached via cell adhesion. Then, gradient concentrations (0, 2, 4, and 8 µM) of **7h** were added and the cells were co-incubated for 48 h. The cells were cleaned with ice-cold PBS, stained with H_2_DCFDA, mixed, and incubated in cells for 20 min in darkness at 25 °C. The apoptosis morphology of the cells in each group was observed and photographed using a laser confocal microscope (LSM710, Zeiss, Oberkochen, Germany).

##### ROS Content Determination

A549 cells were inoculated into 6-well plates and attached via cell adhesion. Then, gradient concentrations (0, 2, 4, and 8 µM) of **7h** were added and the cells were co-incubated for 48 h. The cells were gathered and prepared as cell suspensions according to the method described in Section 4.2.3. The cell suspensions were treated with H_2_DCFDA staining solution in darkness for 20 min at 25 °C and then cleaned with PBS to remove residual stain. The experimental data were gathered using flow cytometry (FACS Calibur, BD, Milpitas, CA, USA) and analyzed using Flow Jo software. Three attempts were performed for each treatment to eliminate experimental bias.

#### 4.2.6. Experimental Method for MMP Measurement

##### Cell Morphological Detection

A549 cells were inoculated into 24-well plates and attached via cell adhesion. Then, gradient concentrations (0, 2, 4, and 8 µM) of compound **7h** were added and the cells were co-incubated for 48 h. The cells were cleaned with ice-cold PBS, stained with JC-1, mixed, and then incubated in cells for 20 min in darkness at 25 °C. The apoptotic morphology of the cells in each group was observed and photographed using a laser confocal microscopy (LSM710, Zeiss, Oberkochen, Germany).

##### Cell Morphological Detection

A549 cells were inoculated into 6-well plates and attached via cell adhesion. Then, gradient concentrations (0, 2, 4, and 8 µM) of **7h** were added and the cells were co-incubated for 48 h. The cells were collected and prepared as cell suspensions according to the method described in Section 4.2.3. Cell suspensions were treated with JC-1 staining solution in darkness for 20 min at 25 °C, and then washed with PBS buffer to remove residual stain. The experimental data were gathered using flow cytometry (FACS Calibur, BD, Milpitas, CA, USA) and analyzed by Flow Jo software. Three attempts were performed for each treatment to eliminate experimental bias.

#### 4.2.7. Experimental Methods for GO and KEGG Analysis

The potential targets of compound **7h** were obtained from the PharmMapper online platform (http://www.lilab-ecust.cn/pharmmapper (accessed on 27 July 2024)). Firstly, the chemical structure of **7h** was constructed using ChemBio3D Ultra 14.0 software, and the energy minimum conformation was obtained using the “Calculations-MM2-Minemize Energy” module and saved in MOL2 format. The 3D structure was imported into the PharmMapper server, and the following parameters were qualified: generate conformers: yes; maximum generated conformations: 300; select target set: human protein target only; number of reserved matched targets: 300. The proteins (Z-score > 0.5) were screened as candidate targets for **7h**. The GO and KEGG analyses were performed using the DAVID platform (https://david.ncifcrf.gov (accessed on 27 July 2024)). R (4.4.1) software was used to analyze and visualize the results.

#### 4.2.8. Experimental Method of Western Blot Analysis

Western blot was used to determine the effect of **7h** against the related protein expression in A549 cells. A549 cells were inoculated in 6-well plates and treated with a grdient concentrations (0, 2, 4, and 8 µM) of **7h** for 48 h. The cells were collected, lysed in RIPA buffer, and heated at 100 °C for 10 min. The samples were processed via 10% sodium dodecyl sulfate-polyacrylamide gel electrophoresis (10% SDS-PAGE) and then transferred to a poly vinylidene difluoride (PVDE) membrane. The membrane was immersed in tris-buffered saline and tween (TBST) containing 5% skimmed milk and blocked for 2 h at 25 °C, and then incubated overnight at 4 °C with the addition of the internal reference protein primary antibody (GAPDH; dilution ratio 1:5000) and the target protein primary antibody (PI3K, p-PI3K, Akt, p-Akt, mTOR, and p-mTOR; dilution ratio 1:1000). Following this, the membrane was incubated with an HRP-labeled (horseradish peroxidase) secondary antibody for 2 h at 25 °C and positive bands were shown on X-ray film using an enhanced chemiluminescence system (Kodak, Rochester, NY, USA). The information on the primary antibodies is as follow: PI3K (Catalog: 4292, Cell Signaling Technology, Danvers, MA, USA), p-PI3K (Catalog: 4228, Cell Signaling Technology), Akt (Catalog: 9272, Cell Signaling Technology), p-Akt (Catalog: 4060, Cell Signaling Technology), mTOR (Catalog: 2972, Cell Signaling Technology), p-mTOR (Catalog: 5536, Cell Signaling Technology), and GAPDH (Catalog: 2118, Cell Signaling Technology).

#### 4.2.9. Experimental Method of Molecular Docking Study

The 3D model of ligand compounds was constructed using the ChemBio3D Ultra 14.0 software. The 3D structural models of receptor proteins PI3Kα (PDB ID: 6GVF), Akt1 (PDB ID: 6HHF), and mTOR (PDB ID: 4JT6) were downloaded from the RCSB Protein Data Bank (https://www.rcsb.org/ (accessed on 27 July 2024)) and reprocessed using the Pymol (3.0) software. Molecular docking studies were performed using the Autodock Vina 1.1.2 software [44]. Visualization and interaction analysis of the docking results were obtained using the Discovery Studio 2019 Client (V19.10) software [45].

## Figures and Tables

**Table 1:** In vitro anti-proliferative activities of **5a**–**m** and **7a**–**h** in different cell lines.

Compounds	IC_50_ (µM) ^a^			
	A549	HepG2	MCF-7	L-02
**5a**	14.72 ± 1.54	17.12 ± 1.39	30.72 ± 1.16	>50.00
**5b**	15.84 ± 1.50	15.22 ± 1.58	17.57 ± 2.40	>50.00
**5c**	20.25 ± 1.46	22.54 ± 3.42	21.87 ± 2.72	>50.00
**5d**	8.76 ± 1.56	11.04 ± 1.45	15.56 ± 1.61	30.36 ± 3.44
**5e**	10.71 ± 1.81	11.57 ± 1.96	14.11 ± 2.35	36.68 ± 2.43
**5f**	13.43 ± 2.19	18.30 ± 1.36	20.71 ± 1.20	45.88 ± 2.30
**5g**	15.26 ± 2.40	21.10 ± 2.50	22.81 ± 2.13	>50.00
**5h**	18.37 ± 1.13	20.18 ± 1.33	24.04 ± 2.55	>50.00
**5i**	14.39 ± 1.42	23.65 ± 3.40	25.12 ± 3.34	38.15 ± 2.82
**5j**	23.30 ± 2.25	34.07 ± 3.52	32.84 ± 2.95	>50.00
**5k**	17.16 ± 1.43	25.68 ± 2.16	26.27 ± 2.30	>50.00
**5l**	8.30 ± 2.55	11.15 ± 1.82	15.87 ± 1.90	>50.00
**5m**	6.85 ± 1.68	10.87 ± 1.25	12.34 ± 1.11	32.51 ± 1.62
**7a**	10.21 ± 1.10	13.77 ± 2.19	10.80 ± 1.65	>50.00
**7b**	7.48 ± 1.31	10.53 ± 1.68	12.76 ± 1.64	>50.00
**7c**	8.37 ± 1.53	12.83 ± 1.73	8.35 ± 1.96	34.42 ± 4.37
**7d**	6.41 ± 1.90	11.54 ± 1.58	7.64 ± 2.52	35.74 ± 2.66
**7e**	7.12 ± 1.52	11.60 ± 1.34	9.85 ± 1.53	>50.00
**7f**	7.38 ± 0.96	8.61 ± 1.14	8.20 ± 1.21	>50.00
**7g**	8.60 ± 2.65	8.78 ± 1.46	13.00 ± 1.52	>50.00
**7h**	2.16 ± 1.40	6.10 ± 1.24	6.74 ± 1.94	>50.00
**4**	23.83 ± 1.86	27.35 ± 0.83	25.75 ± 1.14	30.25 ± 1.58
Oridonin	21.10 ± 1.58	22.62 ± 1.20	18.46 ± 1.37	23.62 ± 1.65
Taxol	4.21 ± 0.73	6.60 ± 1.61	5.15 ± 0.80	8.31 ± 1.82

^a^ IC_50_: Concentration of the tested compound that inhibits 50% of cell growth. All data are recorded as means ± SD of three experiments.

**Table 2:** The docking results of compounds **7h** and **4**.

Compound	Receptor Protein	Binding Energy (kcal/mol) ^a^
**7h**	PI3Kα	−11.6
	Akt1	−11.3
	mTOR	−11.4
**4**	PI3Kα	−10.1
	Akt1	−9.0
	mTOR	−10.4

^a^ Binding energy: The lower the binding energy value, the better the incorporation between the compound and the protein.

## Data Availability

The data will be provided upon request.

## Supplementary Materials

The following supporting information can be downloaded at: https://www.mdpi.com/article/10.3390/molecules29174066/s1. The supplementary material includes HRMS, ^1^H NMR, and ^13^C NMR spectral data of all derivatives, the single-crystal data and absolute configuration of compound **4**, the GO and KEGG analyses of **7h**, and the molecular docking results.
