# Supplementary material for: Design, Synthesis, and Anticancer Activity of Novel Enmein-Type Diterpenoid Derivatives Targeting the PI3K/Akt/mTOR Signaling Pathway
Source: Molecules. 2024 Aug 27;29(17):4066. doi: 10.3390/molecules29174066 (PMC11396751; doi:10.3390/molecules29174066)

**Supplemental material**

Design, synthesis, and anticancer activity of novel enmein-type diterpenoid derivatives targeting the PI3K/Akt/mTOR signaling pathway

Jiafeng Wang^1, a^, Lu Wang^2, a^, Yingbo Zhang^1^, Siwen Pan^1^, Yu Lin^2^, Jiale Wu^3^, and Ming Bu^2*^

|  |
| --- |

^1^ College of Pathology, Qiqihar Medical University, Qiqihar, Heilongjiang, P.R. China; [wangjiafeng410323@163.com](mailto:wangjiafeng410323@163.com) (J.W.); [448508507@qq.com](mailto:448508507@qq.com) (Y.Z.); [siwen_tongtong0606@163.com](mailto:siwen_tongtong0606@163.com) (S.P.)

^2^ College of Pharmacy, Qiqihar Medical University, Qiqihar, Heilongjiang, P.R. China; [wanglu9264582022@163.com](mailto:wanglu9264582022@163.com) (L.W.); [linyu7373@163.com](mailto:linyu7373@163.com) (Y.L.); [buming@qmu.edu.cn](mailto:buming@qmu.edu.cn) (M.B.)

^3^ College of Life and Health, Hainan University, Haikou, Hainan, P.R. China; [wwwwjl55@163.com](mailto:wwwwjl55@163.com) (J.W.)

^a^ These authors contributed equally to this work.

***** Correspondence: buming@qmu.edu.cn; Tel.: +86-0452-2663-881.

**List of contents:**

^1^H NMR, ^13^C NMR and HRMS spectra of all synthesized novel compounds **03**

The potential target proteins selected of **7h**  **49**

The GO enrichment analysis of **7h 50**

The KEGG enrichment analysis of **7h**  **53**

The single-crystal data and absolute configuration of compound **4**  **54**

^1^H NMR, ^13^C NMR and HRMS spectra of compound **4**.

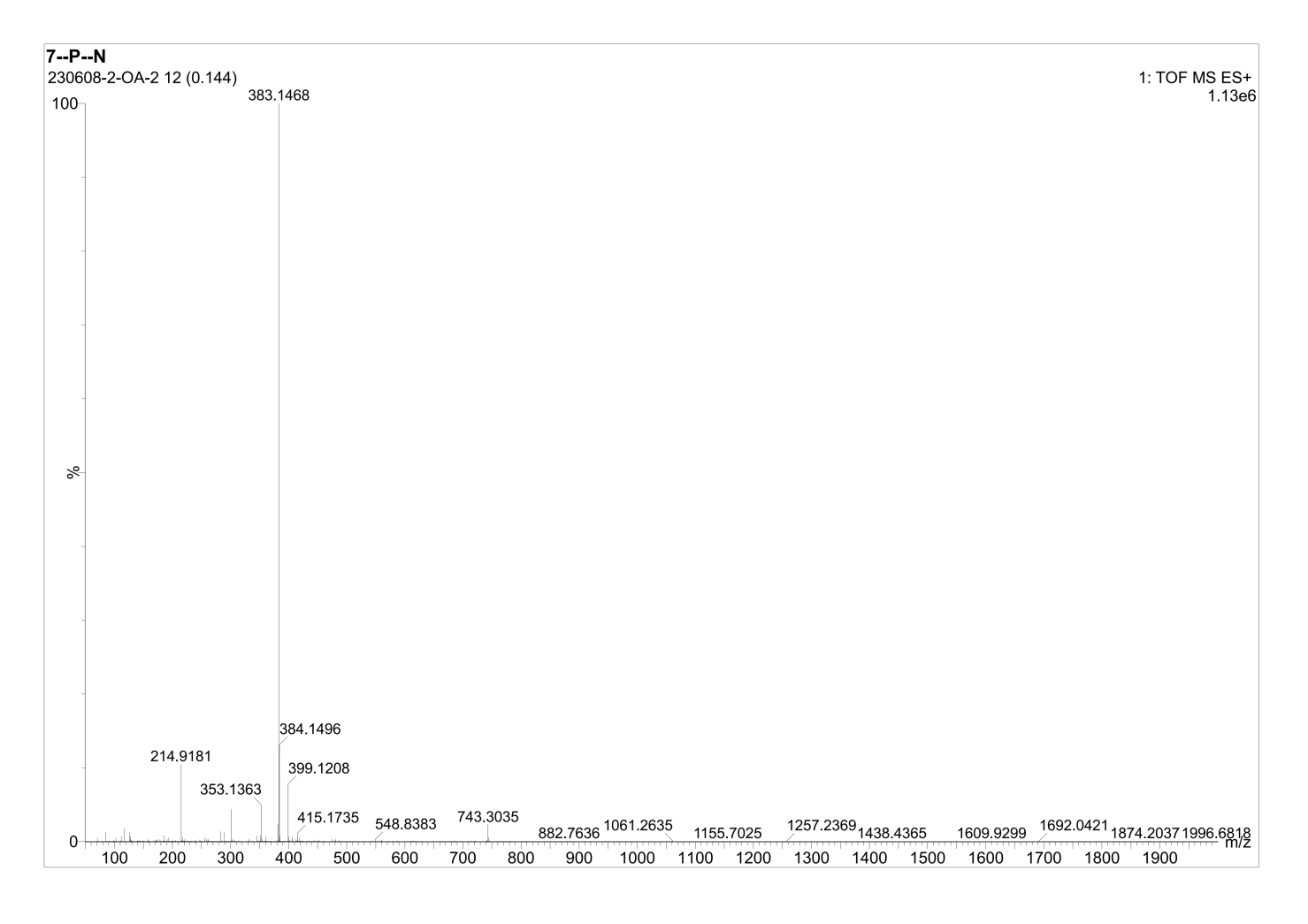

^1^H NMR, ^13^C NMR and HRMS spectra of compound **5a**.

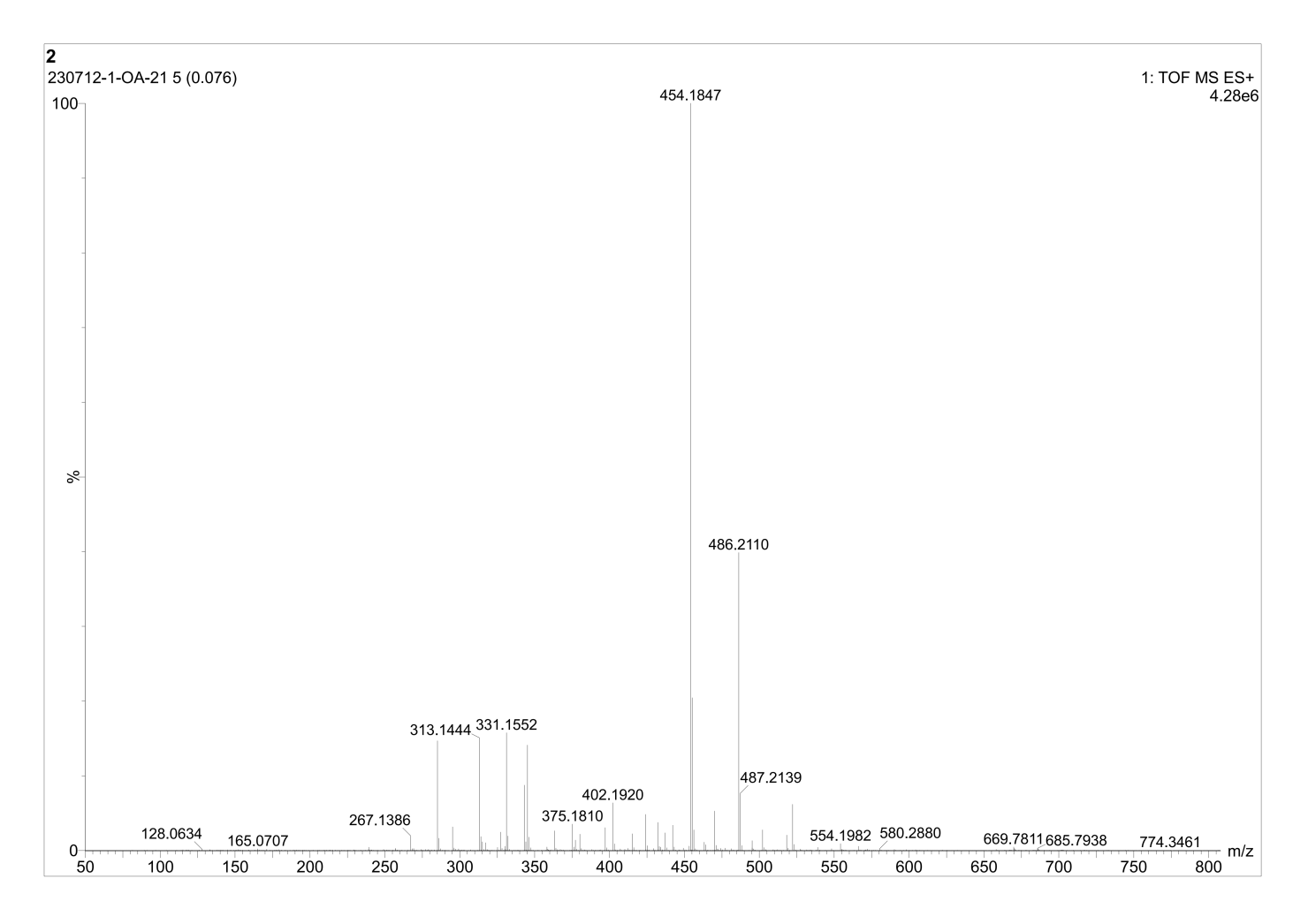

^1^H NMR, ^13^C NMR and HRMS spectra of compound **5b**.

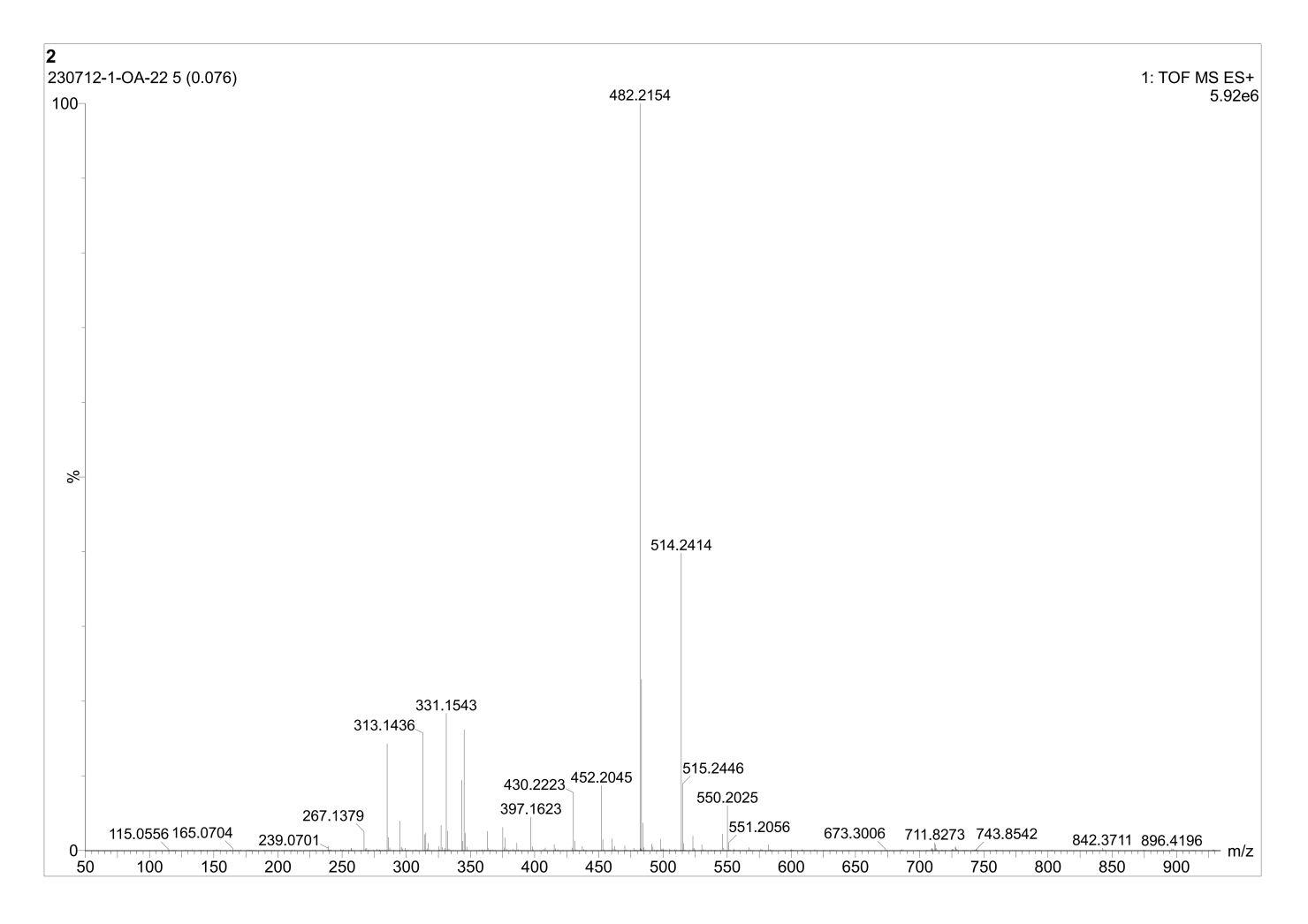

^1^H NMR, ^13^C NMR and HRMS spectra of compound **5c**.

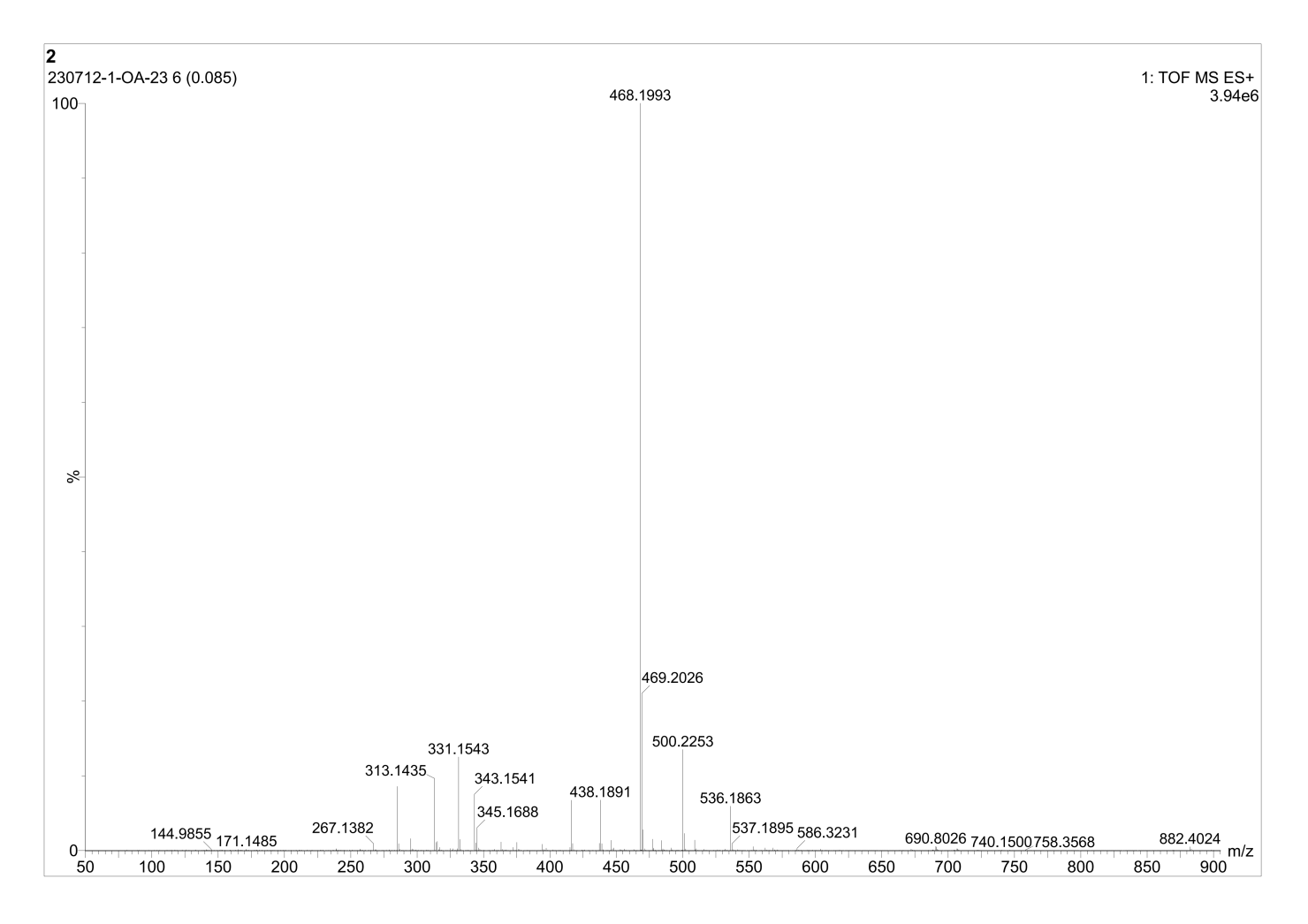

^1^H NMR, ^13^C NMR and HRMS spectra of compound **5d**.

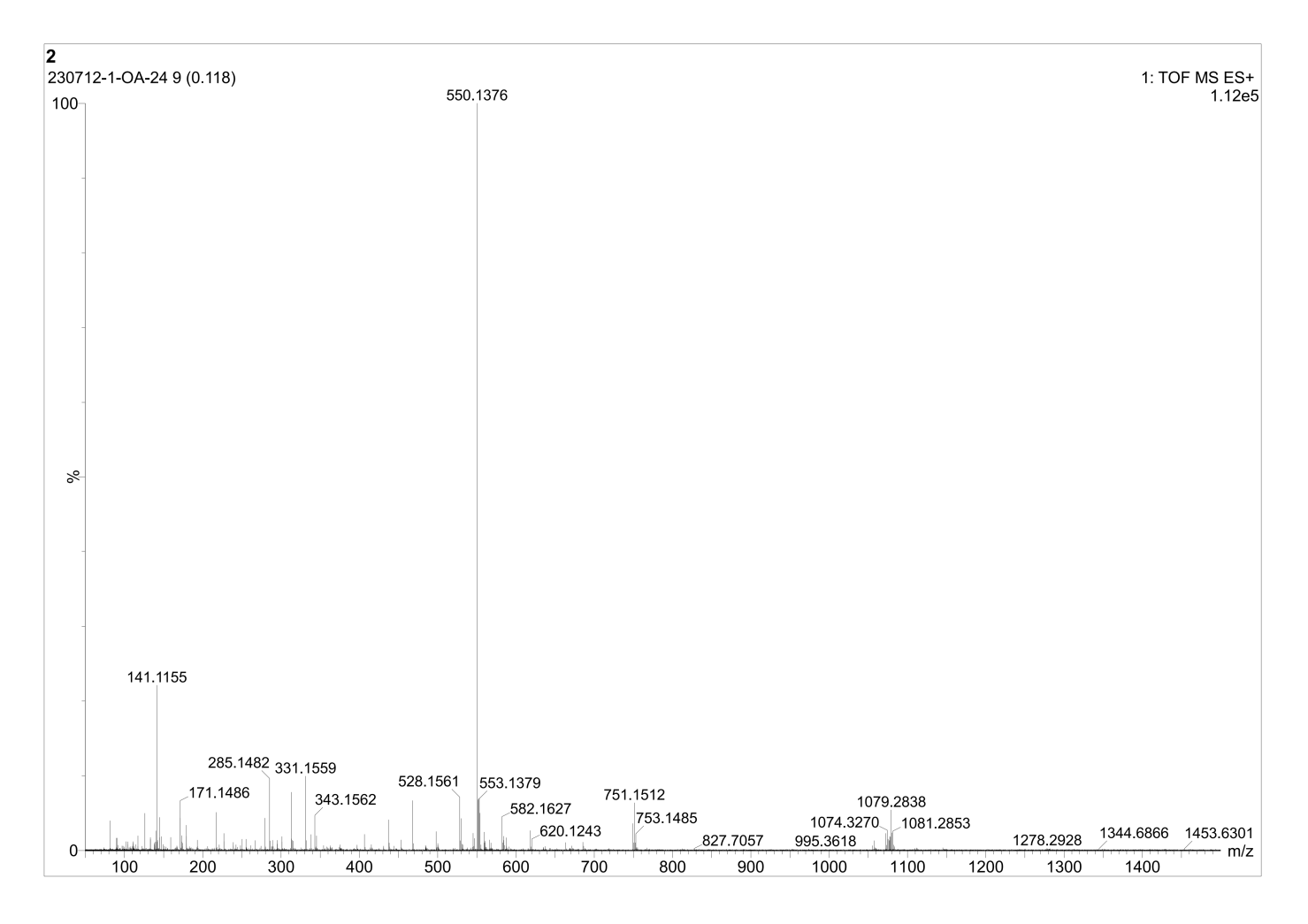

^1^H NMR, ^13^C NMR and HRMS spectra of compound **5e**.

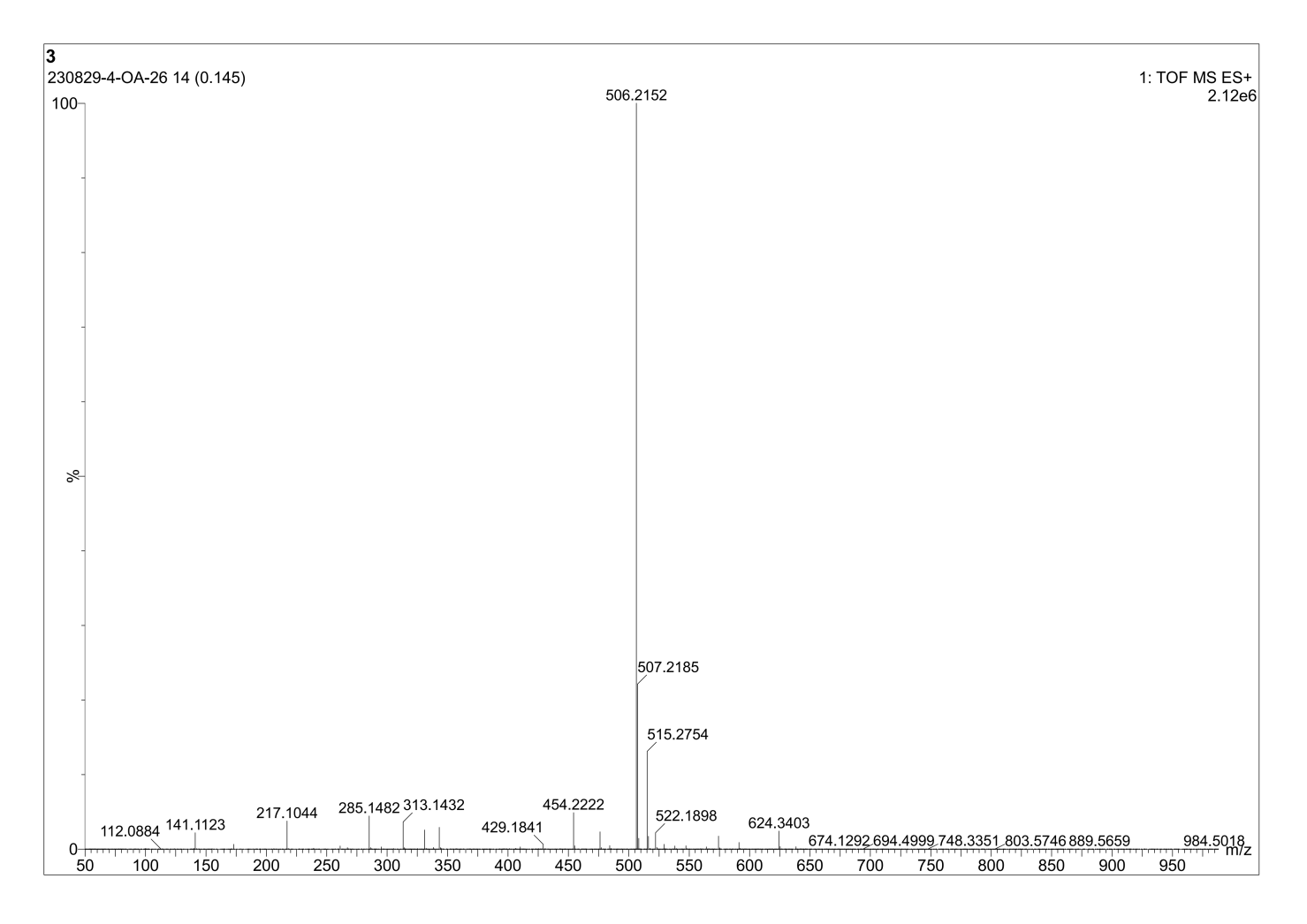

^1^H NMR, ^13^C NMR and HRMS spectra of compound **5f**.

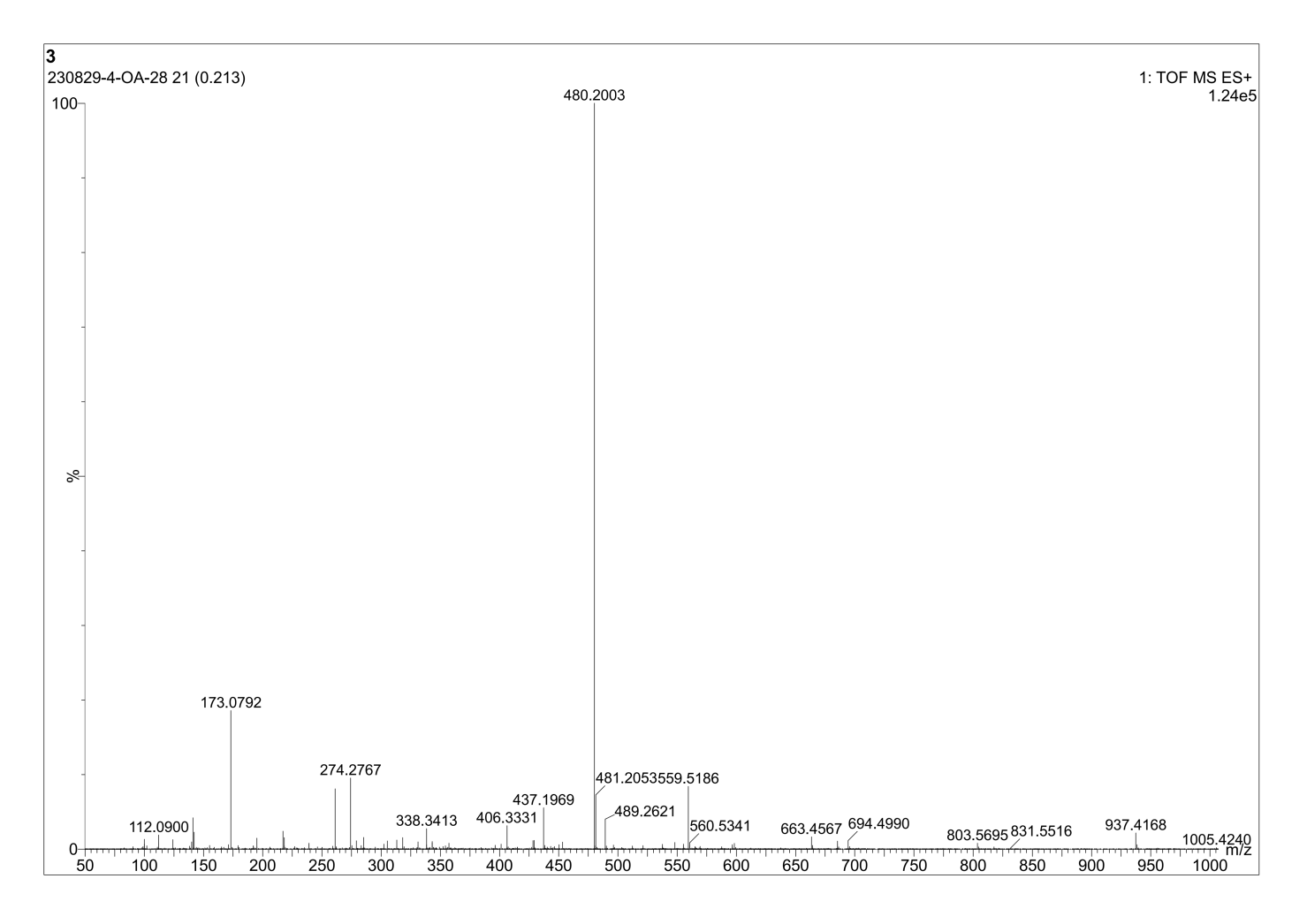

^1^H NMR, ^13^C NMR and HRMS spectra of compound **5g**.

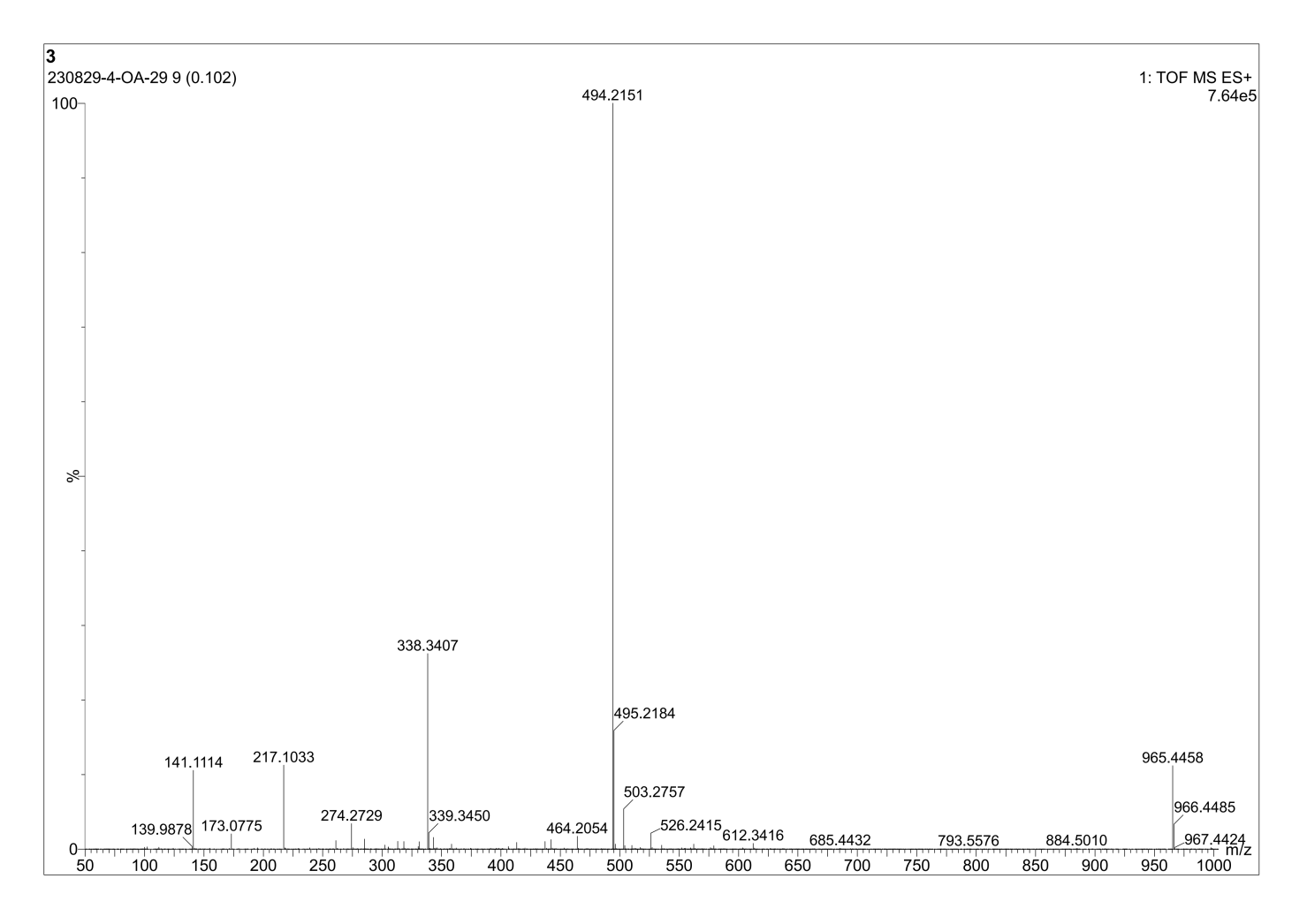

^1^H NMR, ^13^C NMR and HRMS spectra of compound **5h**.

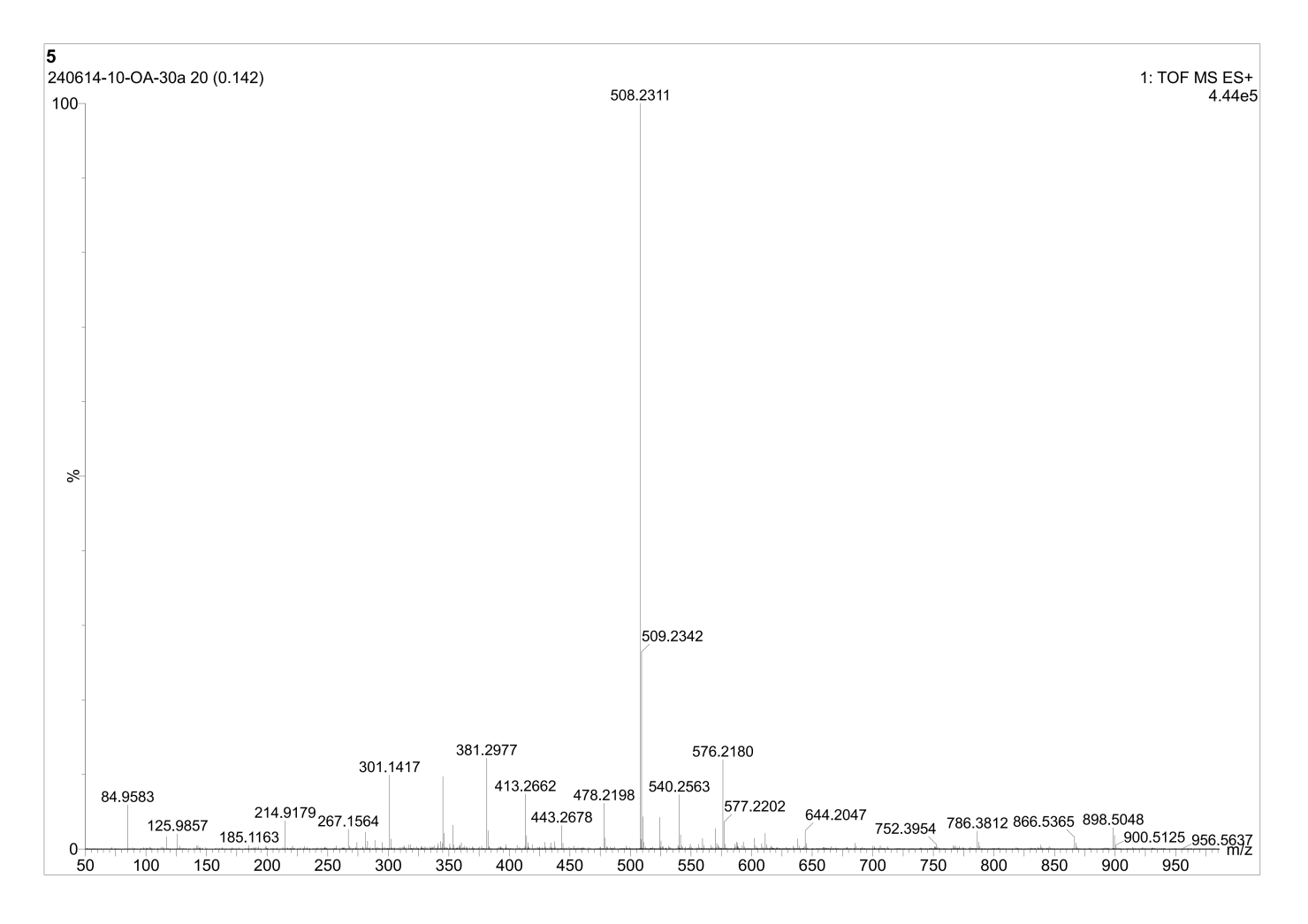

^1^H NMR, ^13^C NMR and HRMS spectra of compound **5i**.

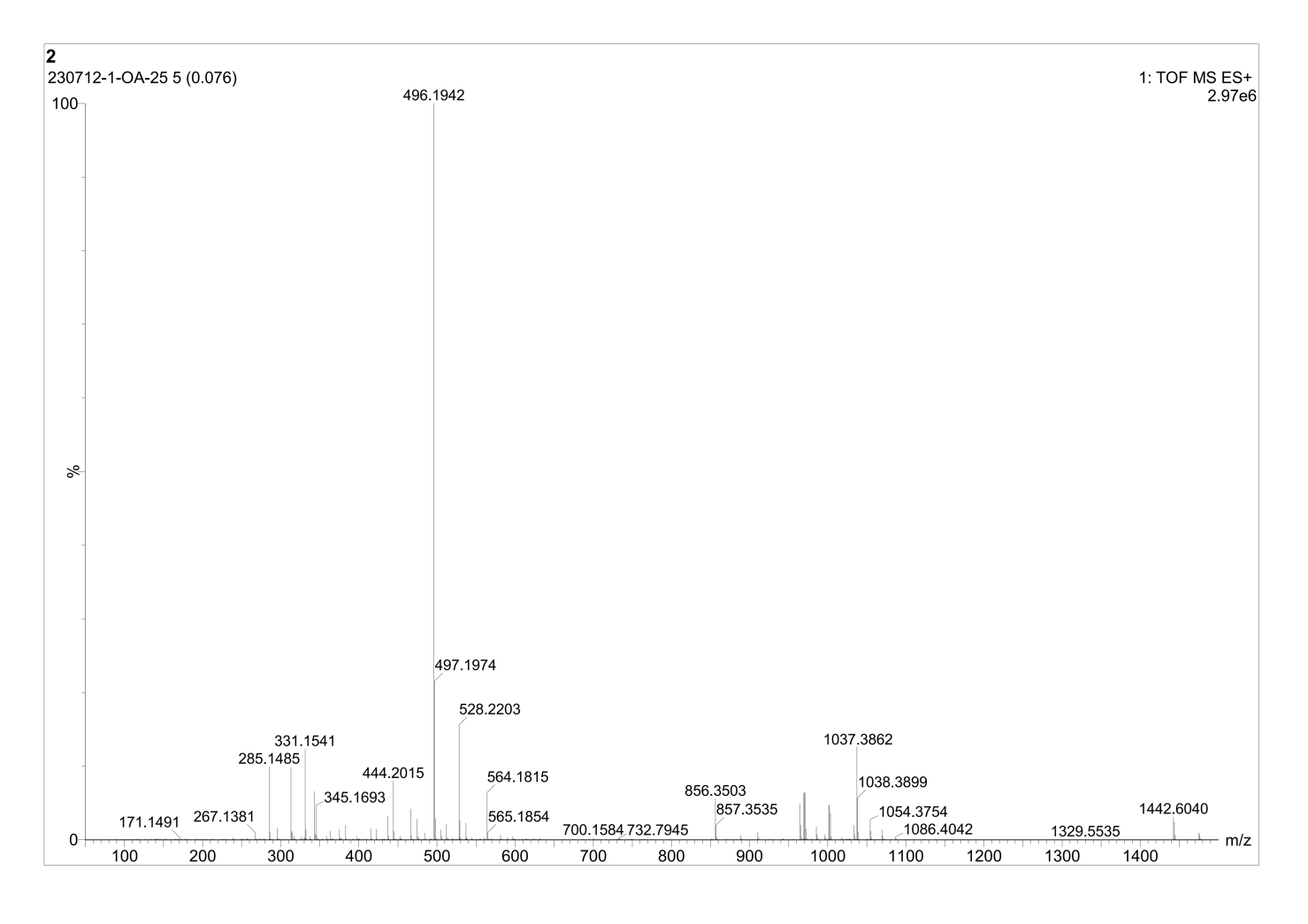

^1^H NMR, ^13^C NMR and HRMS spectra of compound **5j**.

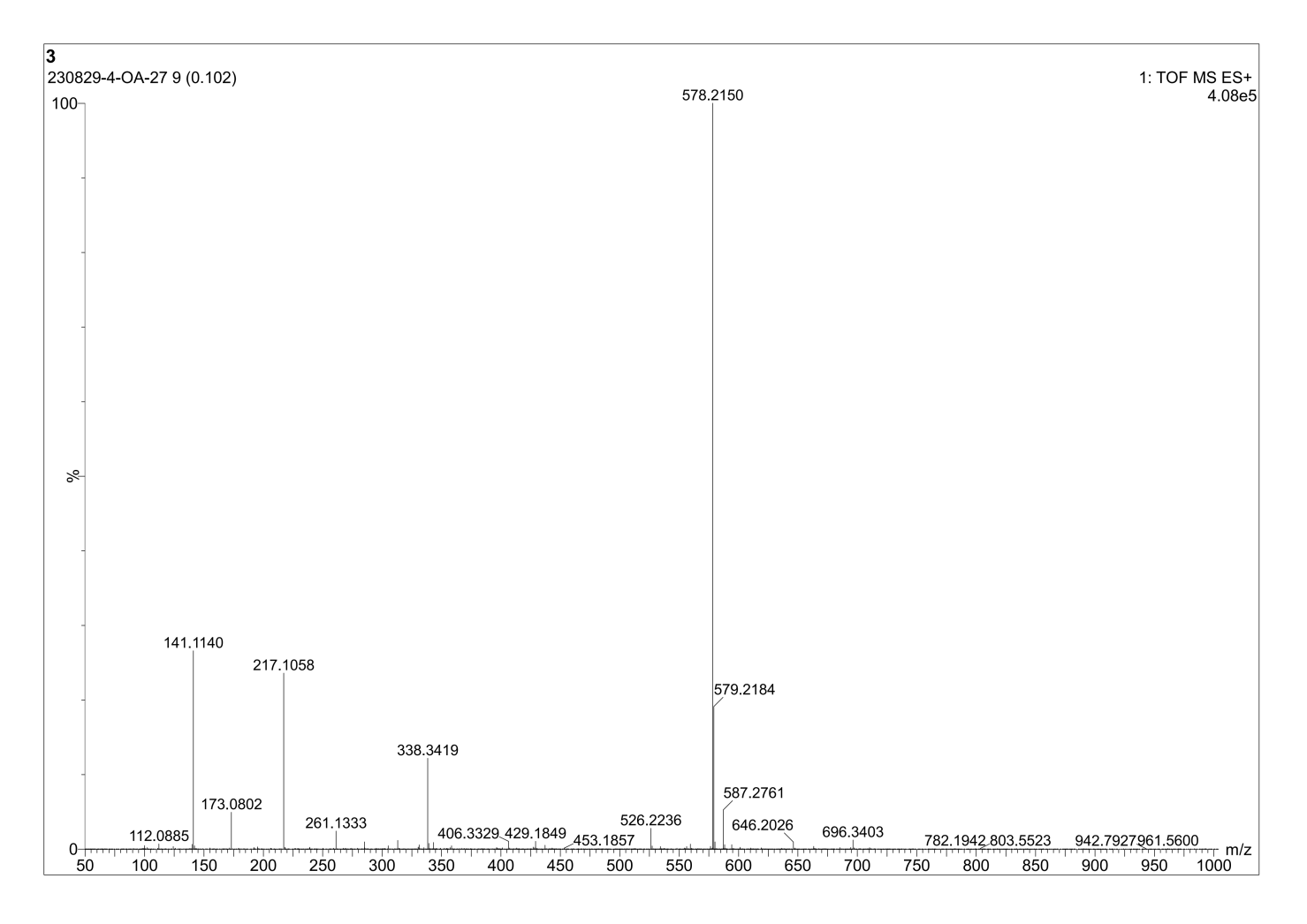

^1^H NMR, ^13^C NMR and HRMS spectra of compound **5k**.

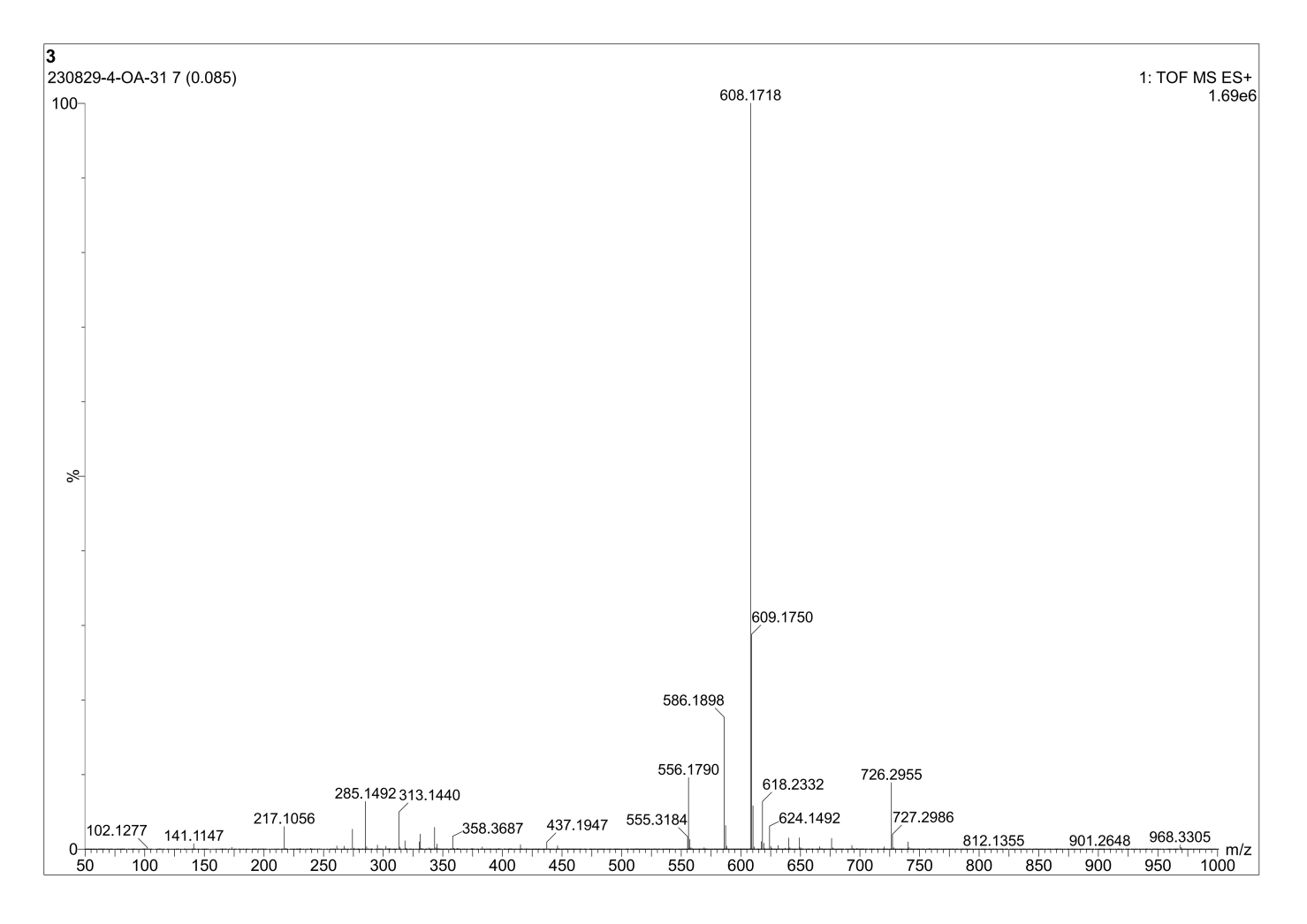

^1^H NMR, ^13^C NMR and HRMS spectra of compound **5l**.

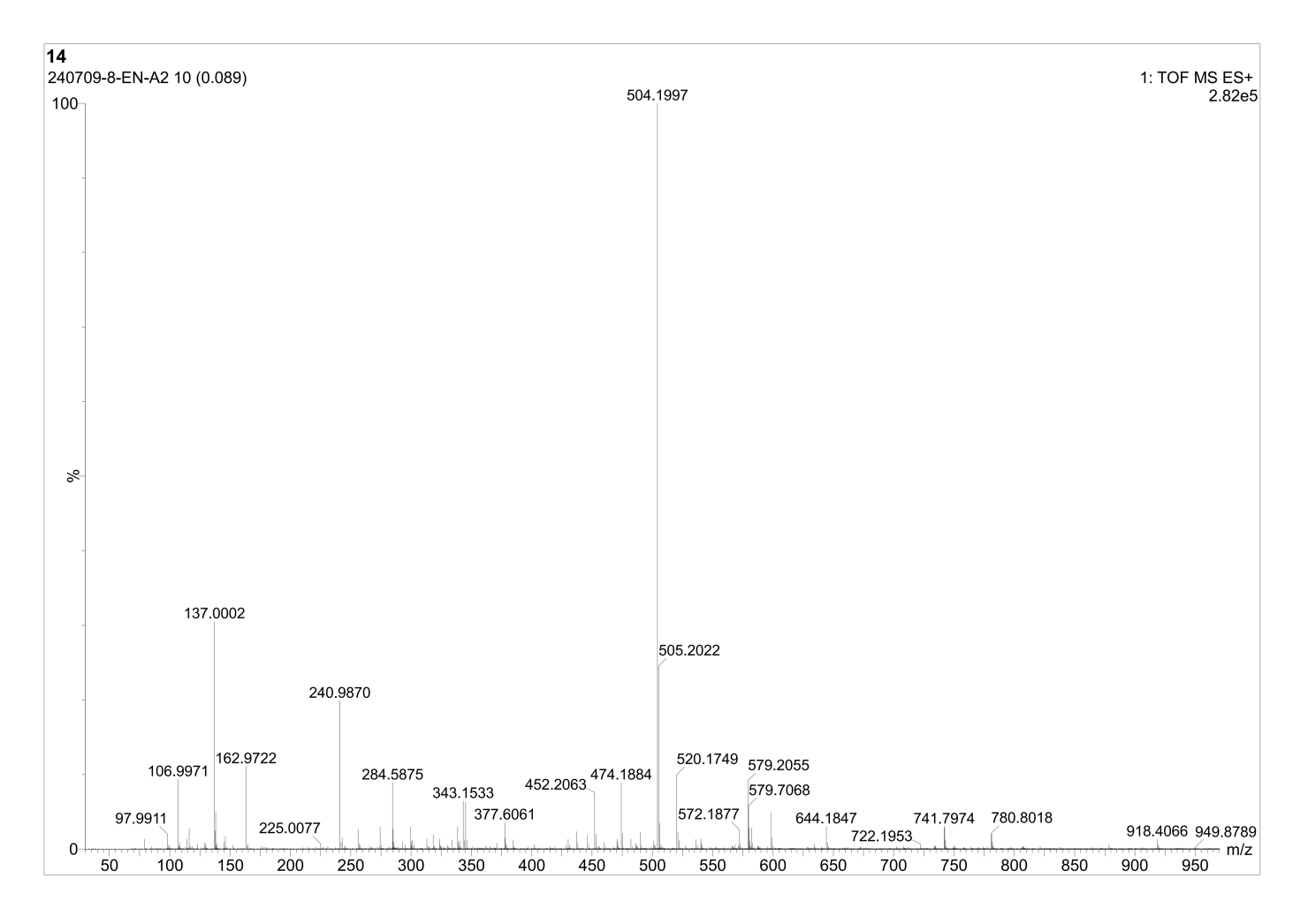

^1^H NMR, ^13^C NMR and HRMS spectra of compound **5m**.

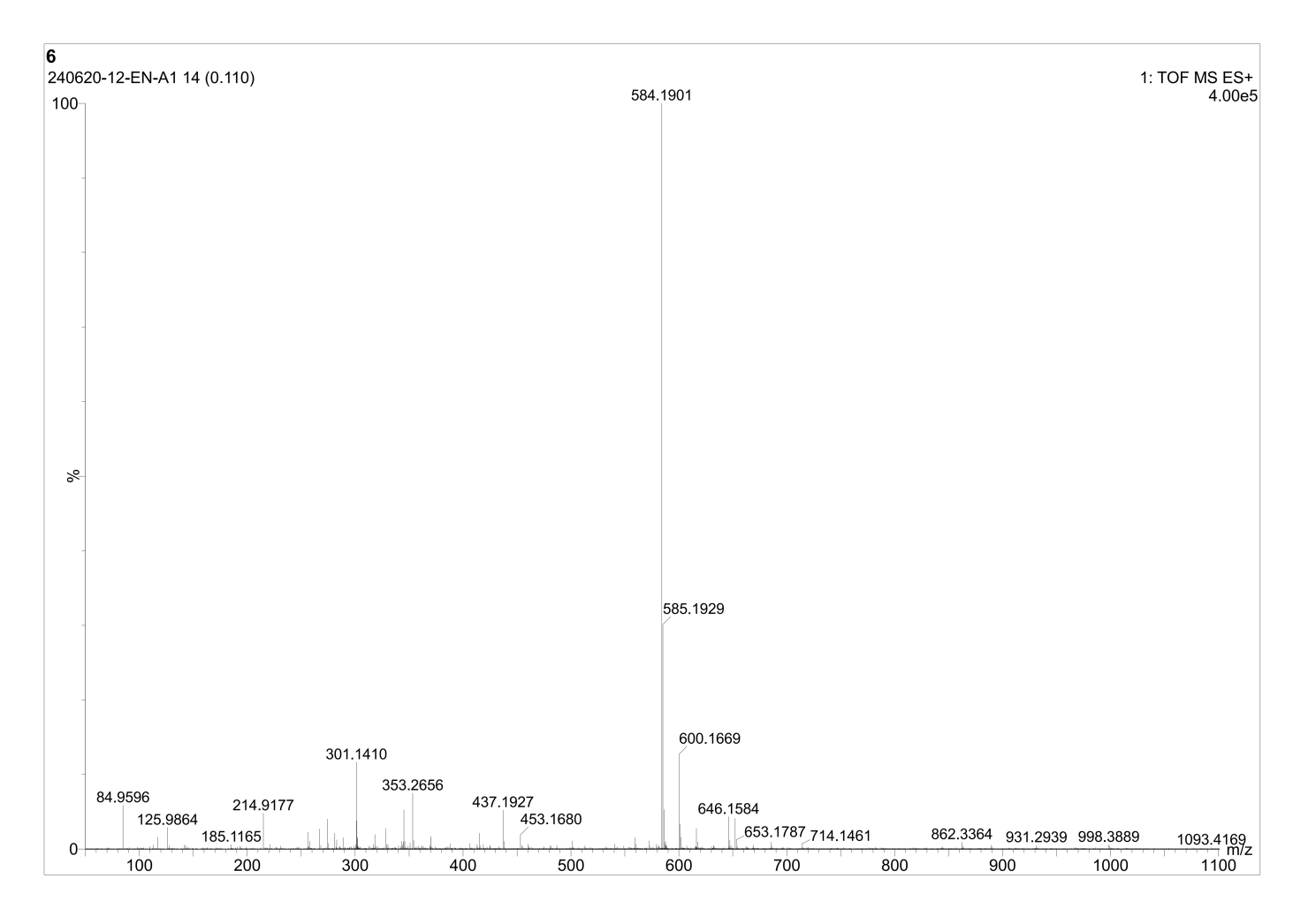

^1^H NMR, ^13^C NMR and HRMS spectra of compound **6**.

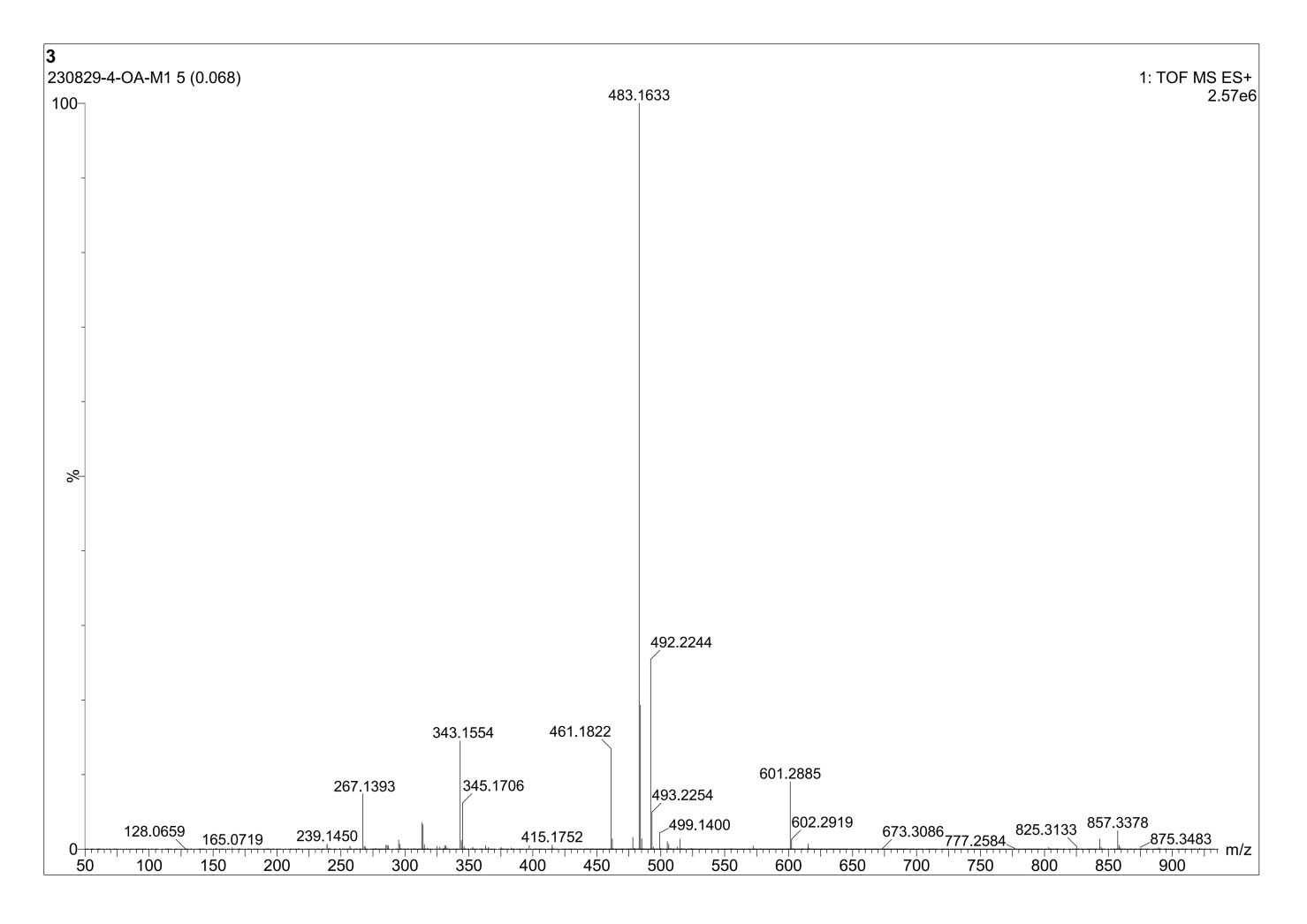

^1^H NMR, ^13^C NMR and HRMS spectra of compound **7a**.

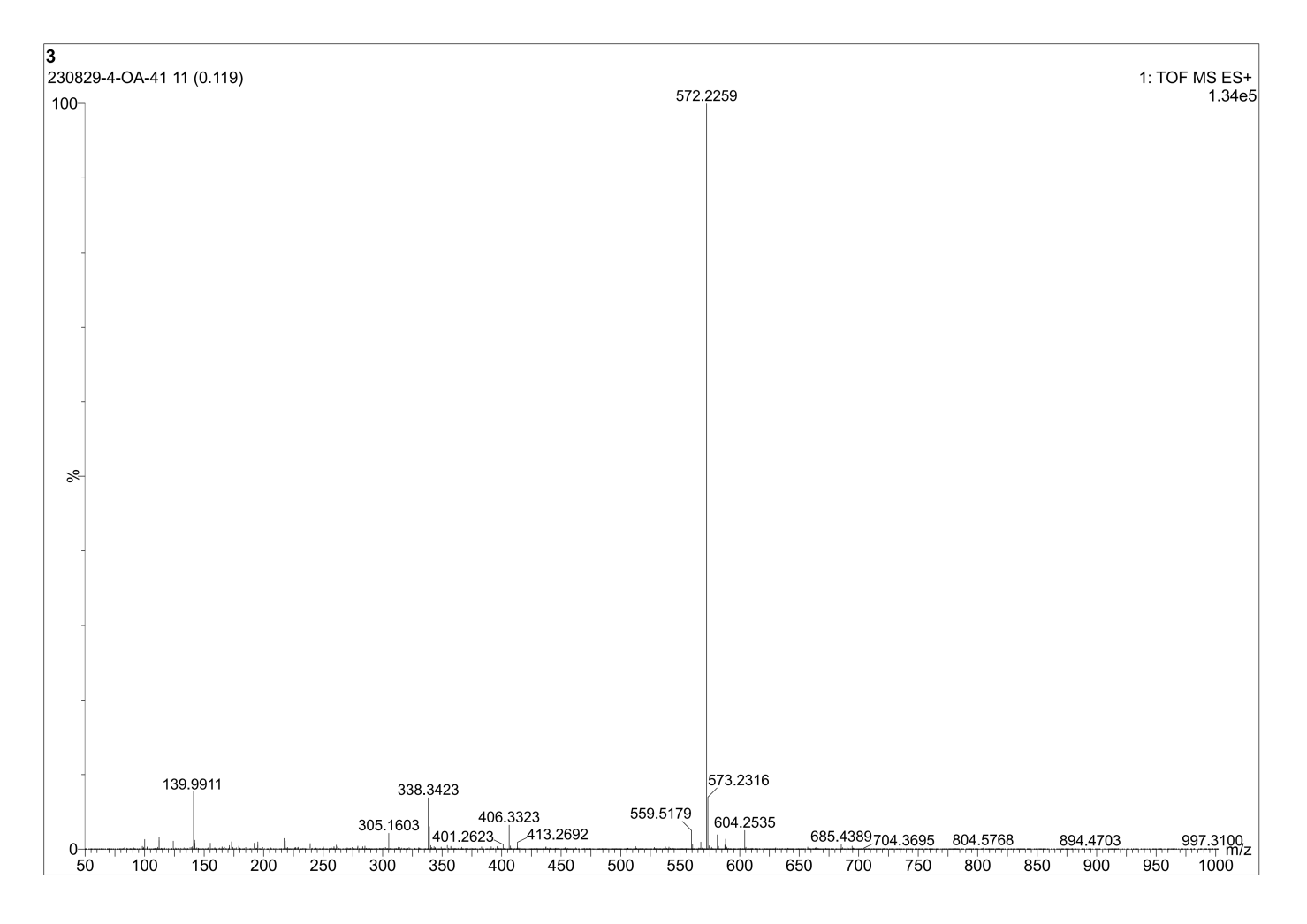

^1^H NMR, ^13^C NMR and HRMS spectra of compound **7b**.

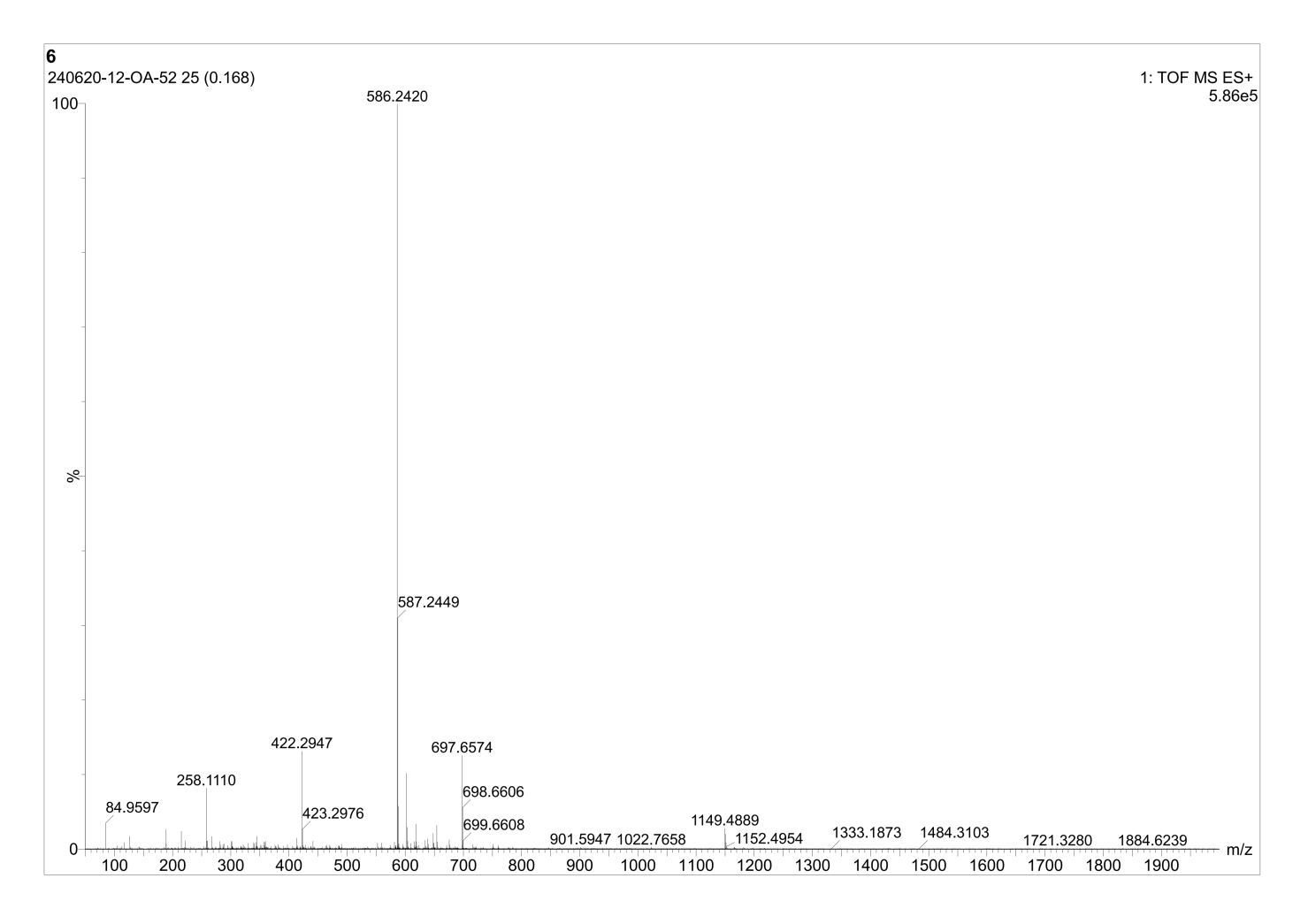

^1^H NMR, ^13^C NMR and HRMS spectra of compound **7c**.

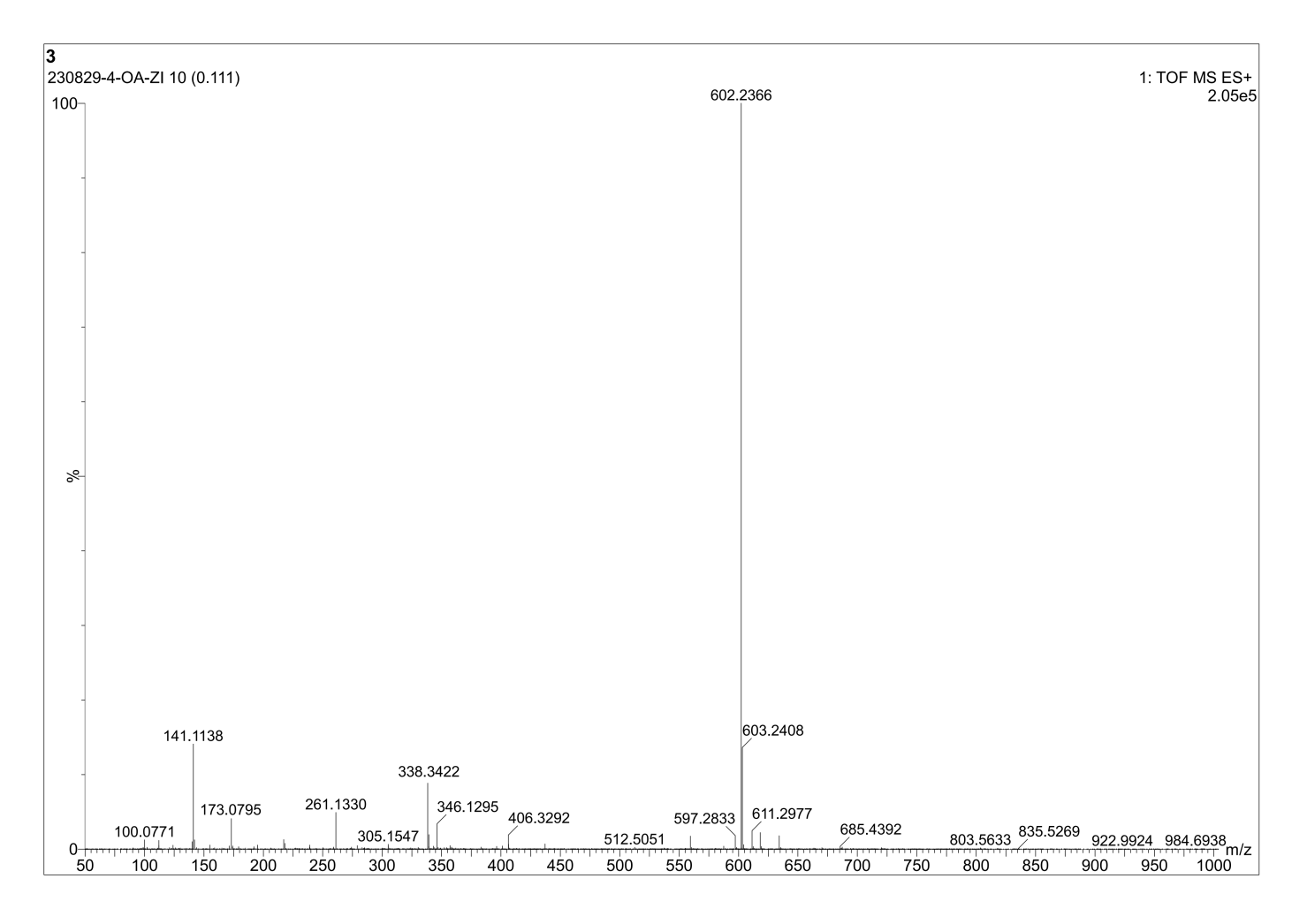

^1^H NMR, ^13^C NMR and HRMS spectra of compound **7d**.

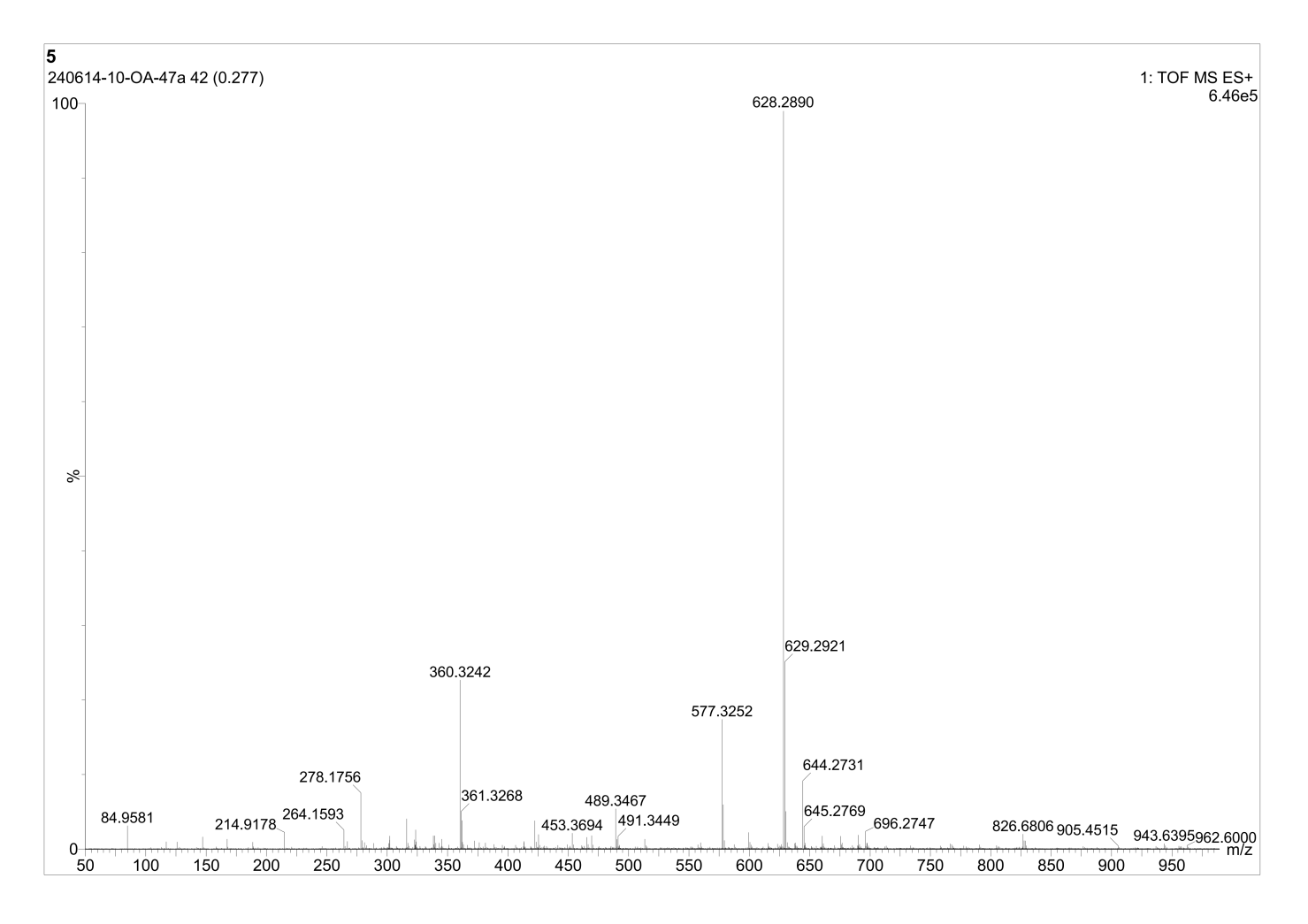

^1^H NMR, ^13^C NMR and HRMS spectra of compound **7e**.

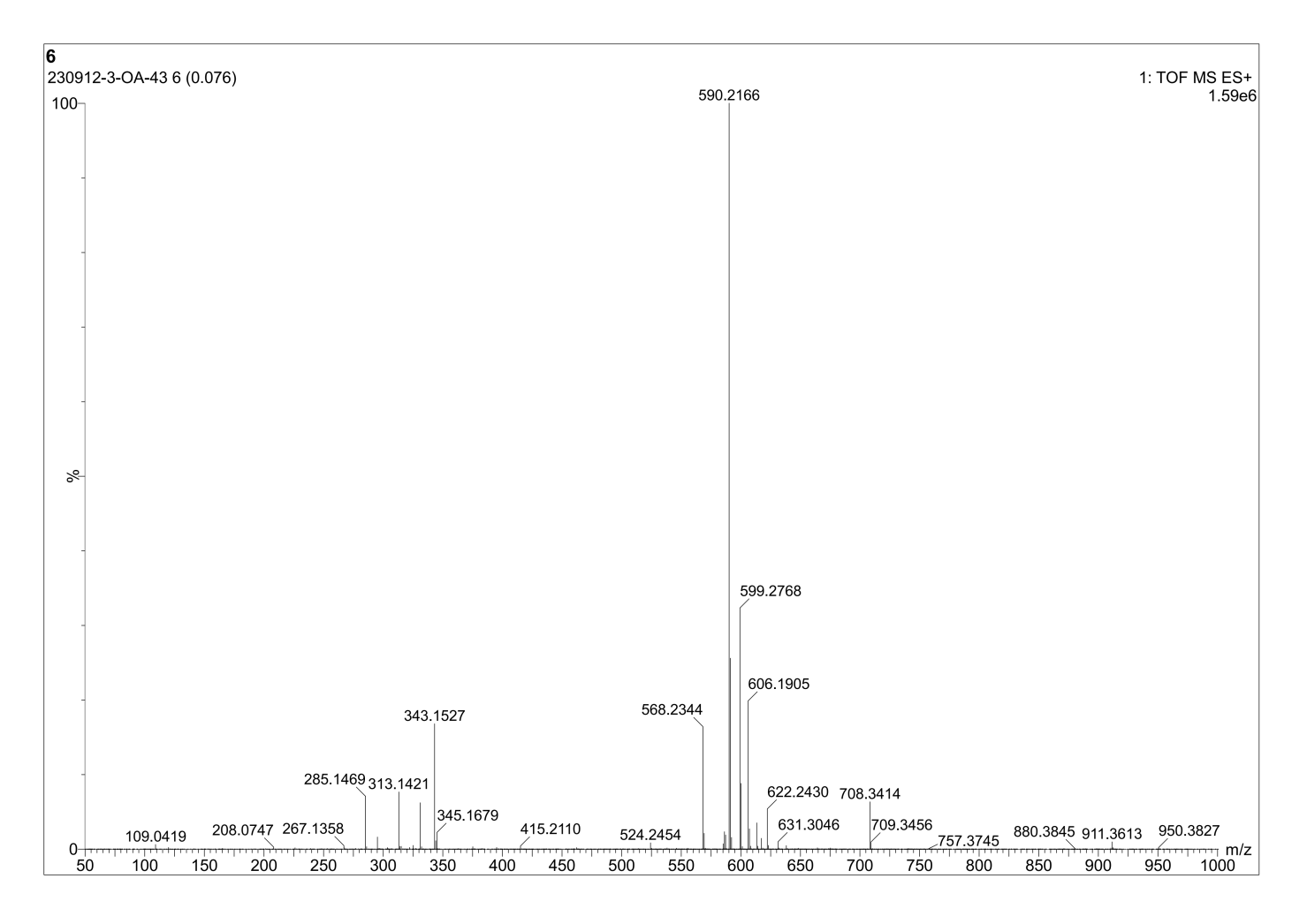

^1^H NMR, ^13^C NMR and HRMS spectra of compound **7f**.

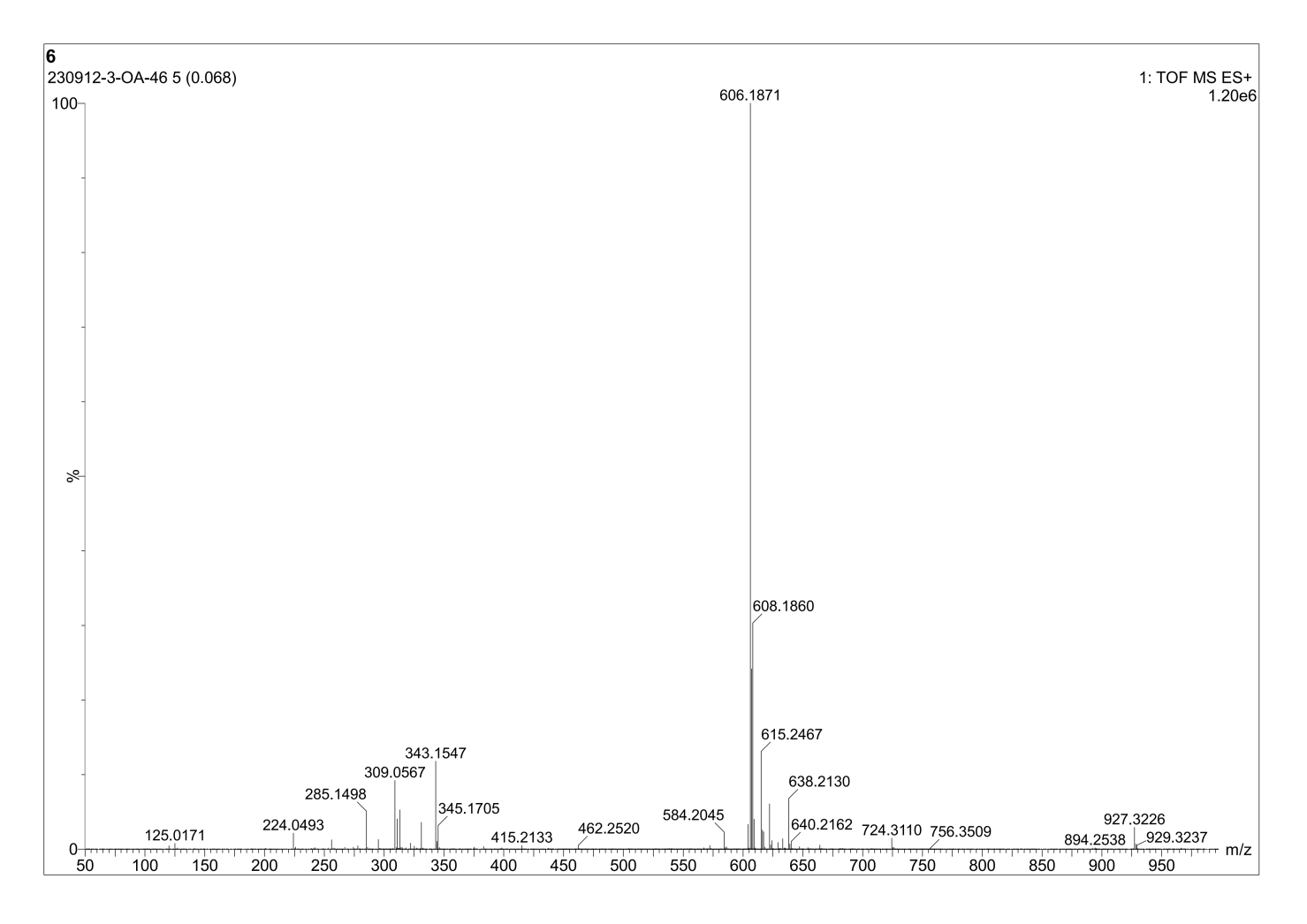

^1^H NMR, ^13^C NMR and HRMS spectra of compound **7g**.

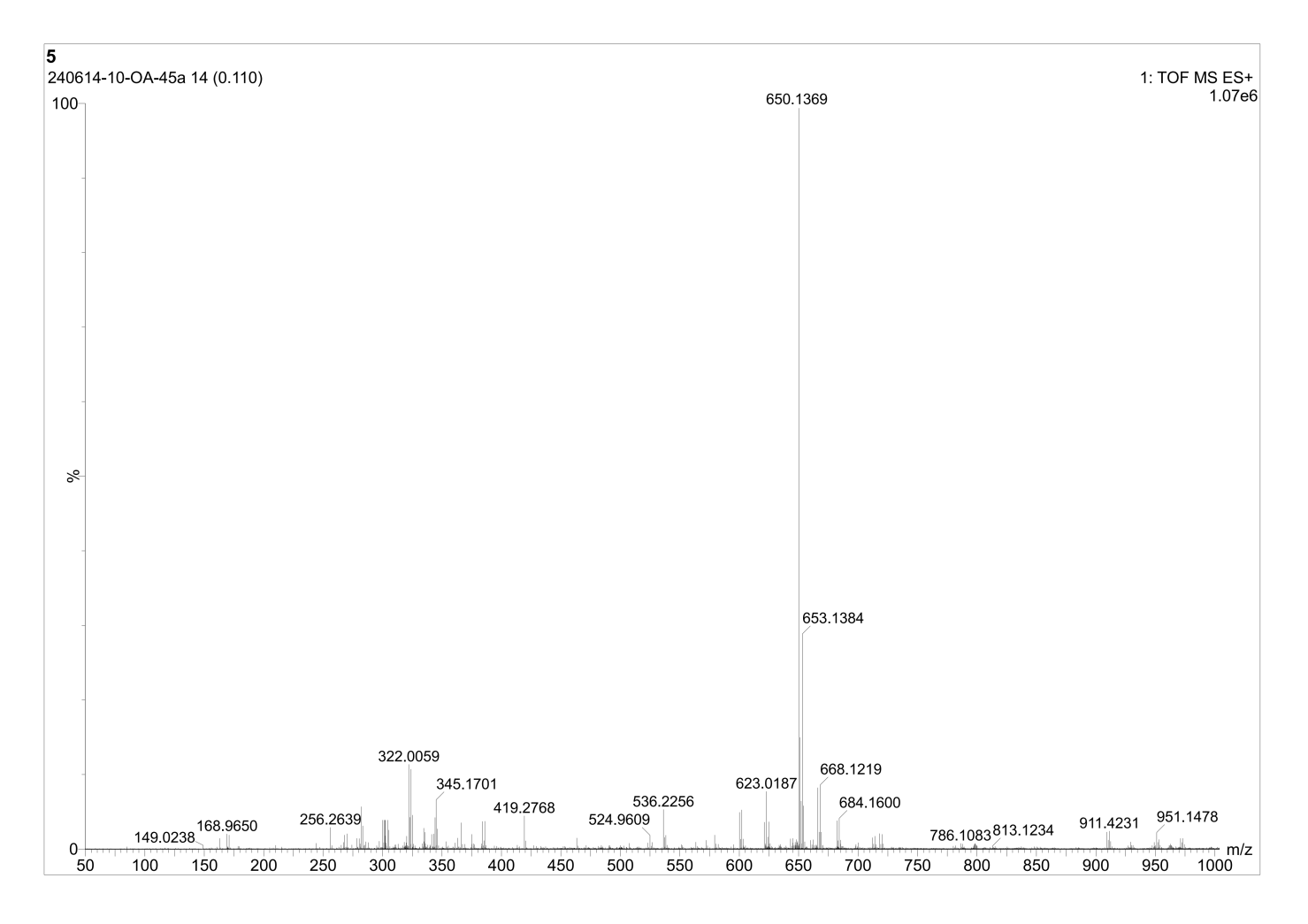

^1^H NMR, ^13^C NMR and HRMS spectra of compound **7h**.

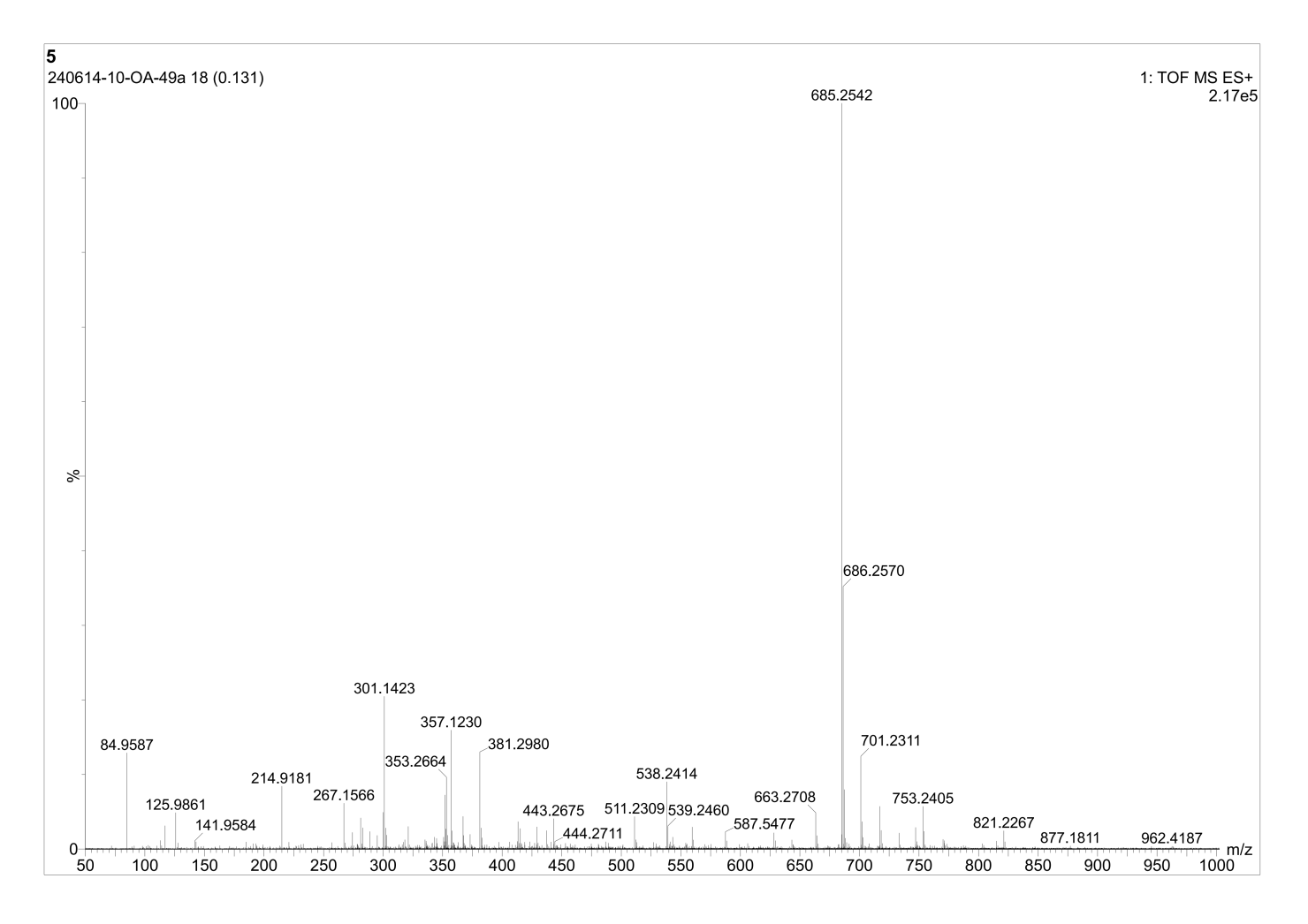

The potential targets of compound **7h**.

Table S1. The potential targets of compound **7h** predicted by Pharmmapper.

| **PDB ID** | **Gene Name** | **Z-score** | **PDB ID** | **Gene Name** | **Z-score** | **PDB ID** | **Gene Name** | **Z-score** |
| --- | --- | --- | --- | --- | --- | --- | --- | --- |
| 2yxj | BCL2L1 | 4.39512 | 3dy6 | PPARD | 1.27487 | 1csb | CTSB | 0.904664 |
| 2gqg | ABL1 | 2.78193 | 1xap | RARB | 1.26757 | 1s9j | MAP2K1 | 0.90373 |
| 1t48 | PTPN1 | 2.49087 | 1y2c | PDE4D | 1.2526 | 1uhl | RXRB | 0.899168 |
| 1ln3 | PCTP | 2.24935 | 2rfn | MET | 1.24418 | 1uhl | NR1H3 | 0.899168 |
| 2iit | DPP4 | 2.23472 | 1utt | MMP12 | 1.23196 | 1uhl | NCOA2 | 0.899168 |
| 1dkf | RARA | 2.14137 | 2i6b | ADK | 1.21877 | 1gbn | OAT | 0.890504 |
| 1o1v | FABP6 | 2.03212 | 2g22 | REN | 1.20961 | 2hmb | FABP3 | 0.890431 |
| 1gzr | IGF1 | 2.00788 | 1jqe | HNMT | 1.2015 | 1sz7 | TRAPPC3 | 0.883707 |
| 1og5 | CYP2C9 | 1.8763 | 1xcw | AMY2A | 1.19247 | 2f57 | PAK5 | 0.873886 |
| 1vjy | TGFBR1 | 1.86443 | 3f0r | HDAC8 | 1.18528 | 1tfg | TGFB2 | 0.869217 |
| 1so2 | PDE3B | 1.80149 | 3b92 | ADAM17 | 1.1767 | 1osh | NR1HH4 | 0.859923 |
| 2p4i | TEK | 1.76517 | 1nmx | DTYMK | 1.14209 | 1h1b | ELANE | 0.856009 |
| 1s8c | HMOX1 | 1.67914 | 1hrk | FECH | 1.13118 | 1vj5 | EPHX2 | 0.845727 |
| 1x0n | GRB2 | 1.65657 | 1nuh | GPI | 1.13107 | 1r7t | ABO | 0.790603 |
| 1m48 | IL2 | 1.62822 | 1h9u | RXRB | 1.12558 | 1u59 | ZAP70 | 0.768188 |
| 1onq | CD1A | 1.58118 | 1i7g | PPARN | 1.12521 | 1nhz | NR3C1 | 0.766774 |
| 1onq | B2M | 1.58118 | 1he3 | BLVRB | 1.08771 | 2q6c | HMGCR | 0.758033 |
| 2b7a | JAK2 | 1.57355 | 1ma0 | ADH5 | 1.07562 | 1itu | DPEP1 | 0.740493 |
| 1t4e | MDM2 | 1.54898 | 1o6u | SCE14L2 | 1.05589 | 1tjj | GM2A | 0.688326 |
| 2vd1 | HPGDS | 1.54679 | 1hov | MMP2 | 1.04684 | 1n69 | PSAP | 0.669805 |
| 1w6j | LSS | 1.525 | 1xvp | RXRA | 1.04161 | 1wda | PADI4 | 0.657948 |
| 1ro6 | PDE4B | 1.52402 | 1xvp | NR1I3 | 1.04161 | 1v4s | GCK | 0.65552 |
| 2j95 | F10 | 1.52113 | 1xvp | NCOA1 | 1.04161 | 2h8h | SRC | 0.623195 |
| 1qab | RBP4 | 1.4992 | 1yk7 | CYSK | 1.02517 | 1iz2 | SERPINA1 | 0.606212 |
| 1qab | TTR | 1.4992 | 1jk7 | PPP1CC | 1.01336 | 1zpb | F11 | 0.586391 |
| 1fd0 | RARG | 1.46688 | 1oiz | TTPA | 1.0063 | 1pq2 | CYP2C8 | 0.580305 |
| 3czr | HSD11B1 | 1.43732 | 1ctr | CALM1 | 1.00541 | 1uzf | ACE | 0.56911 |
| 2pd9 | AKR1B1 | 1.41788 | 1s0z | VDR | 1.00512 | 1pl6 | SORD | 0.561344 |
| 2bu5 | PDK2 | 1.38424 | 1dhf | DHFR | 0.999632 | 1wok | PARP1 | 0.552835 |
| 1oec | FGFR2 | 1.38346 | 1xbc | SYK | 0.987186 | 1nav | THRA | 0.551703 |
| 1yvj | JAK3 | 1.32913 | 2fky | KIF11 | 0.979908 | 3bbt | ERBB4 | 0.524974 |
| 1p62 | DCK | 1.31738 | 2o9i | NR1I2 | 0.971677 | 3d7t | CSK | 0.524566 |
| 1lv2 | HNF4G | 1.31631 | 1yvl | STAT1 | 0.971655 | 1snu | ITK | 0.519907 |
| 1x89 | LCN2 | 1.29011 | 1l7x | PYGL | 0.931722 | 1lt8 | BHMT | 0.504461 |
| 1zyj | MAOK14 | 1.28624 | 1xjd | PRKCQ | 0.931483 |  |  |  |
| 1m6d | CTSF | 1.27558 | 2rl5 | KDR | 0.927408 |  |  |  |

The GO enrichment analysis of compound **7h**.

Table S2. The potential biological processes (BP) of compound **7h**.

| **GO ID** | **Biological Process** | **P-value** | **Count** | **Enrichment** |
| --- | --- | --- | --- | --- |
| GO:0009755 | hormone-mediated signaling pathway | 3.74E-11 | 9 | 41.26285714 |
| GO:0018108 | peptidyl-tyrosine phosphorylation | 1.65E-10 | 9 | 34.6608 |
| GO:0016310 | phosphorylation | 2.59E-09 | 19 | 5.872616372 |
| GO:0042789 | mRNA transcription from RNA polymerase II promoter | 5.51E-09 | 8 | 31.43836735 |
| GO:0048384 | retinoic acid receptor signaling pathway | 2.64E-08 | 6 | 64.18666667 |
| GO:0030154 | cell differentiation | 4.54E-08 | 18 | 5.212150376 |
| GO:0045944 | positive regulation of transcription from RNA polymerase II promoter | 4.78E-08 | 24 | 3.757268293 |
| GO:0006468 | protein phosphorylation | 7.27E-08 | 14 | 7.113034301 |
| GO:0032526 | response to retinoic acid | 1.10E-07 | 7 | 29.95377778 |
| GO:0000122 | negative regulation of transcription from RNA polymerase II promoter | 3.51E-06 | 19 | 3.618832839 |
| GO:0007165 | signal transduction | 4.76E-06 | 21 | 3.232422062 |
| GO:0051897 | positive regulation of protein kinase B signaling | 5.27E-06 | 9 | 9.317419355 |
| GO:1904707 | positive regulation of vascular smooth muscle cell proliferation | 7.66E-06 | 6 | 21.79924528 |
| GO:0008284 | positive regulation of cell proliferation | 1.06E-05 | 13 | 4.966825397 |
| GO:0007169 | transmembrane receptor protein tyrosine kinase signaling pathway | 2.32E-05 | 7 | 12.14342342 |
| GO:0030522 | intracellular receptor signaling pathway | 2.71E-05 | 5 | 28.31764706 |
| GO:0032570 | response to progesterone | 2.71E-05 | 5 | 28.31764706 |
| GO:0007275 | multicellular organism development | 5.41E-05 | 6 | 14.62481013 |
| GO:0032496 | response to lipopolysaccharide | 8.20E-05 | 7 | 9.697266187 |
| GO:0070374 | positive regulation of ERK1 and ERK2 cascade | 1.58E-04 | 8 | 6.877142857 |

Table S3. The potential cellular composition (CC) of compound **7h**.

| **GO ID** | **Cellular Composition** | **P-value** | **Count** | **Enrichment** |
| --- | --- | --- | --- | --- |
| GO:0005829 | cytosol | 2.16E-12 | 61 | 2.260756727 |
| GO:0090575 | RNA polymerase II transcription factor complex | 1.63E-07 | 9 | 14.77512 |
| GO:0005576 | extracellular region | 8.11E-07 | 29 | 2.739912523 |
| GO:0005615 | extracellular space | 3.49E-06 | 26 | 2.763055412 |
| GO:0070062 | extracellular exosome | 4.96E-06 | 28 | 2.563980366 |
| GO:0045121 | membrane raft | 7.84E-06 | 9 | 8.836794258 |
| GO:0005654 | nucleoplasm | 8.33E-06 | 39 | 2.014901813 |
| GO:0043235 | receptor complex | 8.40E-06 | 9 | 8.753033175 |
| GO:0032991 | macromolecular complex | 2.80E-05 | 14 | 4.151647399 |
| GO:0031234 | extrinsic component of cytoplasmic side of plasma membrane | 2.98E-05 | 5 | 27.73108108 |
| GO:0000785 | chromatin | 6.83E-05 | 17 | 3.180100273 |
| GO:0005739 | mitochondrion | 1.47E-04 | 19 | 2.732298528 |
| GO:0005667 | transcription factor complex | 7.62E-04 | 7 | 6.4128125 |
| GO:0005759 | mitochondrial matrix | 8.33E-04 | 9 | 4.504609756 |
| GO:0005911 | cell-cell junction | 0.002133664 | 6 | 6.584278075 |
| GO:0005737 | cytoplasm | 0.003569013 | 41 | 1.49283357 |
| GO:0005925 | focal adhesion | 0.004696838 | 8 | 3.835700935 |
| GO:0009925 | basal plasma membrane | 0.004718432 | 4 | 11.72628571 |
| GO:0005886 | plasma membrane | 0.005512152 | 39 | 1.483445783 |
| GO:0005634 | nucleus | 0.006769579 | 42 | 1.43145989 |

Table S4. The potential molecular functions (MF) of compound **7h**.

| **GO ID** | **Molecular Function** | **P-value** | **Count** | **Enrichment** |
| --- | --- | --- | --- | --- |
| GO:0004879 | RNA polymerase II transcription factor activity, ligand-activated sequence-specific DNA binding | 6.11E-17 | 13 | 45.45907407 |
| GO:0004713 | protein tyrosine kinase activity | 1.74E-11 | 12 | 20.05274336 |
| GO:0008270 | zinc ion binding | 8.92E-10 | 23 | 4.885365579 |
| GO:0004715 | non-membrane spanning protein tyrosine kinase activity | 3.98E-09 | 8 | 32.84 |
| GO:0043565 | sequence-specific DNA binding | 2.07E-08 | 14 | 7.91502994 |
| GO:0042802 | identical protein binding | 6.24E-07 | 27 | 2.940259516 |
| GO:0005524 | ATP binding | 3.50E-06 | 24 | 2.942805195 |
| GO:0042803 | protein homodimerization activity | 7.36E-06 | 16 | 4.060860215 |
| GO:1901363 | heterocyclic compound binding | 4.88E-05 | 4 | 53.95142857 |
| GO:0004714 | transmembrane receptor protein tyrosine kinase activity | 6.83E-05 | 5 | 22.4797619 |
| GO:0016922 | ligand-dependent nuclear receptor binding | 7.50E-05 | 5 | 21.95697674 |
| GO:0003700 | transcription factor activity, sequence-specific DNA binding | 8.13E-05 | 13 | 4.030853859 |
| GO:0046965 | retinoid X receptor binding | 1.08E-04 | 4 | 41.96222222 |
| GO:0004672 | protein kinase activity | 1.91E-04 | 10 | 4.904675325 |
| GO:0044323 | retinoic acid-responsive element binding | 4.03E-04 | 3 | 94.415 |
| GO:0046875 | ephrin receptor binding | 4.16E-04 | 4 | 26.97571429 |
| GO:0097110 | scaffold protein binding | 5.00E-04 | 5 | 13.48785714 |
| GO:0004175 | endopeptidase activity | 5.86E-04 | 5 | 12.93356164 |
| GO:0019899 | enzyme binding | 7.85E-04 | 9 | 4.544037433 |
| GO:0005102 | receptor binding | 8.70E-04 | 9 | 4.472289474 |

The KEGG enrichment analysis of compound **7h**.

Table S5. The potential pathways of compound **7h**.

| **KEGG ID** | **Pathway** | **P-value** | **Count** | **Enrichment** |
| --- | --- | --- | --- | --- |
| hsa05200 | Pathways in cancer | 1.80E-08 | 23 | 4.078154426 |
| hsa01521 | EGFR tyrosine kinase inhibitor resistance | 1.54E-06 | 9 | 10.72619703 |
| hsa04659 | Th17 cell differentiation | 1.74E-06 | 10 | 8.717793881 |
| hsa04151 | PI3K-Akt signaling pathway | 2.00E-05 | 15 | 3.933934843 |
| hsa04014 | Ras signaling pathway | 3.37E-05 | 12 | 4.787398674 |
| hsa04919 | Thyroid hormone signaling pathway | 3.71E-05 | 9 | 7.003054258 |
| hsa05205 | Proteoglycans in cancer | 5.36E-05 | 11 | 5.052067869 |
| hsa05223 | Non-small cell lung cancer | 1.02E-04 | 7 | 9.153683575 |
| hsa05220 | Chronic myeloid leukemia | 1.38E-04 | 7 | 8.671910755 |
| hsa05226 | Gastric cancer | 1.62E-04 | 9 | 5.687044062 |
| hsa04910 | Insulin signaling pathway | 5.69E-04 | 8 | 5.497937163 |
| hsa04915 | Estrogen signaling pathway | 5.69E-04 | 8 | 5.497937163 |
| hsa04917 | Prolactin signaling pathway | 8.10E-04 | 6 | 8.070186335 |
| hsa03320 | PPAR signaling pathway | 0.001108458 | 6 | 7.532173913 |
| hsa05161 | Hepatitis B | 0.001525576 | 8 | 4.64949007 |
| hsa04660 | T cell receptor signaling pathway | 0.001663198 | 7 | 5.446819978 |
| hsa05207 | Chemical carcinogenesis - receptor activation | 0.001679834 | 9 | 3.997026251 |
| hsa04012 | ErbB signaling pathway | 0.001940505 | 6 | 6.646035806 |
| hsa04658 | Th1 and Th2 cell differentiation | 0.002746957 | 6 | 6.140359168 |
| hsa01522 | Endocrine resistance | 0.00361147 | 6 | 5.764418811 |

The single-crystal data and absolute configuration of compound **4 (240423_WJF_1_0m).**

| **Table SC-1 Crystal data and structure refinement for compound 4 (240423_WJF_1_0m).** | |
| --- | --- |
| Identification code | 240423_WJF_1_0m |
| Empirical formula | C_20_H_24_O_6_ |
| Formula weight | 360.39 |
| Temperature/K | 170.00 |
| Crystal system | orthorhombic |
| Space group | P2_1_2_1_2_1_ |
| a/Å | 6.4862(3) |
| b/Å | 14.5322(6) |
| c/Å | 18.0306(8) |
| α/° | 90 |
| β/° | 90 |
| γ/° | 90 |
| Volume/Å^3^ | 1699.54(13) |
| Z | 4 |
| ρ_calc_g/cm^3^ | 1.408 |
| μ/mm^‑1^ | 0.545 |
| F(000) | 768.0 |
| Crystal size/mm^3^ | 0.07 × 0.05 × 0.03 |
| Radiation | GaKα (λ = 1.34139) |
| 2Θ range for data collection/° | 6.796 to 121.148 |
| Index ranges | -8 ≤ h ≤ 8, -18 ≤ k ≤ 17, -23 ≤ l ≤ 23 |
| Reflections collected | 33916 |
| Independent reflections | 3911 [R_int_ = 0.0356, R_sigma_ = 0.0205] |
| Data/restraints/parameters | 3911/0/238 |
| Goodness-of-fit on F^2^ | 1.031 |
| Final R indexes [I>=2σ (I)] | R_1_ = 0.0327, wR_2_ = 0.0864 |
| Final R indexes [all data] | R_1_ = 0.0342, wR_2_ = 0.0875 |
| Largest diff. peak/hole / e Å^-3^ | 0.18/-0.20 |
| Flack parameter | 0.10(5) |

| Table SC-2 Fractional Atomic Coordinates (×10^4^) and Equivalent Isotropic Displacement Parameters (Å^2^×10^3^) for **compound 4**. U_eq_ is defined as 1/3 of the trace of the orthogonalised U_IJ_ tensor. | | | | |
| --- | --- | --- | --- | --- |
| **Atom** | ***x*** | ***y*** | ***z*** | **U(eq)** |
| O1 | 1529(3) | 1749.4(10) | 7437.8(9) | 47.9(4) |
| C1 | 3424(3) | 3058.5(12) | 7928.8(10) | 30.3(4) |
| O2 | -208(2) | 3030.3(10) | 7893.7(9) | 43.2(4) |
| C2 | 4134(3) | 3412.5(15) | 8710.1(11) | 35.3(4) |
| O3 | 355(2) | 5620.1(10) | 6124.9(9) | 42.4(4) |
| C3 | 5487(3) | 4269.2(15) | 8606.1(11) | 37.5(4) |
| O4 | 2421(2) | 5417.7(8) | 7099.4(7) | 32.0(3) |
| C4 | 4393(3) | 5046.3(14) | 8192.3(11) | 34.7(4) |
| O5 | 5676.9(19) | 4699.4(9) | 7005.7(7) | 29.6(3) |
| C5 | 3820(3) | 4728.2(12) | 7425.6(10) | 27.7(4) |
| O6 | 1470(2) | 4125.6(11) | 4878.8(8) | 40.7(3) |
| C6 | 2673(3) | 3808.0(11) | 7380.5(10) | 25.8(3) |
| C7 | 412(3) | 3914.8(13) | 7628.8(11) | 32.0(4) |
| C8 | 1518(3) | 2439.2(13) | 7988.8(11) | 37.4(4) |
| C9 | 2379(4) | 3637.4(16) | 9258.1(12) | 43.7(5) |
| C10 | 5459(4) | 2650.8(18) | 9058.1(13) | 50.7(6) |
| C11 | 2678(3) | 3509.9(11) | 6544.4(10) | 25.9(3) |
| C12 | 3018(3) | 4422.6(11) | 6093.8(10) | 26.6(4) |
| C13 | 1725(3) | 5192.0(12) | 6404.3(11) | 30.9(4) |
| C14 | 4214(3) | 2777.1(12) | 6264.3(10) | 29.7(4) |
| C15 | 6440(3) | 3096.9(13) | 6149.1(11) | 32.7(4) |
| C16 | 6489(3) | 4034.4(13) | 5758.2(10) | 31.4(4) |
| C17 | 5194(3) | 4030.9(13) | 5055.4(11) | 32.6(4) |
| C18 | 2981(3) | 4195.8(12) | 5268.0(10) | 30.6(4) |
| C19 | 5264(3) | 4719.6(12) | 6228.8(10) | 28.1(4) |
| C20 | 5787(4) | 3957.0(15) | 4358.6(12) | 43.1(5) |

| Table SC-3 Anisotropic Displacement Parameters (Å^2^×10^3^) for **compound 4**. The Anisotropic displacement factor exponent takes the form: -2π^2^[h^2^a*^2^U_11_+2hka*b*U_12_+…]. | | | | | | |
| --- | --- | --- | --- | --- | --- | --- |
| **Atom** | **U_11_** | **U_22_** | **U_33_** | **U_23_** | **U_13_** | **U_12_** |
| O1 | 64.0(11) | 32.0(7) | 47.6(9) | -3.6(6) | 15.7(8) | -18.5(7) |
| C1 | 34.3(9) | 27.9(8) | 28.7(8) | 2.0(7) | 5.4(8) | -0.4(7) |
| O2 | 34.9(7) | 38.5(8) | 56.2(9) | 5.5(7) | 13.4(7) | -10.2(6) |
| C2 | 39.8(10) | 37.8(10) | 28.2(8) | 2.4(7) | 2.2(8) | -1.6(8) |
| O3 | 38.9(8) | 38.4(7) | 49.8(8) | 3.0(6) | 4.3(7) | 15.7(6) |
| C3 | 36.8(10) | 44.7(11) | 31.1(9) | -2.8(8) | -0.1(8) | -5.8(9) |
| O4 | 38.4(7) | 22.8(6) | 34.8(6) | -1.5(5) | 8.4(6) | 4.3(5) |
| C4 | 39.1(10) | 32.7(9) | 32.3(9) | -5.3(7) | 6.1(8) | -7.4(8) |
| O5 | 25.8(6) | 31.4(6) | 31.7(6) | -1.8(5) | 5.8(5) | -5.2(5) |
| C5 | 27.8(8) | 24.1(8) | 31.1(8) | -1.9(7) | 6.1(7) | -1.0(6) |
| O6 | 38.8(7) | 49.9(8) | 33.5(7) | 1.9(6) | -1.2(6) | 3.5(7) |
| C6 | 25.7(8) | 22.7(7) | 29.1(8) | -0.1(6) | 6.6(7) | -2.2(6) |
| C7 | 26.8(8) | 33.2(9) | 36.0(9) | -0.5(7) | 7.9(7) | -3.5(7) |
| C8 | 45.3(11) | 29.6(9) | 37.2(10) | 2.9(7) | 10.1(9) | -8.2(8) |
| C9 | 54.3(13) | 47.0(12) | 29.8(9) | 0.4(8) | 10.6(9) | -7.6(10) |
| C10 | 60.3(15) | 51.7(13) | 40.0(11) | 10.2(10) | -5.0(11) | 6.5(11) |
| C11 | 24.5(8) | 22.6(7) | 30.5(8) | 1.1(6) | 3.0(7) | -2.8(6) |
| C12 | 27.1(8) | 22.9(7) | 29.8(8) | 1.2(6) | 5.6(7) | 2.1(6) |
| C13 | 31.6(9) | 25.9(8) | 35.2(9) | 2.3(7) | 9.3(8) | 2.5(7) |
| C14 | 35.4(9) | 21.6(8) | 32.0(8) | -1.3(6) | 3.0(8) | 0.5(7) |
| C15 | 30.6(9) | 29.9(9) | 37.7(10) | -3.4(7) | 3.0(8) | 8.4(7) |
| C16 | 26.5(8) | 31.8(9) | 36.0(9) | -1.4(7) | 8.9(7) | 0.6(7) |
| C17 | 35.0(9) | 27.0(8) | 35.7(9) | -1.1(7) | 7.2(8) | 3.7(7) |
| C18 | 34.8(10) | 27.7(8) | 29.4(9) | 2.9(7) | 4.0(7) | 3.4(7) |
| C19 | 28.9(8) | 23.7(8) | 31.6(8) | 0.3(6) | 6.8(7) | -2.7(7) |
| C20 | 52.7(13) | 39.0(11) | 37.5(10) | 3.2(8) | 11.7(9) | 13.1(10) |

| Table SC-4 Bond Lengths for **compound 4**. | | | | | | |
| --- | --- | --- | --- | --- | --- | --- |
| **Atom** | **Atom** | **Length/Å** |  | **Atom** | **Atom** | **Length/Å** |
| O1 | C8 | 1.411(3) |  | C5 | C6 | 1.532(2) |
| C1 | C2 | 1.569(3) |  | O6 | C18 | 1.210(2) |
| C1 | C6 | 1.549(2) |  | C6 | C7 | 1.541(2) |
| C1 | C8 | 1.533(3) |  | C6 | C11 | 1.569(2) |
| O2 | C7 | 1.429(2) |  | C11 | C12 | 1.571(2) |
| O2 | C8 | 1.422(3) |  | C11 | C14 | 1.543(2) |
| C2 | C3 | 1.534(3) |  | C12 | C13 | 1.505(2) |
| C2 | C9 | 1.543(3) |  | C12 | C18 | 1.525(2) |
| C2 | C10 | 1.536(3) |  | C12 | C19 | 1.539(2) |
| O3 | C13 | 1.196(2) |  | C14 | C15 | 1.531(3) |
| C3 | C4 | 1.528(3) |  | C15 | C16 | 1.534(3) |
| O4 | C5 | 1.474(2) |  | C16 | C17 | 1.520(3) |
| O4 | C13 | 1.372(2) |  | C16 | C19 | 1.531(2) |
| C4 | C5 | 1.504(3) |  | C17 | C18 | 1.505(3) |
| O5 | C5 | 1.423(2) |  | C17 | C20 | 1.319(3) |
| O5 | C19 | 1.426(2) |  |  |  |  |

| Table SC-5 Bond Angles for **compound 4**. | | | | | | | | |
| --- | --- | --- | --- | --- | --- | --- | --- | --- |
| **Atom** | **Atom** | **Atom** | **Angle/˚** |  | **Atom** | **Atom** | **Atom** | **Angle/˚** |
| C6 | C1 | C2 | 115.76(15) |  | O1 | C8 | O2 | 110.38(19) |
| C8 | C1 | C2 | 111.48(15) |  | O2 | C8 | C1 | 105.77(14) |
| C8 | C1 | C6 | 101.80(15) |  | C6 | C11 | C12 | 105.32(13) |
| C8 | O2 | C7 | 111.23(15) |  | C14 | C11 | C6 | 120.43(15) |
| C3 | C2 | C1 | 108.93(15) |  | C14 | C11 | C12 | 108.84(14) |
| C3 | C2 | C9 | 109.16(17) |  | C13 | C12 | C11 | 110.90(14) |
| C3 | C2 | C10 | 108.35(19) |  | C13 | C12 | C18 | 120.97(15) |
| C9 | C2 | C1 | 115.33(18) |  | C13 | C12 | C19 | 105.08(14) |
| C10 | C2 | C1 | 107.15(17) |  | C18 | C12 | C11 | 108.67(14) |
| C10 | C2 | C9 | 107.70(18) |  | C18 | C12 | C19 | 103.30(14) |
| C4 | C3 | C2 | 113.19(17) |  | C19 | C12 | C11 | 106.73(14) |
| C13 | O4 | C5 | 113.85(13) |  | O3 | C13 | O4 | 120.31(17) |
| C5 | C4 | C3 | 109.65(16) |  | O3 | C13 | C12 | 130.06(18) |
| C5 | O5 | C19 | 111.28(13) |  | O4 | C13 | C12 | 109.51(15) |
| O4 | C5 | C4 | 108.05(15) |  | C15 | C14 | C11 | 116.34(15) |
| O4 | C5 | C6 | 105.86(14) |  | C14 | C15 | C16 | 110.56(15) |
| C4 | C5 | C6 | 115.89(15) |  | C17 | C16 | C15 | 111.62(16) |
| O5 | C5 | O4 | 109.19(14) |  | C17 | C16 | C19 | 100.22(15) |
| O5 | C5 | C4 | 106.79(15) |  | C19 | C16 | C15 | 108.20(14) |
| O5 | C5 | C6 | 110.91(13) |  | C18 | C17 | C16 | 108.32(15) |
| C1 | C6 | C11 | 114.74(14) |  | C20 | C17 | C16 | 129.3(2) |
| C5 | C6 | C1 | 115.29(15) |  | C20 | C17 | C18 | 122.3(2) |
| C5 | C6 | C7 | 111.01(14) |  | O6 | C18 | C12 | 126.69(17) |
| C5 | C6 | C11 | 106.91(13) |  | O6 | C18 | C17 | 127.70(17) |
| C7 | C6 | C1 | 100.63(14) |  | C17 | C18 | C12 | 105.54(15) |
| C7 | C6 | C11 | 108.00(15) |  | O5 | C19 | C12 | 109.10(14) |
| O2 | C7 | C6 | 105.92(15) |  | O5 | C19 | C16 | 115.70(15) |
| O1 | C8 | C1 | 111.30(16) |  | C16 | C19 | C12 | 102.78(14) |

| Table SC-6 Torsion Angles for **compound 4**. | | | | | | | | | | |
| --- | --- | --- | --- | --- | --- | --- | --- | --- | --- | --- |
| **A** | **B** | **C** | **D** | **Angle/˚** |  | **A** | **B** | **C** | **D** | **Angle/˚** |
| C1 | C2 | C3 | C4 | 58.0(2) |  | C8 | O2 | C7 | C6 | 12.6(2) |
| C1 | C6 | C7 | O2 | -31.29(19) |  | C9 | C2 | C3 | C4 | -68.7(2) |
| C1 | C6 | C11 | C12 | -151.38(15) |  | C10 | C2 | C3 | C4 | 174.29(17) |
| C1 | C6 | C11 | C14 | -28.1(2) |  | C11 | C6 | C7 | O2 | 89.29(17) |
| C2 | C1 | C6 | C5 | 35.6(2) |  | C11 | C12 | C13 | O3 | -116.9(2) |
| C2 | C1 | C6 | C7 | -83.87(19) |  | C11 | C12 | C13 | O4 | 67.04(19) |
| C2 | C1 | C6 | C11 | 160.49(16) |  | C11 | C12 | C18 | O6 | 84.3(2) |
| C2 | C1 | C8 | O1 | -147.40(17) |  | C11 | C12 | C18 | C17 | -92.84(16) |
| C2 | C1 | C8 | O2 | 92.69(19) |  | C11 | C12 | C19 | O5 | -49.05(17) |
| C2 | C3 | C4 | C5 | -61.7(2) |  | C11 | C12 | C19 | C16 | 74.25(16) |
| C3 | C4 | C5 | O4 | 168.89(15) |  | C11 | C14 | C15 | C16 | -43.9(2) |
| C3 | C4 | C5 | O5 | -73.76(18) |  | C12 | C11 | C14 | C15 | 44.8(2) |
| C3 | C4 | C5 | C6 | 50.4(2) |  | C13 | O4 | C5 | C4 | -177.93(14) |
| O4 | C5 | C6 | C1 | -158.46(14) |  | C13 | O4 | C5 | O5 | 66.28(18) |
| O4 | C5 | C6 | C7 | -44.88(18) |  | C13 | O4 | C5 | C6 | -53.18(18) |
| O4 | C5 | C6 | C11 | 72.68(16) |  | C13 | C12 | C18 | O6 | -45.7(3) |
| C4 | C5 | C6 | C1 | -38.7(2) |  | C13 | C12 | C18 | C17 | 137.22(17) |
| C4 | C5 | C6 | C7 | 74.9(2) |  | C13 | C12 | C19 | O5 | 68.77(17) |
| C4 | C5 | C6 | C11 | -167.58(16) |  | C13 | C12 | C19 | C16 | -167.93(14) |
| O5 | C5 | C6 | C1 | 83.23(18) |  | C14 | C11 | C12 | C13 | -173.52(15) |
| O5 | C5 | C6 | C7 | -163.20(15) |  | C14 | C11 | C12 | C18 | 51.21(19) |
| O5 | C5 | C6 | C11 | -45.63(19) |  | C14 | C11 | C12 | C19 | -59.59(17) |
| C5 | O4 | C13 | O3 | 168.89(17) |  | C14 | C15 | C16 | C17 | -51.8(2) |
| C5 | O4 | C13 | C12 | -14.6(2) |  | C14 | C15 | C16 | C19 | 57.6(2) |
| C5 | O5 | C19 | C12 | -19.97(18) |  | C15 | C16 | C17 | C18 | 82.77(18) |
| C5 | O5 | C19 | C16 | -135.21(15) |  | C15 | C16 | C17 | C20 | -101.1(2) |
| C5 | C6 | C7 | O2 | -153.81(15) |  | C15 | C16 | C19 | O5 | 45.4(2) |
| C5 | C6 | C11 | C12 | -22.20(17) |  | C15 | C16 | C19 | C12 | -73.38(18) |
| C5 | C6 | C11 | C14 | 101.11(17) |  | C16 | C17 | C18 | O6 | -169.82(19) |
| C6 | C1 | C2 | C3 | -44.3(2) |  | C16 | C17 | C18 | C12 | 7.24(19) |
| C6 | C1 | C2 | C9 | 78.8(2) |  | C17 | C16 | C19 | O5 | 162.37(15) |
| C6 | C1 | C2 | C10 | -161.31(18) |  | C17 | C16 | C19 | C12 | 43.58(16) |
| C6 | C1 | C8 | O1 | 88.58(18) |  | C18 | C12 | C13 | O3 | 12.1(3) |
| C6 | C1 | C8 | O2 | -31.32(18) |  | C18 | C12 | C13 | O4 | -164.00(15) |
| C6 | C11 | C12 | C13 | -43.10(19) |  | C18 | C12 | C19 | O5 | -163.54(13) |
| C6 | C11 | C12 | C18 | -178.38(14) |  | C18 | C12 | C19 | C16 | -40.24(17) |
| C6 | C11 | C12 | C19 | 70.82(16) |  | C19 | O5 | C5 | O4 | -43.30(18) |
| C6 | C11 | C14 | C15 | -76.8(2) |  | C19 | O5 | C5 | C4 | -159.89(14) |
| C7 | O2 | C8 | O1 | -108.42(18) |  | C19 | O5 | C5 | C6 | 72.99(18) |
| C7 | O2 | C8 | C1 | 12.1(2) |  | C19 | C12 | C13 | O3 | 128.2(2) |
| C7 | C6 | C11 | C12 | 97.33(16) |  | C19 | C12 | C13 | O4 | -47.92(18) |
| C7 | C6 | C11 | C14 | -139.36(16) |  | C19 | C12 | C18 | O6 | -162.66(19) |
| C8 | C1 | C2 | C3 | -160.02(17) |  | C19 | C12 | C18 | C17 | 20.24(17) |
| C8 | C1 | C2 | C9 | -36.9(2) |  | C19 | C16 | C17 | C18 | -31.61(18) |
| C8 | C1 | C2 | C10 | 83.0(2) |  | C19 | C16 | C17 | C20 | 144.5(2) |
| C8 | C1 | C6 | C5 | 156.68(15) |  | C20 | C17 | C18 | O6 | 13.7(3) |
| C8 | C1 | C6 | C7 | 37.20(17) |  | C20 | C17 | C18 | C12 | -169.24(18) |
| C8 | C1 | C6 | C11 | -78.44(18) |  |  |  |  |  |  |

| Table SC-7 Hydrogen Atom Coordinates (Å×10^4^) and Isotropic Displacement Parameters (Å^2^×10^3^) for **compound 4**. | | | | |
| --- | --- | --- | --- | --- |
| **Atom** | ***x*** | ***y*** | ***z*** | **U(eq)** |
| H1 | 829.44 | 1297.59 | 7582.77 | 72 |
| H1A | 4573.58 | 2703.48 | 7695.21 | 36 |
| H3A | 5922.84 | 4496.6 | 9099.33 | 45 |
| H3B | 6743.33 | 4094.53 | 8328.19 | 45 |
| H4A | 3135.38 | 5229.6 | 8466.28 | 42 |
| H4B | 5312.64 | 5588.32 | 8159.13 | 42 |
| H7A | 297.74 | 4380.43 | 8027.54 | 38 |
| H7B | -463.16 | 4108.45 | 7206.95 | 38 |
| H8 | 1467.57 | 2148.87 | 8491.27 | 45 |
| H9A | 1545.13 | 4143.56 | 9060.51 | 66 |
| H9B | 2968.08 | 3818.98 | 9736.76 | 66 |
| H9C | 1508.22 | 3092.63 | 9325.35 | 66 |
| H10A | 4615.02 | 2099.48 | 9133.67 | 76 |
| H10B | 5998.51 | 2862.22 | 9536.37 | 76 |
| H10C | 6610.39 | 2504.54 | 8726.3 | 76 |
| H11 | 1260.93 | 3283.25 | 6425.02 | 31 |
| H14A | 4223.88 | 2261.53 | 6623.16 | 36 |
| H14B | 3694.43 | 2532.35 | 5787.18 | 36 |
| H15A | 7191.25 | 2638.11 | 5846.15 | 39 |
| H15B | 7141.02 | 3145.21 | 6635.34 | 39 |
| H16 | 7929.3 | 4249.99 | 5663.76 | 38 |
| H19 | 5478.87 | 5357.12 | 6034.8 | 34 |
| H20A | 4804.03 | 4007.87 | 3970.21 | 52 |
| H20B | 7198.2 | 3852.84 | 4244.86 | 52 |


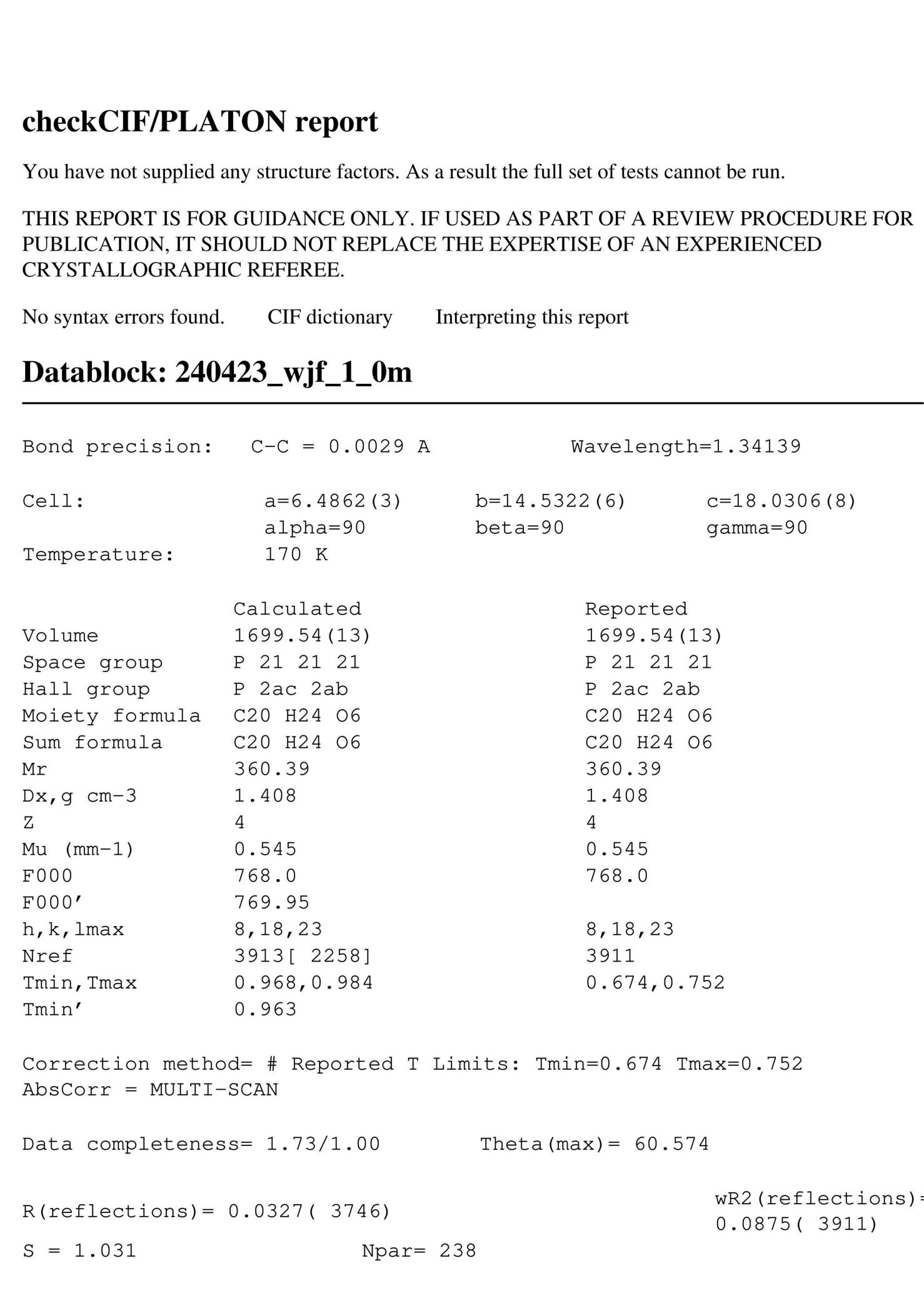


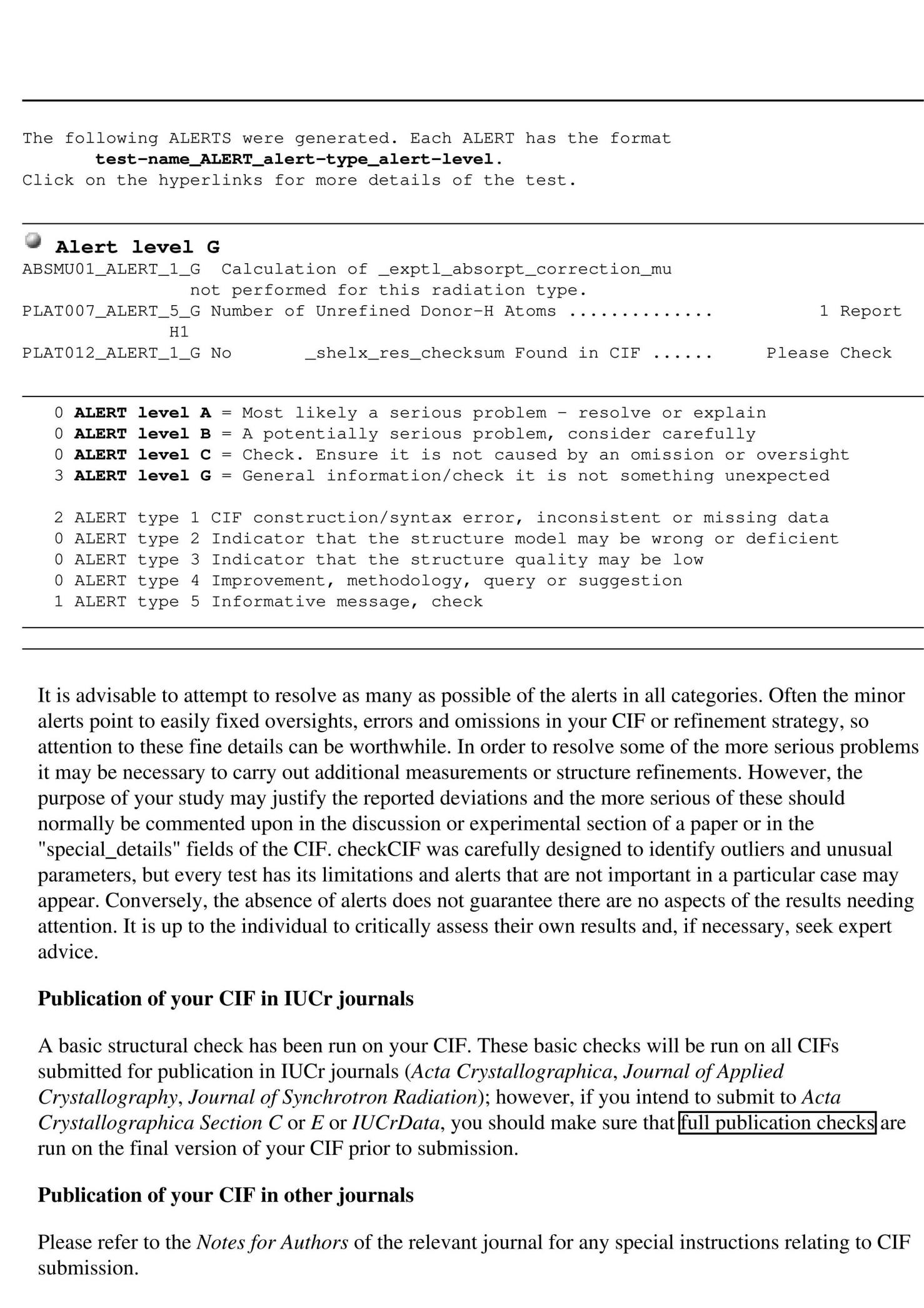


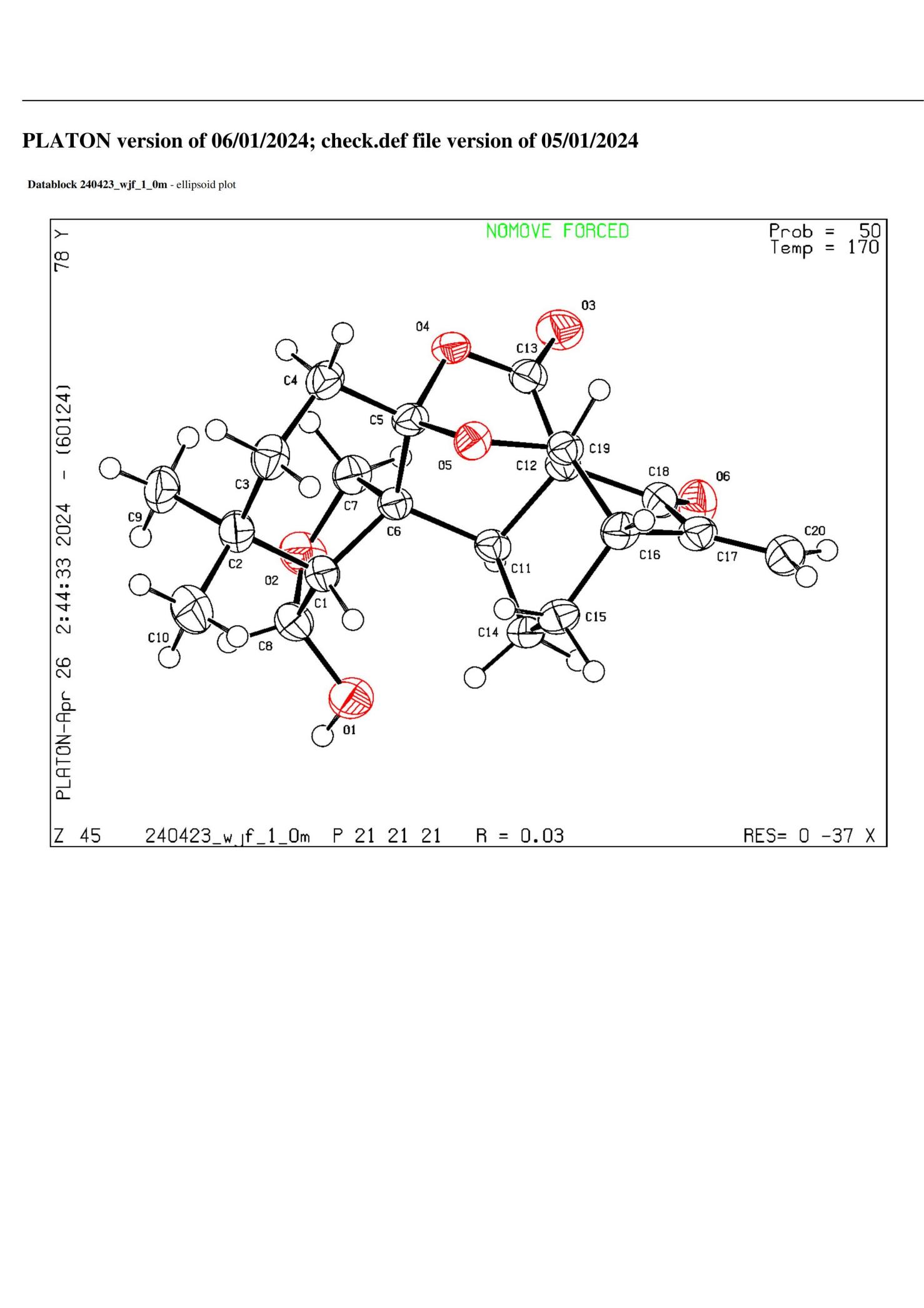


The carbon atoms of compound 4 were renumbered by Ref:


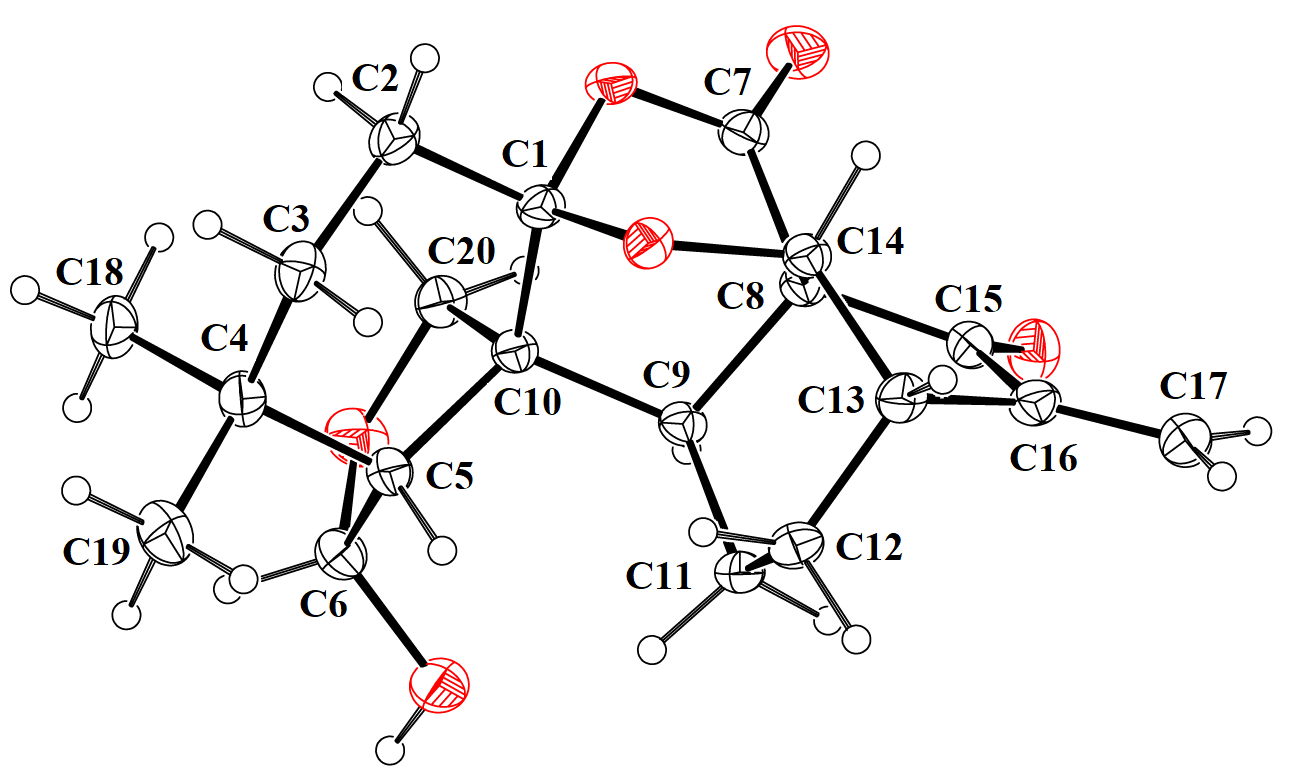

Supplement: Supplementary file 1 [file molecules-29-04066-s001.zip › molecules-3152720-supplementary/Supplemental material/Supplemental material.docx]
