# Supplementary material for: Design, Synthesis, and Anticancer Activity of Novel Enmein-Type Diterpenoid Derivatives Targeting the PI3K/Akt/mTOR Signaling Pathway
Source: Molecules. 2024 Aug 27;29(17):4066. doi: 10.3390/molecules29174066 (PMC11396751; doi:10.3390/molecules29174066)

checkCIF/PLATON report

You have not supplied any structure factors. As a result the full set of tests cannot be run.

THIS REPORT IS FOR GUIDANCE ONLY. IF USED AS PART OF A REVIEW PROCEDURE FOR PUBLICATION, IT SHOULD NOT REPLACE THE EXPERTISE OF AN EXPERIENCED CRYSTALLOGRAPHIC REFEREE.

No syntax errors found. CIF dictionary Interpreting this report

Datablock: 240423_wjf_1_0m

```
Bond precision:   C-C = 0.0029 A           Wavelength=1.34139

Cell:              a=6.4862(3)      b=14.5322(6)      c=18.0306(8)
                   alpha=90         beta=90           gamma=90
Temperature:       170 K

                Calculated                  Reported
Volume          1699.54(13)                 1699.54(13)
Space group     P 21 21 21                  P 21 21 21
Hall group      P 2ac 2ab                   P 2ac 2ab
Moiety formula  C20 H24 O6                  C20 H24 O6
Sum formula     C20 H24 O6                  C20 H24 O6
Mr              360.39                      360.39
Dx,g cm-3       1.408                       1.408
Z               4                           4
Mu (mm-1)       0.545                       0.545
F000            768.0                       768.0
F000'           769.95
h,k,lmax        8,18,23                     8,18,23
Nref            3913[ 2258]                 3911
Tmin,Tmax       0.968,0.984                 0.674,0.752
Tmin'           0.963

Correction method= # Reported T Limits: Tmin=0.674 Tmax=0.752
AbsCorr = MULTI-SCAN

Data completeness= 1.73/1.00       Theta(max)= 60.574

                                                       wR2(reflections)=
R(reflections)= 0.0327( 3746)                          0.0875( 3911)

S = 1.031                  Npar= 238
```

```
The following ALERTS were generated. Each ALERT has the format
       test-name_ALERT_alert-type_alert-level.
Click on the hyperlinks for more details of the test.
```

Alert level G

```
ABSMU01_ALERT_1_G  Calculation of _exptl_absorpt_correction_mu
                not performed for this radiation type.
PLAT007_ALERT_5_G Number of Unrefined Donor-H Atoms .............          1 Report
              H1
PLAT012_ALERT_1_G No       _shelx_res_checksum Found in CIF ......     Please Check
```

```
   0 ALERT level A = Most likely a serious problem - resolve or explain
   0 ALERT level B = A potentially serious problem, consider carefully
   0 ALERT level C = Check. Ensure it is not caused by an omission or oversight
   3 ALERT level G = General information/check it is not something unexpected

   2 ALERT type 1 CIF construction/syntax error, inconsistent or missing data
   0 ALERT type 2 Indicator that the structure model may be wrong or deficient
   0 ALERT type 3 Indicator that the structure quality may be low
   0 ALERT type 4 Improvement, methodology, query or suggestion
   1 ALERT type 5 Informative message, check
```

It is advisable to attempt to resolve as many as possible of the alerts in all categories. Often the minor alerts point to easily fixed oversights, errors and omissions in your CIF or refinement strategy, so attention to these fine details can be worthwhile. In order to resolve some of the more serious problems it may be necessary to carry out additional measurements or structure refinements. However, the purpose of your study may justify the reported deviations and the more serious of these should normally be commented upon in the discussion or experimental section of a paper or in the "special_details" fields of the CIF. checkCIF was carefully designed to identify outliers and unusual parameters, but every test has its limitations and alerts that are not important in a particular case may appear. Conversely, the absence of alerts does not guarantee there are no aspects of the results needing attention. It is up to the individual to critically assess their own results and, if necessary, seek expert advice.

Publication of your CIF in IUCr journals

A basic structural check has been run on your CIF. These basic checks will be run on all CIFs submitted for publication in IUCr journals (*Acta Crystallographica*, *Journal of Applied Crystallography*, *Journal of Synchrotron Radiation*); however, if you intend to submit to *Acta Crystallographica Section C* or *E* or *IUCrData*, you should make sure that full publication checks are run on the final version of your CIF prior to submission.

Publication of your CIF in other journals

Please refer to the *Notes for Authors* of the relevant journal for any special instructions relating to CIF submission.

PLATON version of 06/01/2024; check.def file version of 05/01/2024

Datablock 240423_wjf_1_0m - ellipsoid plot

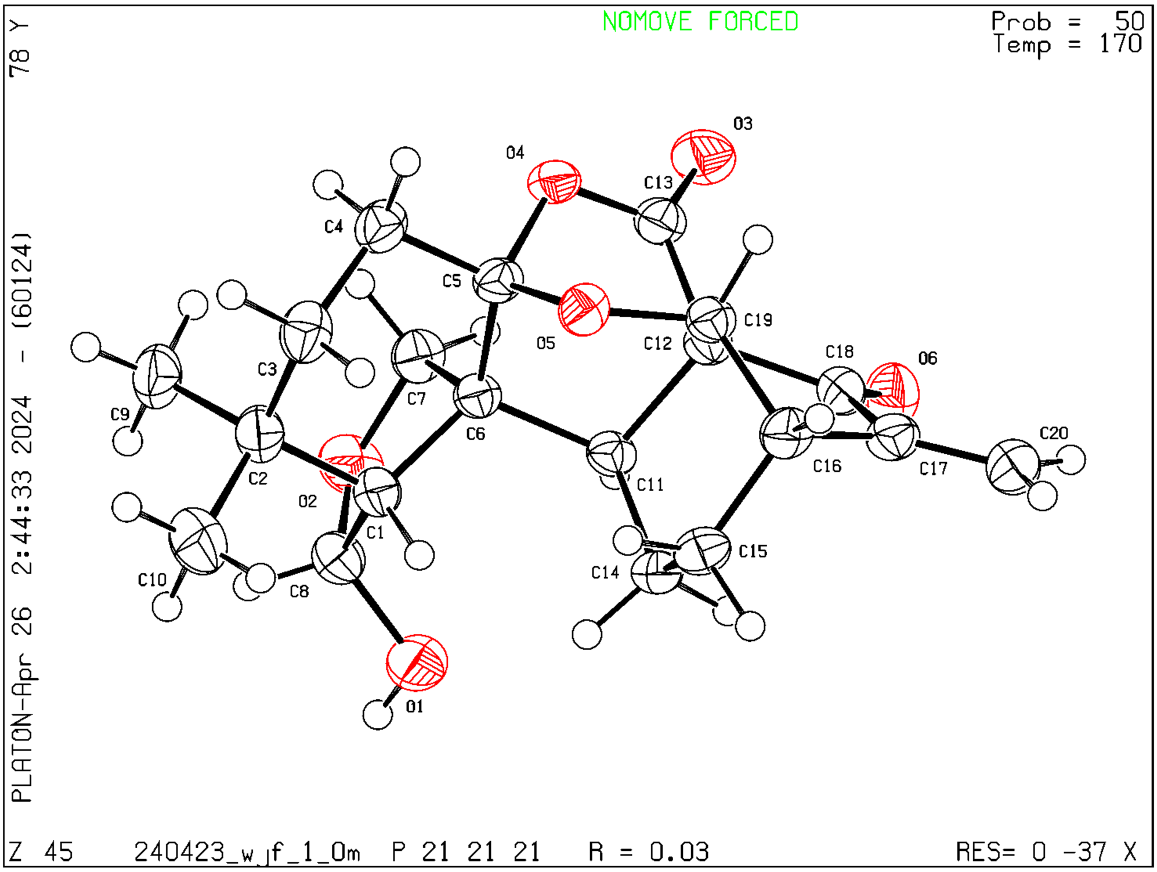

Supplement: Supplementary file 1 [file molecules-29-04066-s001.zip › molecules-3152720-supplementary/Supplemental material/The single-crystal data of compound 4/checkcif.pdf]
