# Supplementary figures and images for: Design, Synthesis, and Anticancer Activity of Novel Enmein-Type Diterpenoid Derivatives Targeting the PI3K/Akt/mTOR Signaling Pathway
Source: Molecules. 2024 Aug 27;29(17):4066. doi: 10.3390/molecules29174066 (PMC11396751; doi:10.3390/molecules29174066)

Control


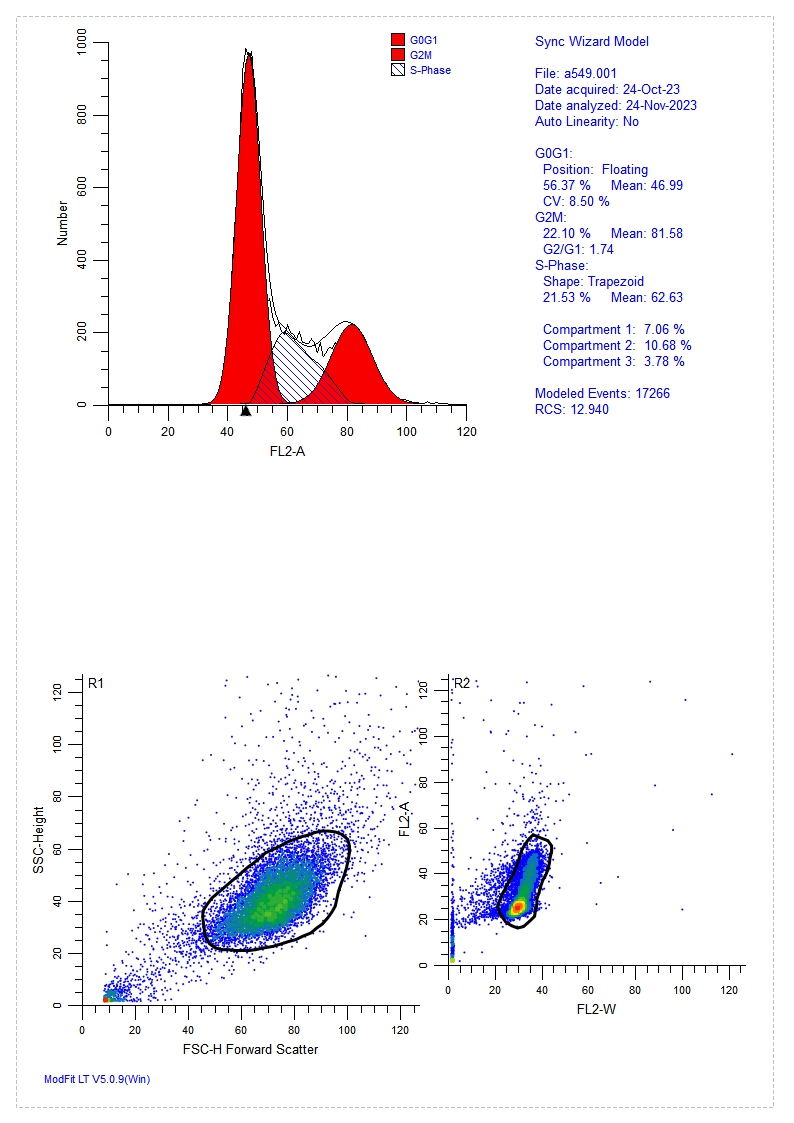


2 µM


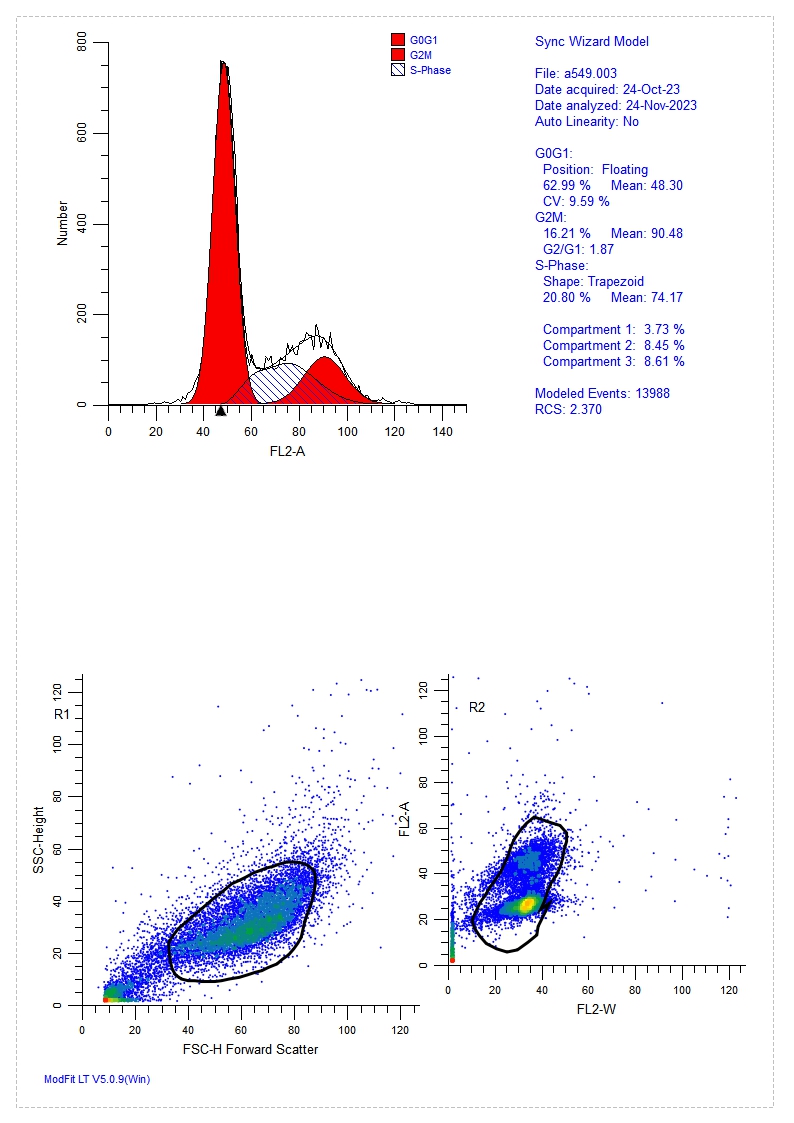


4 µM


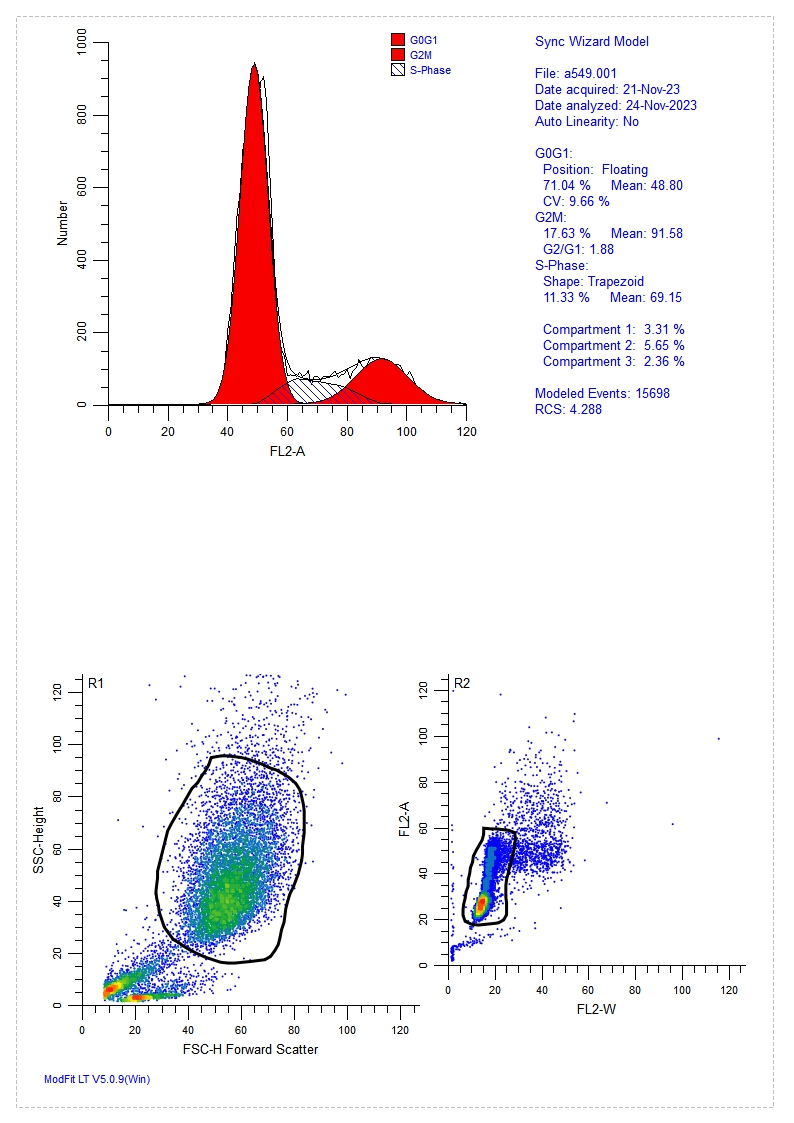


8 µM


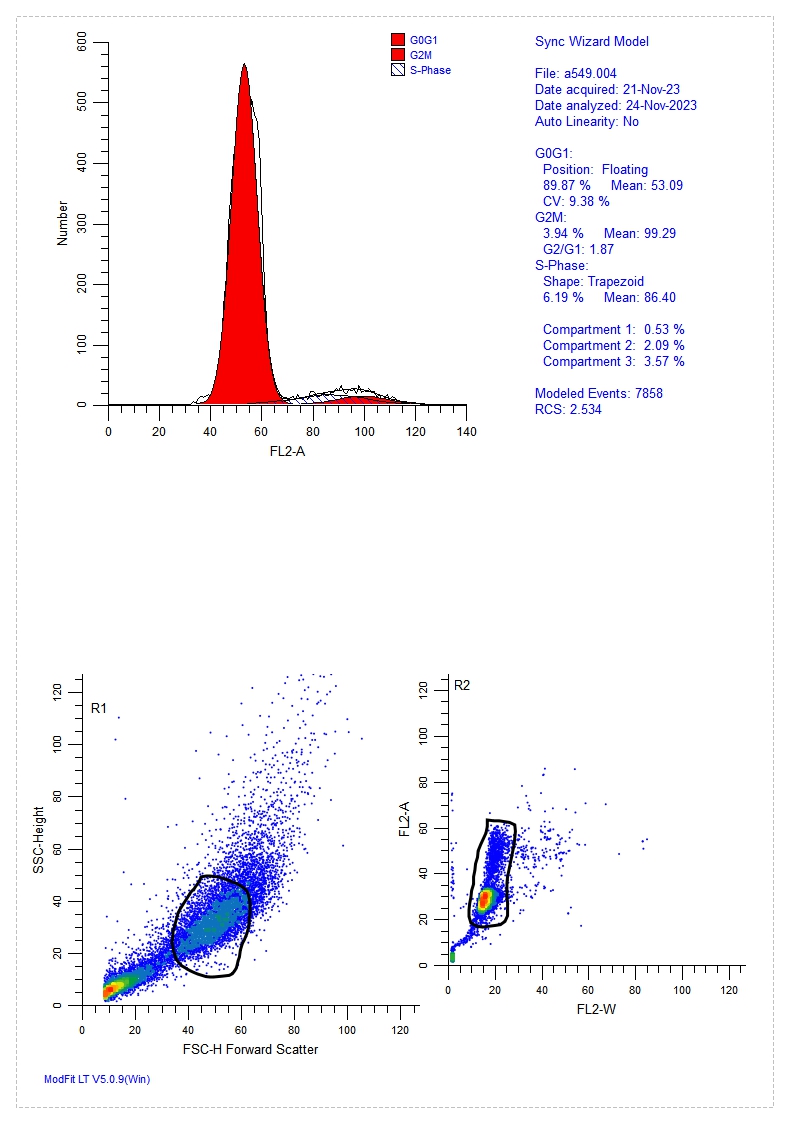

Supplement: Supplementary file 1 [file molecules-29-04066-s001.zip › molecules-3152720-supplementary/Supplemental material/Original Image/Original Image for Figure 3.docx]

Control


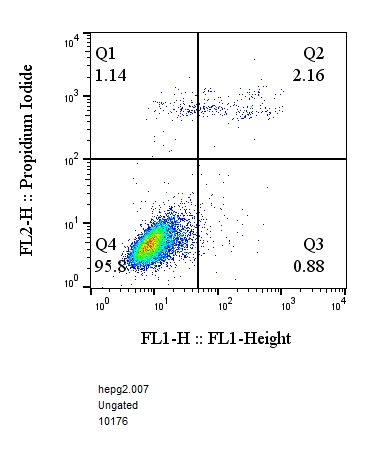


2 µM


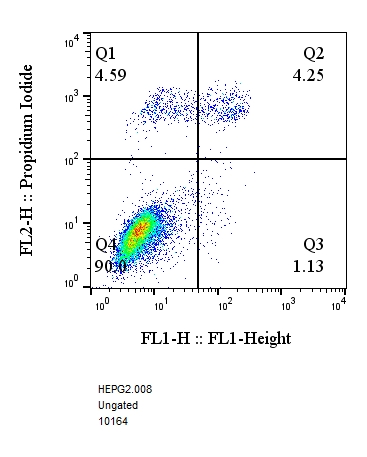


4 µM


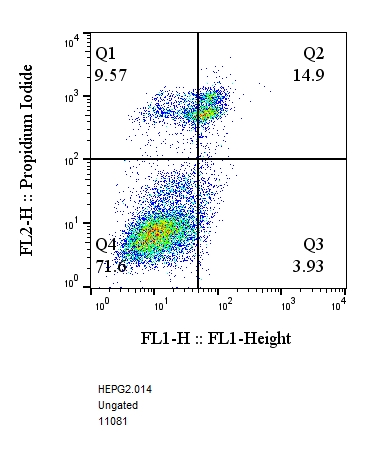


8 µM


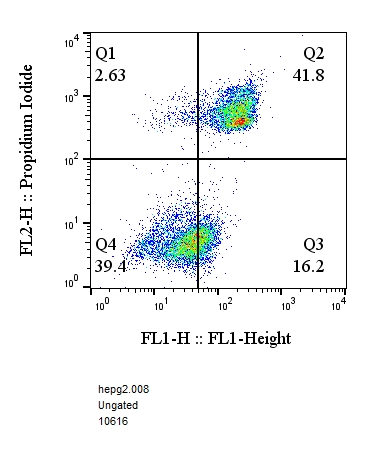


Control 2 µM 4 µM 8 µM


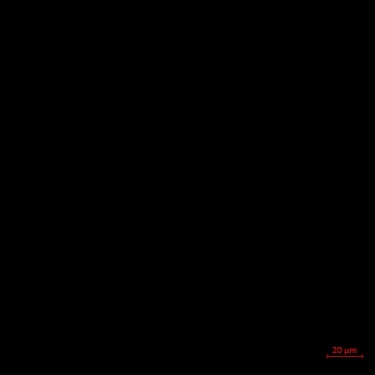

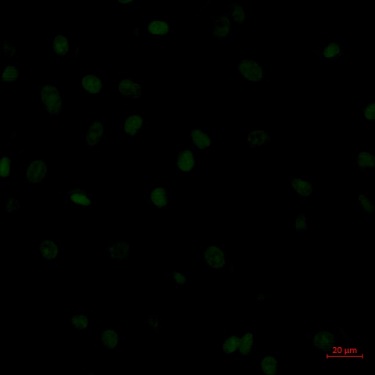

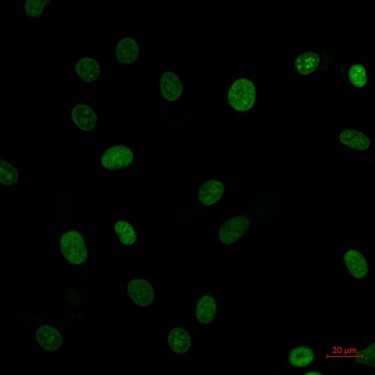

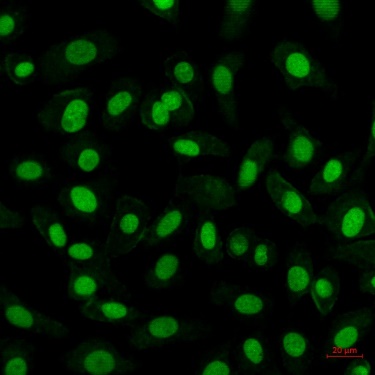


AO


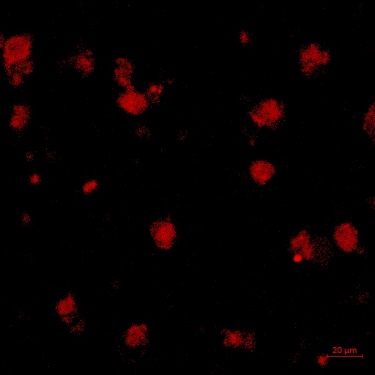

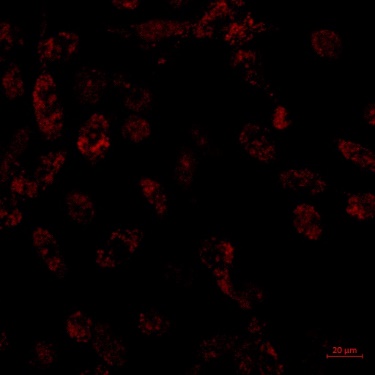

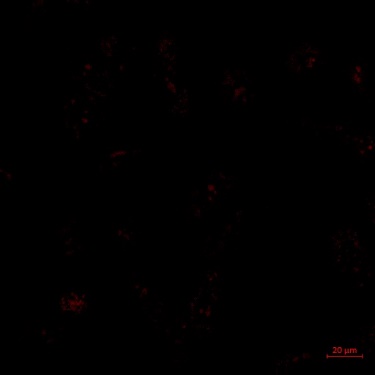

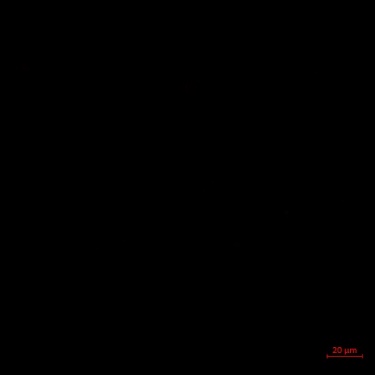


EB


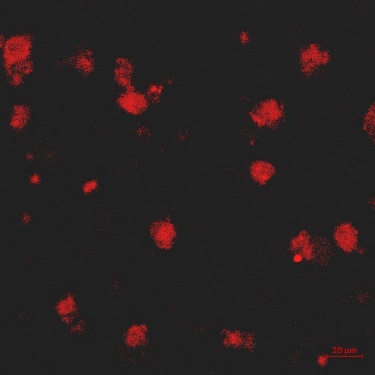

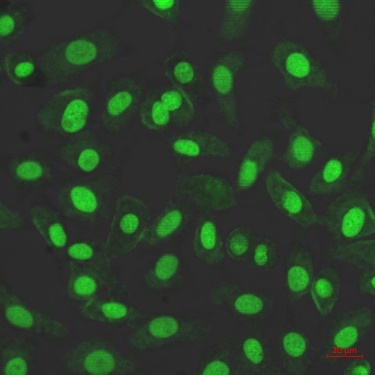

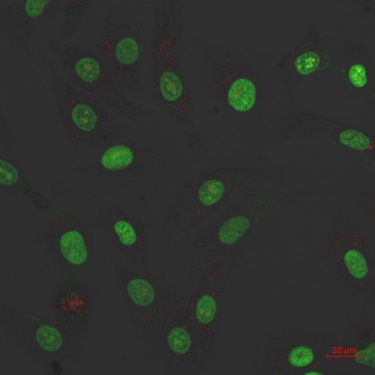

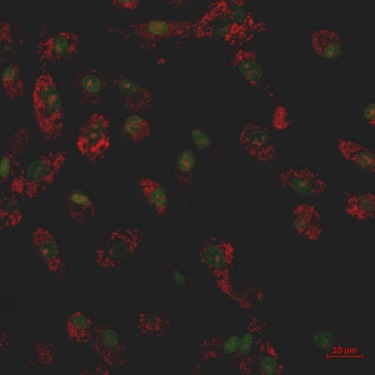


Merge

Supplement: Supplementary file 1 [file molecules-29-04066-s001.zip › molecules-3152720-supplementary/Supplemental material/Original Image/Original Image for Figure 4.docx]

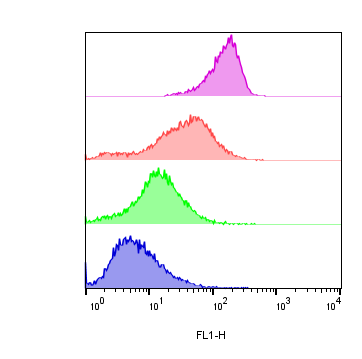


Control 2 µM 4 µM 8 µM


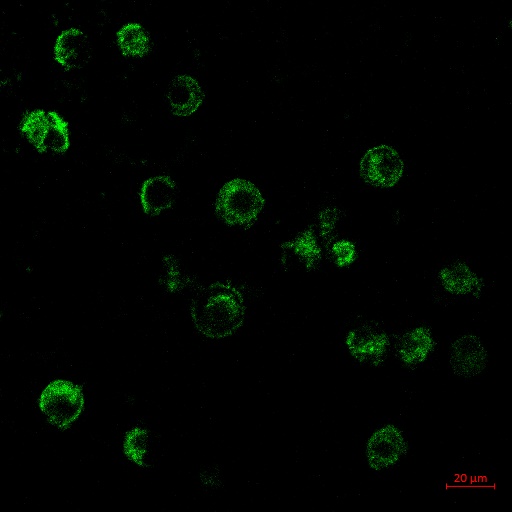

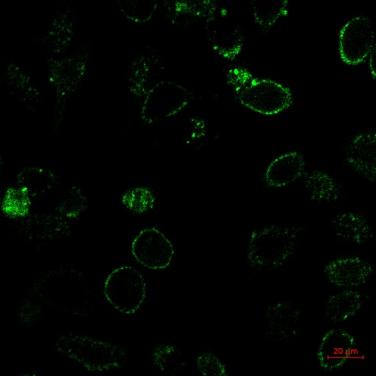

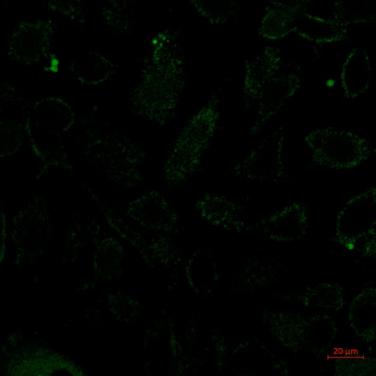

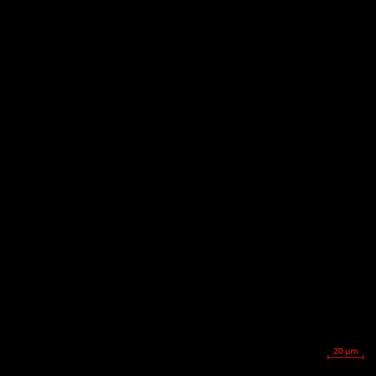


ROS


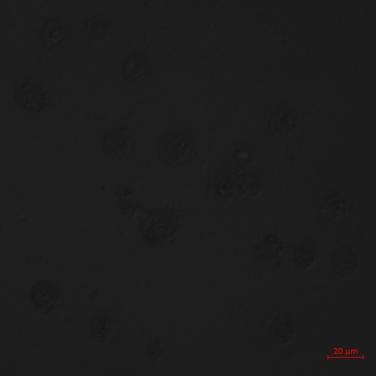

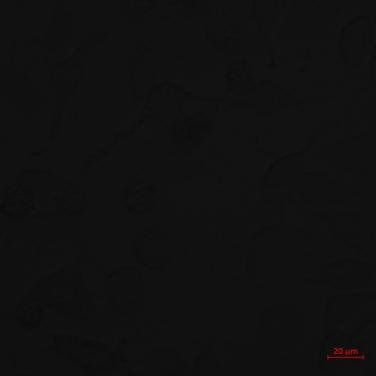


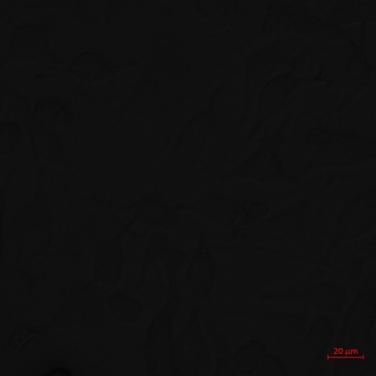

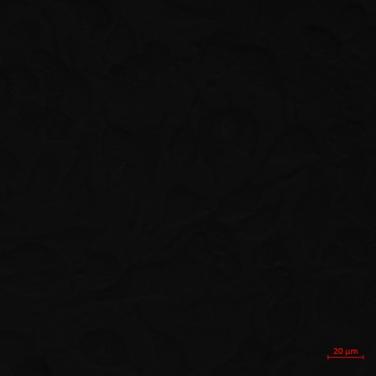


Bright


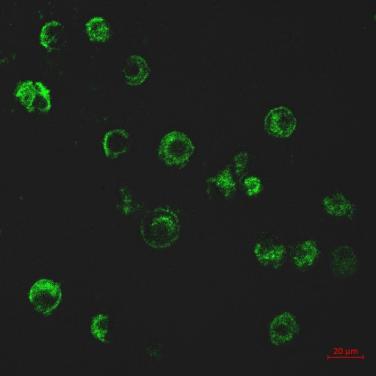

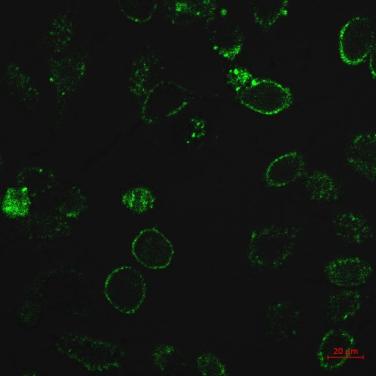

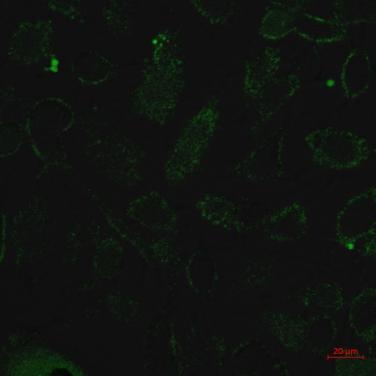

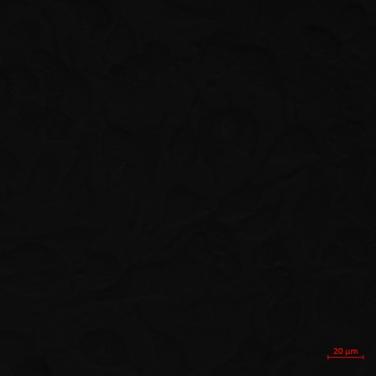


Merge

Supplement: Supplementary file 1 [file molecules-29-04066-s001.zip › molecules-3152720-supplementary/Supplemental material/Original Image/Original Image for Figure 5.docx]

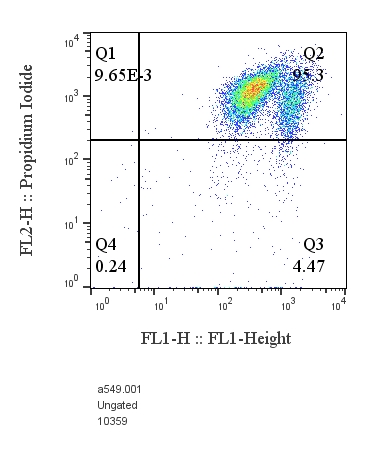
Control 2 µM


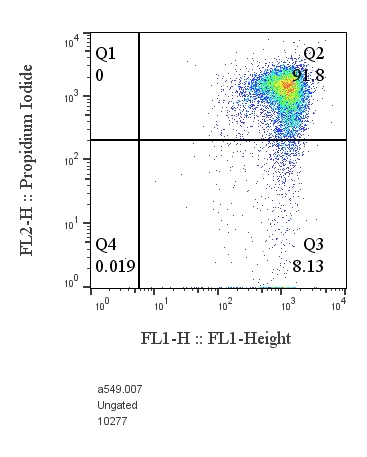


2 µM

4 µM

4 µM 8 µM


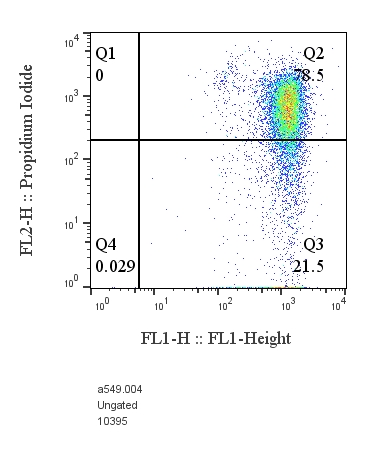

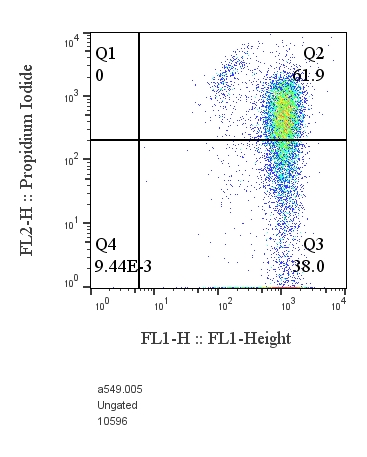


Control 2 µM 4 µM 8 µM


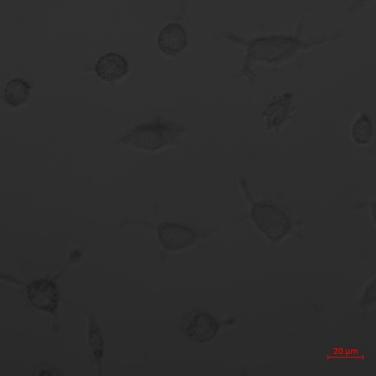

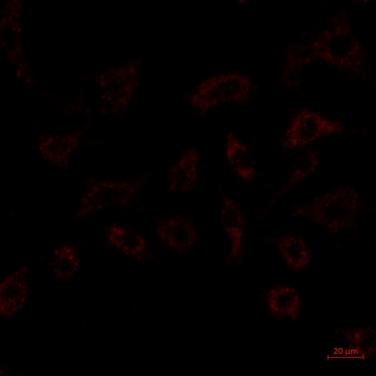

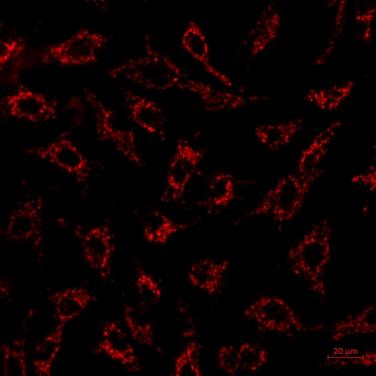

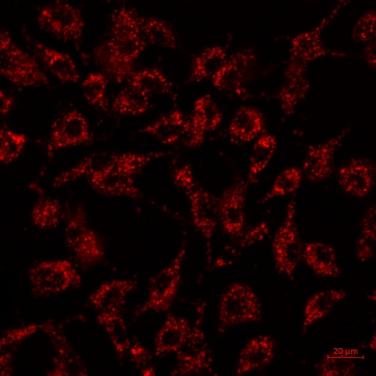


JC-1 aggregate


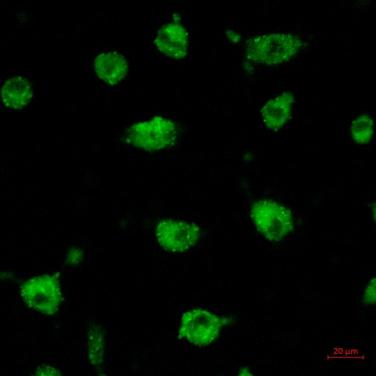

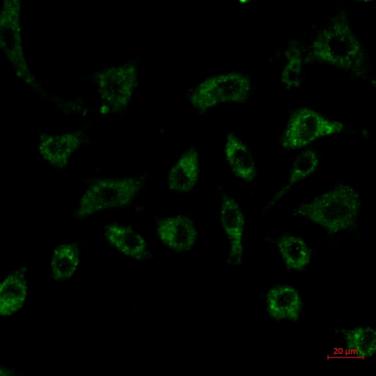

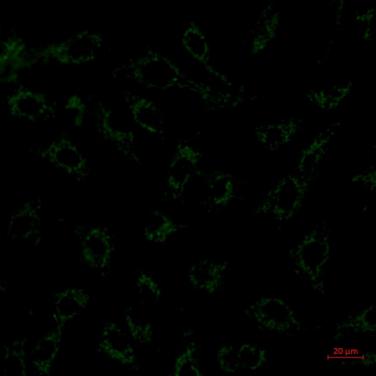

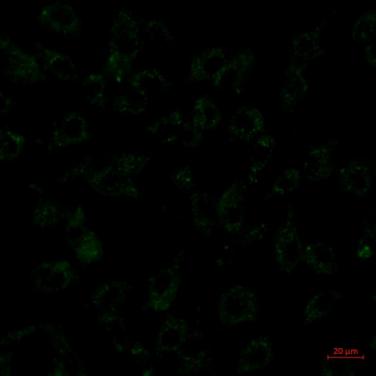


JC-1 monomer


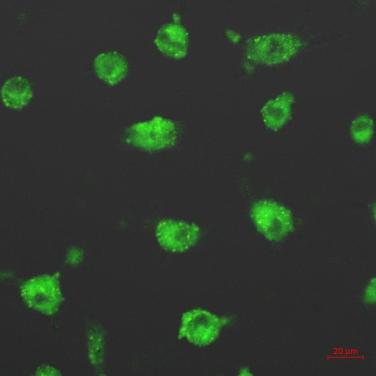

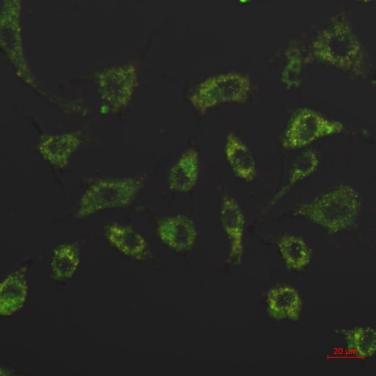

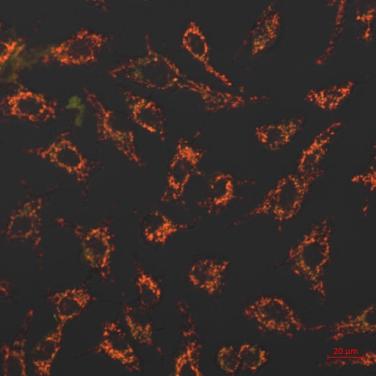

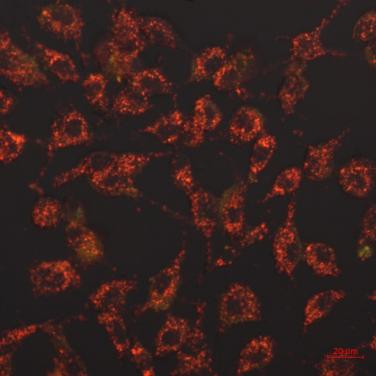


Merge

Supplement: Supplementary file 1 [file molecules-29-04066-s001.zip › molecules-3152720-supplementary/Supplemental material/Original Image/Original Image for Figure 6.docx]

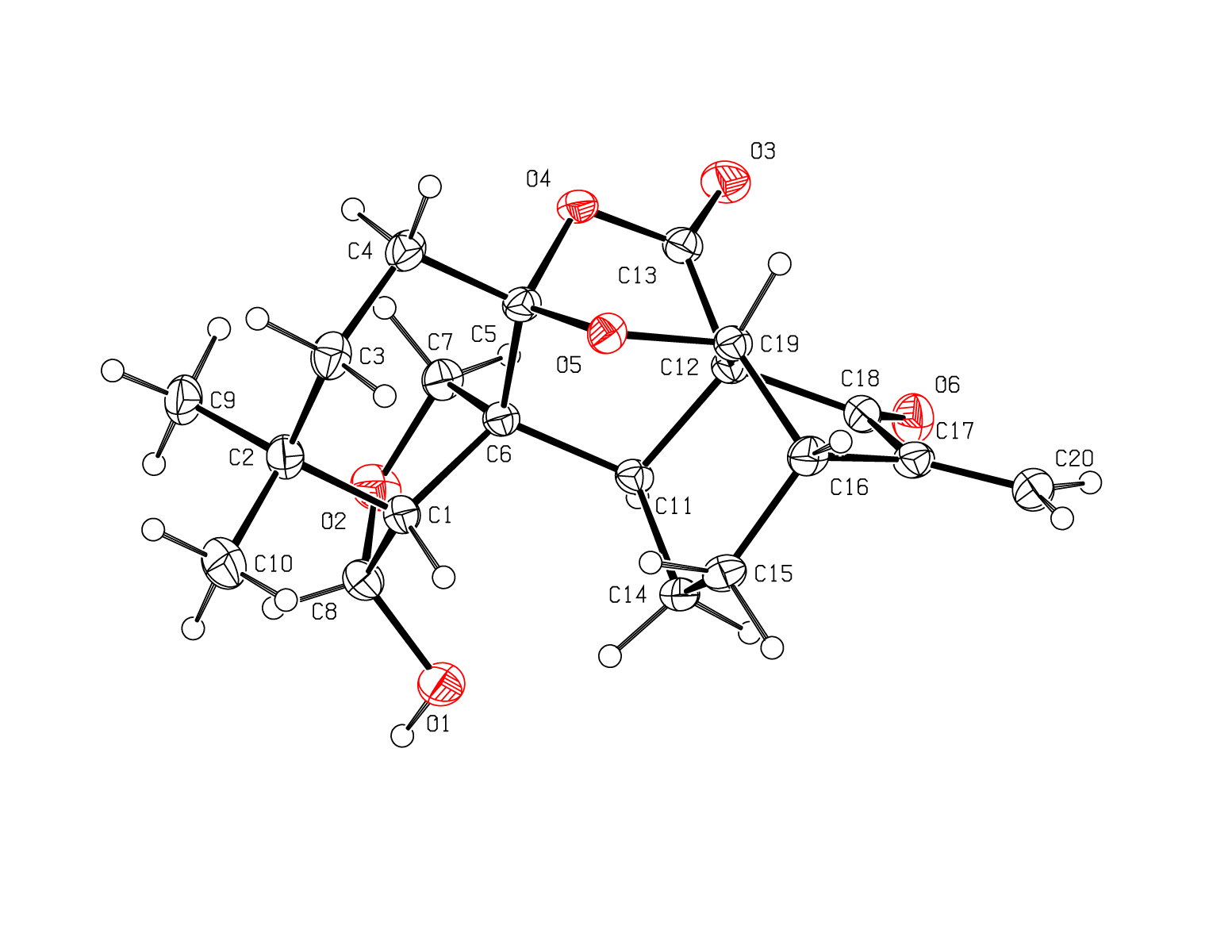

Supplement: Supplementary file 1 [file molecules-29-04066-s001.zip › molecules-3152720-supplementary/Supplemental material/The single-crystal data of compound 4/240423_WJF_1_0m.tif]
